# 4-1BB-encoding CAR causes cell death via sequestration of the ubiquitin-modifying enzyme A20

**DOI:** 10.1038/s41423-024-01198-y

**Published:** 2024-06-27

**Authors:** Zhangqi Dou, Thomas Raphael Bonacci, Peishun Shou, Elisa Landoni, Mark G. Woodcock, Chuang Sun, Barbara Savoldo, Laura E. Herring, Michael J. Emanuele, Feifei Song, Albert S. Baldwin, Yisong Wan, Gianpietro Dotti, Xin Zhou

**Affiliations:** 1 Lineberger Comprehensive Cancer Center, University of North Carolina, Chapel Hill, NC USA; 2 Department of Neurosurgery, Second Affiliated Hospital, School of Medicine, Zhejiang University, Hangzhou, Zhejiang China; 3 Department of Genetics, University of North Carolina, Chapel Hill, NC USA; 4 Division of Oncology, Department of Medicine, The University of North Carolina at Chapel Hill, Chapel Hill, NC USA; 5 Department of Pediatrics, University of North Carolina, Chapel Hill, NC USA; 6 Michael Hooker Proteomics Center, Department of Pharmacology, University of North Carolina, Chapel Hill, NC USA; 7 Department of Microbiology and Immunology, University of North Carolina, Chapel Hill, NC USA

**Keywords:** Chimeric antigen receptor (CAR)-T cell, 4-1BB, Necroptosis, A20, NF-κB, TNF receptor associated factor (TRAF), Cancer immunotherapy, Cancer immunotherapy

## Abstract

CD28 and 4-1BB costimulatory endodomains included in chimeric antigen receptor (CAR) molecules play a critical role in promoting sustained antitumor activity of CAR-T cells. However, the molecular events associated with the ectopic and constitutive display of either CD28 or 4-1BB in CAR-T cells have been only partially explored. In the current study, we demonstrated that 4-1BB incorporated within the CAR leads to cell cluster formation and cell death in the forms of both apoptosis and necroptosis in the absence of CAR tonic signaling. Mechanistic studies illustrate that 4-1BB sequesters A20 to the cell membrane in a TRAF-dependent manner causing A20 functional deficiency that in turn leads to NF-κB hyperactivity, cell aggregation via ICAM-1 overexpression, and cell death including necroptosis via RIPK1/RIPK3/MLKL pathway. Genetic modulations obtained by either overexpressing A20 or releasing A20 from 4-1BB by deleting the TRAF-binding motifs of 4-1BB rescue cell cluster formation and cell death and enhance the antitumor ability of 4-1BB-costimulated CAR-T cells.

## Introduction

CAR-T cells have been proven to be a promising cancer immunotherapy in some hematologic malignancies [1–6]. CAR-T cell costimulation provided by either CD28 or 4-1BB endodomains plays a fundamental role in promoting CAR-T cell therapeutic effects [7–9]. However, mechanistic studies elucidating the molecular effects of CD28 and 4-1BB included in the CAR as fusion proteins remain limited. Understanding how the ectopic and constitutive expression of costimulatory endodomains affect their interaction with other signaling molecules remains critical to optimize the design of CAR signaling.

We and others reported that CAR molecules carrying either CD28 or 4-1BB endodomains show significantly different patterns of phosphorylation [8–10]. Specifically, CD28 within the CAR promotes LCK-mediated phosphorylation of the CD3ζ-chain, while the THEMIS/SHP1 phosphate complex recruited by 4-1BB attenuates the CD3ζ-chain phosphorylation [8]. These signaling differences could explain at least in part why CD28 costimulation mediates rapid tumor-killing of CAR-T cells, while 4-1BB causes relatively slow antitumor effects [8, 9]. 4-1BB costimulation of CAR-T cells has also been associated with better T cell persistence, metabolic fitness, and memory formation compared to CD28-costimulated CAR-T cells [8, 9, 11]. However, 4-1BB can also increase CD95/FAS-mediated T-cell apoptosis [12]. It remains to be determined if these biological effects are caused by specific and not yet investigated molecular interactions of CD28 and 4-1BB expressed within the CAR with other molecules.

In the current study, we demonstrated that 4-1BB endodomain causes cell aggregation and cell death in the forms of both apoptosis and necroptosis in CAR-T cells. Furthermore, we provide mechanistic data linking cell aggregation and cell death caused by 4-1BB to the NF-κB/A20 pathway.

## Results

### 4-1BB signaling causes CAR-T cell aggregation and cell death

**Fig. 1:**
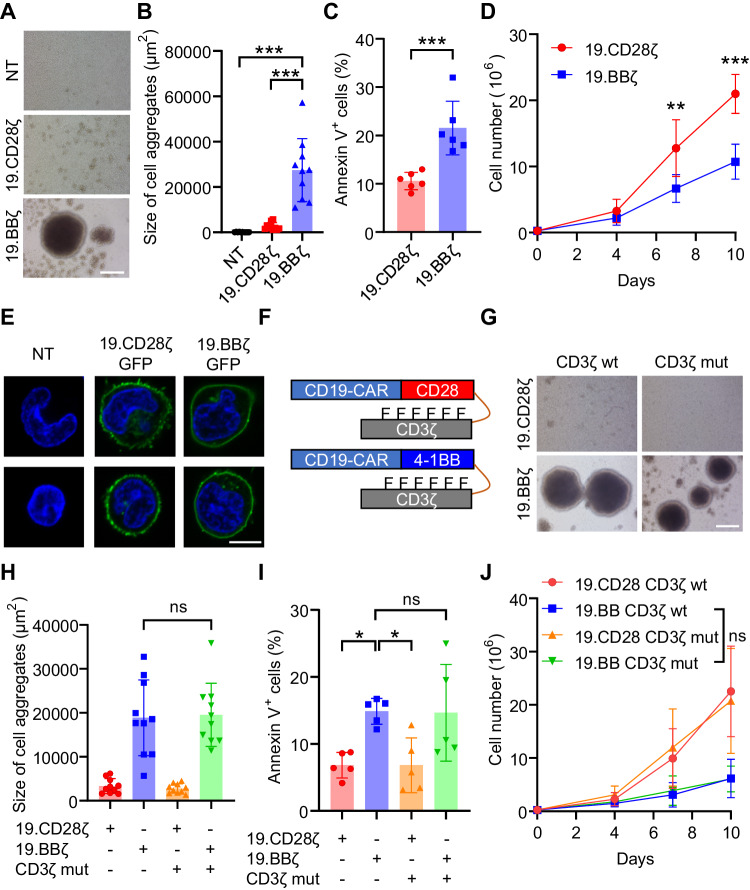
4-1BB signaling causes cell aggregation and Annexin-V expression in CAR-T cells. **A** Representative microscopic imaging showing the formation of cell aggregates in NT, 19.CD28ζ, and 19.BBζ CAR-T cells at day 7–10 during the in vitro expansion; magnification 20×; scale bar 100 µm. NT indicates control non-transduced T cells. **B** Quantification of the size of cell aggregates illustrated in (**A**); *n* = 10, ***p < 0.001, one-way ANOVA. **C** Quantification of Annexin-V^+^ cells of 19.CD28ζ and 19.BBζ CAR-T cells at day 7–10 of culture; *n* = 6, ***p < 0.001, *t*-test. **D** Cell counts of 19.CD28ζ and 19.BBζ CAR-T cells in vitro. Cell numbers were counted by flow cytometry with counting beads; *n* = 4, **p < 0.01, ***p < 0.001, two-way ANOVA. **E** Representative confocal microscopy imaging showing the distribution of the GFP-tagged 19.CD28ζ and 19.BBζ CARs on the cell surface of CAR-T cells. Blue staining indicates DAPI. Shown are representative cells of a single field; magnification 63×; scale bar 5 µm. **F** Schematic of the 19.CD28ζ and 19.BBζ CARs with loss-of-function mutation in the ITAMs (CD3ζ mut); F represents phenylalanine. **G** Representative microscopic imaging showing the formation of cell aggregates in 19.CD28ζ and 19.BBζ CAR-T cells, and 19.BB CD3ζ mut CAR-T cells at day 7–10 during the in vitro expansion; magnification 20×; scale bar 100 µm. **H** Quantification of the size of cell aggregates illustrated in (**G**); *n* = 10, ns represents no significance. **I** Quantification of Annexin-V^+^ cells of 19.CD28ζ and 19.BBζ CAR-T cells, and 19.BB CD3ζ mut CAR-T cells at day 7–10 days of culture; *n* = 5, *p < 0.05, ns represents no significance, one-way ANOVA. **J** Cell counts of 19.CD28ζ and 19.BBζ CAR-T cells, and 19.BB CD3ζ mut CAR-T cells in vitro. Cell numbers were counted by flow cytometry with counting beads; *n* = 4, ns represents no significance at day 10, two-way ANOVA

We have previously constructed CD19-specific CARs encoding the CD8α stalk with either the CD28 (19.CD28ζ) or 4-1BB (19.BBζ) endodomains and expressed these CARs via gamma retroviral gene transfer in activated human T cells [8]. CARs are equally expressed in T cells as assessed by flow cytometry at 7–10 days of culture (Fig. S1A). However, 19.BBζ CAR-T cells showed the formation of large cell clusters (Fig. 1A, B), more Annexin-V^+^ cells (Fig. 1C and Fig. S1B), and low T-cell counts compared to 19.CD28ζ CAR-T cells (Fig. 1D). To explore whether the effects observed in 19.BBζ CAR-T cells are caused by self-aggregation of the CAR known as tonic signaling [13, 14], we compared the CAR biodistribution on the cell surface of T cells by confocal microscopy using GFP-tagged 19.CD28ζ and 19.BBζ CARs, as previously described [15]. GFP-tagging of the CARs did not alter the formation of cell aggregates and the frequency of Annexin-V^+^ cells noted in 19.BBζ CAR-T cells (Fig. S1C–E). Both 19.BBζGFP and 19.CD28ζGFP CARs showed similar biodistribution on the cell surface (Fig. 1E). In addition, they showed similar basal levels of spontaneous secretion of IFNγ and IL-2 (Fig. S1F), which is also a marker of tonic signaling [14]. To assess if the effects observed in 19.BBζ CAR-T cells are scFv specific, we tested two additional CARs generated using scFvs specific for the GD2 (scFv 14G2a) or B7-H3 (scFv 376.96) antigens [16, 17]. T cells expressing the 4-1BB costimulated GD2.CAR or B7-H3.CAR showed more cell aggregates and more Annexin-V^+^ cells, and lower T-cell counts compared to the CD28-costimulated CAR-T cells (Fig. S2A–F). We then evaluated whether the formation of cell aggregates of 19.BBζ CAR-T cells was dependent on the CD3ζ chain that initiates CAR signaling upon antigen engagement. We mutated six tyrosine of the CD3ζ immunoreceptor tyrosine-based activation motifs (ITAMs) [18] to phenylalanine (Fig. 1F). As expected, CAR-T with the mutated CD3ζ showed impaired cell proliferation upon activation and impaired antitumor ability (Fig. S2G, H). However, no significant decrease was detected in the formation of cell aggregates (Fig. 1G, H), and 19.BB CAR-T cells carrying the mutated CD3ζ ITAMs continued to show more Annexin-V^+^ cells and reduced cell counts (Fig. 1I, J). To further explore the relationship between cell aggregation and Annexin-V expression observed in 19.BBζ CAR-T cells, we first examined the expression of Annexin-V in single cells, cells in clusters, and single cells obtained after gentle disaggregation of the cell clusters. We did not observe a significant difference in Annexin-V expression in all three conditions (Fig. S3A, B). Since ICAM-1 has been reported to mediate cell aggregation [19], we detected the expression of ICAM-1 in 19.BBζ and 19.CD28ζ CAR-T cells. 19.BBζ CAR-T cells displayed higher expression of ICAM-1, and cells in clusters showed significantly higher ICAM-1 expression than single cells (Fig. S3C, D). Moreover, blocking ICAM-1 signaling by incubating 19.BBζ CAR-T cells with the ICAM-1 Ab significantly reduced cell cluster formation (Fig. S3E, F). Overall, these data suggest that 4-1BB signaling causes CAR-T cell aggregation mediated by ICAM-1 expression and increases the frequency of Annexin-V^+^ cells. These phenomena are not caused by the scFv self-aggregation or CD3ζ chain signaling.

### 4-1BB costimulation induces apoptosis and necroptosis

**Fig. 2:**
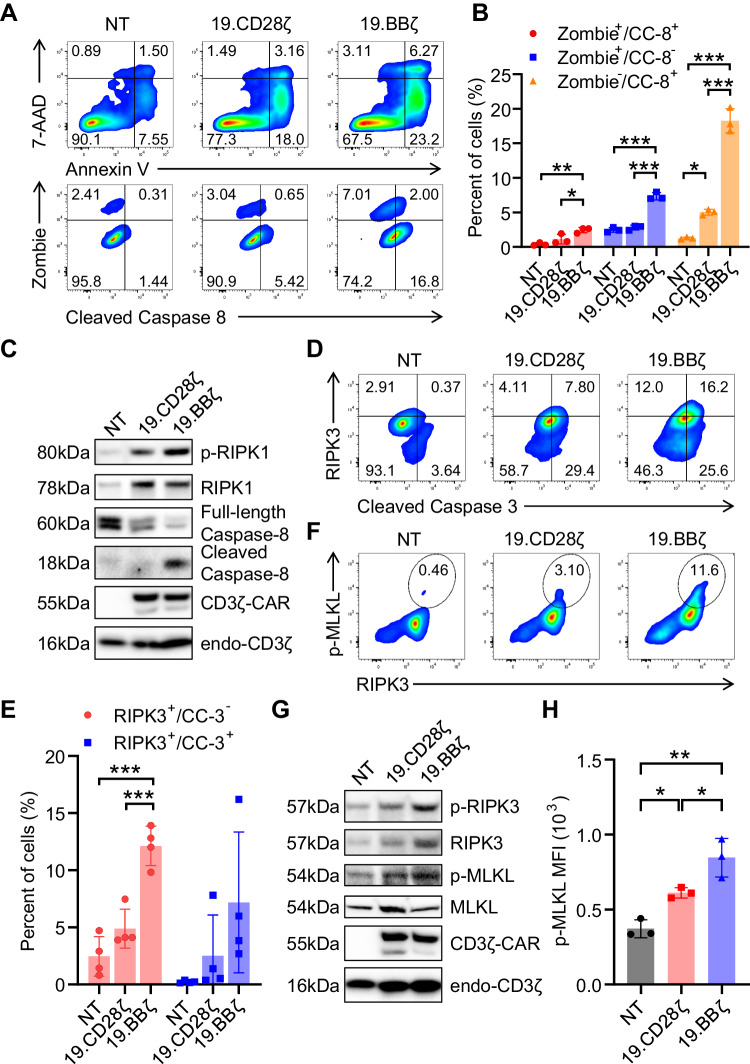
4-1BB costimulation induces apoptosis and necroptosis in CAR-T cells. **A** Representative flow cytometry plots indicating the cell frequency of Annexin-V^+^/7-AAD^−^ and Annexin-V^+^/7-AAD^+^ in NT, 19.CD28ζ and 19.BBζ CAR-T cells (upper panel) and the frequency of Zombie^+^/cleaved caspase-8^−^, Zombie^+^/cleaved caspase-8^+^, and Zombie^−^/cleaved caspase-8^+^ in NT, 19.BBζ and 19.CD28ζ CAR-T cells (lower panel); NT indicates control non-transduced T cells. **B** Quantification of the cells illustrated in (**A**); *n* = 3, *p < 0.05, **p < 0.01, ***p < 0.001, one-way ANOVA; CC-8 represents the cleaved caspase-8. **C** Western blot analysis showing the expression of the cleaved caspase-8 and phosphorylation of RIPK1 (p-RIPK1, Ser^166^) in NT, 19.CD28ζ, and 19.BBζ CAR-T cells. **D** Representative flow cytometry plots showing RIPK3^+^cleaved caspase-3^−^ and RIPK3^+^cleaved caspase-3^+^ cells in NT, 19.CD28ζ and 19.BBζ CAR-T cells, respectively. **E** Quantification of the cells illustrated in (**D**); *n* = 4; ***p < 0.001, one-way ANOVA; CC-3 represents the cleaved caspase-3. **F** Representative flow cytometry plots showing the double staining of RIPK3 and p-MLKL in NT, 19.CD28ζ, and 19.BBζ CAR-T cells. **G** Representative western blot showing the expression of phosphorylated RIPK3 (p-RIPK3, Ser^227^) and phosphorylated MLKL (p-MLKL, Ser^358^) in NT, 19.CD28ζ, and 19.BBζ CAR-T cells. **H** Statistics of the mean fluorescence intensity (MFI) illustrating higher levels of p-MLKL in 19.BBζ compared to 19.CD28ζ CAR-T cells; *p < 0.05, **p < 0.01, one-way ANOVA

Since Annexin-V expression correlates with cell apoptosis and necroptosis [20, 21], to better characterize the Annexin-V^+^ CAR-T cells we performed double staining with Annexin-V and 7-AAD in 19.BBζ and 19.CD28ζ CAR-T cells growing in culture. We found higher Annexin-V^+^/7-AAD^−^ (early apoptosis) cells and higher Annexin-V^+^/7-AAD^+^ (late apoptosis/necrosis) cells in 19.BBζ CAR-T cells compared to 19.CD28ζ CAR-T cells (Fig. 2A). Consistently, higher levels of cleaved caspase-8 and phosphorylation of RIPK1 were detected in 19.BBζ CAR-T cells compared to 19.CD28ζ CAR-T cells indicating increased apoptosis [22] (Fig. 2A–C). In addition, we also observed the presence of Zombie^+^ cells independent from cleaved caspase-8, which indicates the presence of other types of cell death (Fig. 2A, B). 19.BBζ CAR-T cells showed increased RIPK3^+^/cleaved caspase-3^−^ cells compared to 19.CD28ζ CAR-T cells suggesting the induction of cell necroptosis [22] (Fig. 2D, E). The occurrence of necroptosis in 19.BBζ CAR-T cells was confirmed by the detection of higher expression of the phosphorylated form of RIPK3 and MLKL (Fig. 2F–H and Fig. S4A). Furthermore, incubation of 19.BBζ CAR-T cells with necrostatin-1 - a well-described RIPK1 inhibitor - reduced the number of Annexin-V^+^ cells (Fig. S4B) [23]. Since T cell activation causes induced cell death [24], we also analyzed if CAR-T cell stimulation causes different patterns of cell death in 19.CD28ζ and 19.BBζ CAR-T cells. In contrast to the observation of CAR-T growing in culture, we found that CAR-T activation caused similar activation-induced cell death in 19.CD28ζ and 19.BBζ CAR-T cells (Fig. S4C–F). Overall, these data indicate that 4-1BB endodomain in CAR-T cells causes cell death in the forms of apoptosis and necroptosis during the in vitro culture of the cells in the absence of CAR stimulation and activation-induced cell death.

### 4-1BB/TRAF mediates cell aggregation and cell death via NF-κB

**Fig. 3:**
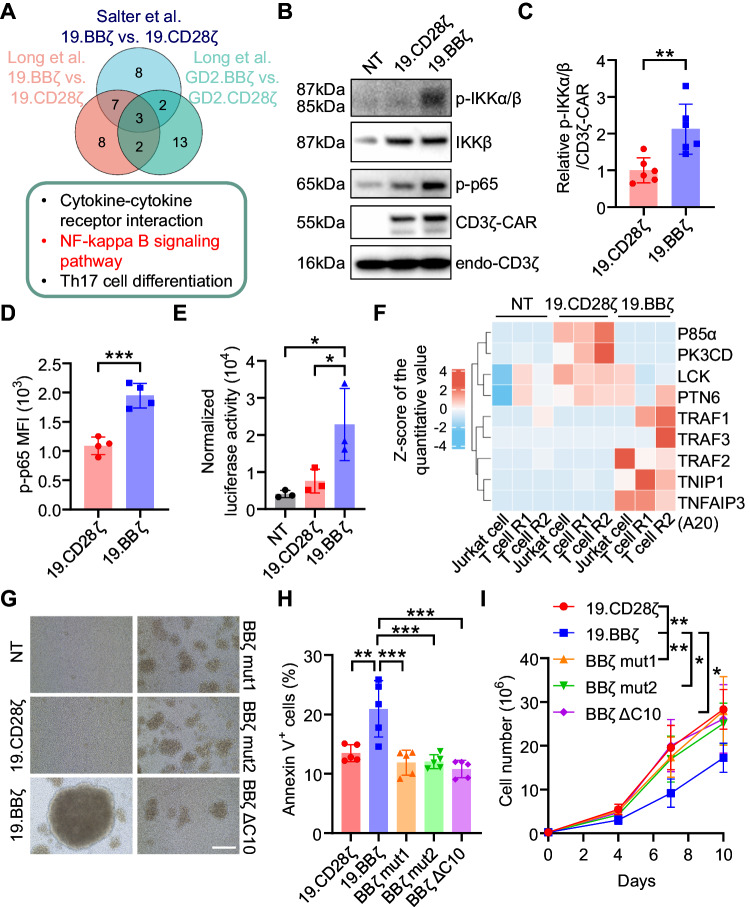
4-1BB causes hyperactive NF-κB signaling and 4-1BB-mediated cell death requires TRAF. **A** Venn plot illustrating the number of KEGG pathways enriched in three RNAseq or microarray datasets comparing 4-1BB to CD28-costimulated CAR-T cells (upper panel) and the detail of three shared KEGG pathways (lower panel, NF-κB pathway marked as red). **B** Representative western blot showing the phosphorylation of IKKα/β (p-IKKα/β, Ser^176/180^) and p65 (p-p65, Ser^536^) in 19.CD28ζ and 19.BBζ CAR-T cells. NT indicates control non-transduced T cells. **C** Quantification of p-IKKα/β normalized to CD3ζ-CAR in NT, 19.CD28ζ and 19.BBζ CAR-T cells in the western blots shown in (**B**); *n* = 6; **p < 0.01, one-way ANOVA. **D** Quantification of the p-p65 expression as mean fluorescence intensity (MFI) in 19.CD28ζ and 19.BBζ CAR-T cells; *n* = 4, ***p < 0.001, *t*-test. **E** Quantification of the NF-κB luciferase activity in NT, 19.CD28ζ and 19.BBζ CAR-T cells. CAR-T cells were transduced with an NF-κB luciferase reporter and collected after 72 h to measure the luciferase activity. Luciferase activity was normalized by genomic luciferase level; *n* = 3, *p < 0.05, one-way ANOVA. **F** Clustering heatmap of proteomics analysis obtained by CoIP-LC-MS/MS showing the detection of peptides in the CAR pull-down from either Jurkat cells or T cells expressing either the 19.CD28ζ CAR or the 19.BBζ CAR. NT indicates control of non-transduced Jurkat cells and non-transduced T cells. **G** Representative microscopic imaging showing the formation of cell aggregates of CAR-T cells expressing either the 19.CD28ζ or the 19.BBζ CARs and CAR-T cells expressing the mutated form of the 4-1BB endodomain (BBζ mut1, BBζ mut2, and BBζ ΔC10). NT indicates control non-transduced T cells; magnification 20×; scale bar 100 µm. **H** Quantification of Annexin-V^+^ cells of the CAR-T cells described in (**G**) at day 7–10 of culture; *n* = 5, **p < 0.01, ***p < 0.001, one-way ANOVA. **I** Cell counts of the CAR-T cells described in (**G**). Cell numbers were counted by flow cytometry with counting beads; *n* = 4, *p < 0.05, **p < 0.01 at day 10, two-way ANOVA

To explore the mechanism leading to cell aggregation and cell death in 4-1BB costimulated CAR-T cells, we first analyzed available RNAseq (GSE109161) [10] and microarray (GSE65856) [13] datasets comparing 4-1BB and CD28-costimulated CAR-T cells. The most striking and consistent result of this comparison was the enrichment of the NF-κB pathway in 4-1BB costimulated CAR-T cells compared to CD28-costimulated CAR-T cells according to the top 20 KEGG pathways in all analyzed datasets (Fig. 3A and Fig. S5A). Since NF-κB signaling can lead to ICAM-1 expression and cell adhesion [25], hyperactive NF-κB pathway in 4-1BB-costimulated CAR-T cells could explain the cell aggregation. In contrast, the role of the NF-κB pathway in promoting cell death remains controversial since NF-κB is in general considered a pro-survival signal [26]. We evaluated the NF-κB signaling in 19.BBζ and 19.CD28ζ CAR-T cells at day 7–10 of culture and found that NF-κB pathway is hyperactive in 4-1BB costimulated CAR-T cells. Specifically, we found higher phosphorylation of IKKα/β, a key signaling event of the canonical NF-κB pathway [27] (Fig. 3B, C), as well as p65 (Fig. 3D and Fig. S5B) in 19.BBζ compared to 19.CD28ζ CAR-T cells. Higher NF-κB activity in 19.BBζ CAR-T cells was also observed using a specific NF-κB reporter assay (Fig. 3E). Similar hyperactivity of the NF-κB pathway was observed in T cells expressing either the GD2-specific or the B7-H3-specific CARs encoding 4-1BB (Fig. S5B–D). We also found that hyperactive NF-κB in 19.BBζ CAR-T cells is strictly 4-1BB-dependent and CD3ζ-independent, because loss-of-function of the CD3ζ ITAMs did not abrogate the NF-κB signaling (Fig. S5E). We performed co-immunoprecipitation-mass spectrometry analysis (CoIP-LC-MS/MS) on both 19.CD28ζ and 19.BBζ CARs pull-down obtained from CAR-T cells and CAR-expressing Jurkat cells. We found that the adapter p85α subunit of the PI3K involved in the CD28 signaling was more abundant in the 19.CD28ζ pull-down, while TRAF family members including TRAF1/2/3 were more abundant in the 19.BBζ pull-down, which plays important roles in the NF-κB pathway (Fig. 3F) [28, 29]. We then mutated 4-1BB to remove either the TRAF2 binding site (BBζ mut1, QEED→QAAA) or the TRAF1/2/3 binding sites (BBζ mut2, PEEEE→PEAAA), or deleted the last 10 amino acids of the carboxyl-terminal tail (BBζ ΔC10) (Fig. S6A) [8, 12, 28, 30]. In T cells expressing all three 4-1BB CAR mutants, we observed the decrease of p-IKKα/β and p-p65 levels under the premise that the CAR expression and cell proliferation upon activation were not affected (Fig. S6B–E). T cells expressing the three 4-1BB CAR mutants showed significantly reduced formation of cell clusters and reduced ICAM-1 expression underlining the role of NF-κB signaling in causing cell aggregation in 4-1BB costimulated CAR-T cells (Fig. 3G and Fig. S6F). Remarkably, we observed that T cells expressing the three 4-1BB CAR mutants showed significantly less content of Annexin-V^+^ cells and higher counts of viable cells comparable to 19.CD28ζ CAR-T cells (Fig. 3H, I). The expression of p-RIPK1 and p-RIPK3 were also reduced in T cells expressing the three 4-1BB CAR mutants (Fig. S6D), and flow cytometry showed significant decrease of p-MLKL in BBζ ΔC10 CAR-T cells (Fig. S6G, H) indicating reduced necroptosis. Overall, these results indicate that 4-1BB via NF-κB pathway not only causes cell aggregation via ICAM-1, but also causes necroptosis and this effect requires TRAF binding to 4-1BB.

### The NF-κB inhibitor A20 controls cell cluster formation and cell death

**Fig. 4:**
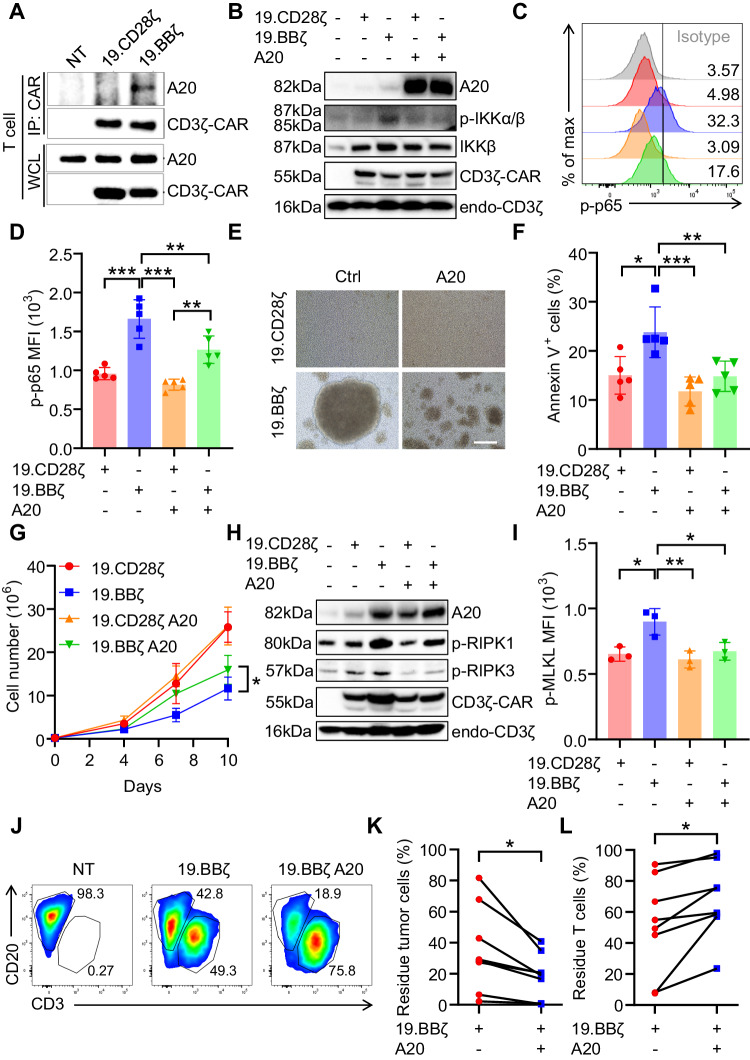
A20 overexpression decreases cell death and improves in vitro functionality of 4-1BB costimulated CAR-T cells. **A** Representative immunoprecipitation blots of CAR-T cells illustrating the binding of A20 to the 19.BBζ CAR in T cells; NT indicates control non-transduced T cells. **B** Representative western blot showing the expression of p-IKKα/β in 19.BBζ CAR-T cells overexpressing A20. The first lane represents NT. **C** Representative flow cytometry histograms showing p-p65 in CAR-T cells with A20 overexpression. Isotype was used as a control, samples legends shown in **D**. **D** Quantification of the mean fluorescence intensity (MFI) showing the level of p-p65 in 19.CD28ζ and 19.BBζ CAR-T cells after A20 overexpression; *n* = 5, **p < 0.01, ***p < 0.001, one-way ANOVA. **E** Representative microscopic imaging showing the formation of cell aggregates of 19.CD28ζ and 19.BBζ CAR-T cells after A20 overexpression. CAR-T cells were analyzed at day 7–10 during the in vitro expansion; magnification 20×; scale bar 100 µm. **F** Quantification of Annexin-V^+^ cells described in (**E**) at day 7–10 of culture; *n* = 5, *p < 0.05, **p < 0.01, ***p < 0.001, one-way ANOVA. **G** Cell counts of the CAR-T cells described in (**E**). Cells were counted with flow cytometry with counting beads; *n* = 4, *p < 0.05 at day 10, two-way ANOVA. **H** Representative western blot testing the expression of p-RIPK1 and p-RIPK3 in 19.BBζ CAR-T cells overexpressing A20. The first line represents NT. **I** Quantification of the p-MLKL MFI in CAR-T cells described in (**E**); *n* = 3, *p < 0.05, **p < 0.01, one-way ANOVA. **J** Representative flow cytometry plots of the experiments in which CAR-T cells were cocultured with Daudi cells for five days (E:T ratio of 1:5). At day five cells were collected to measure Daudi cells (CD20^+^) and T cells (CD3^+^) by flow cytometry. **K**, **L** Summary of the coculture experiments described in (**J**); *n* = 7, *p < 0.05, paired *t*-test

To further investigate how the NF-κB pathway, a key regulator of cell survival [31–34], can be associated with cell death in 4-1BB costimulated CAR-T cells, we focused on the evidence that CoIP-LC-MS/MS showed two downstream negative regulators of the NF-κB signaling - A20 and TNIP1 [35] - almost exclusively identified in the 19.BBζ CAR pull-down. We selected to explore the role of A20 because the function of TNIP1 is A20-dependent [36], A20 mediates RIPK-dependent cell death [32], and A20 contains TRAF-interacting regions [37]. We confirmed the binding of A20 to 19.BBζ CAR via immunoprecipitation (Fig. 4A and Fig. S7A). We also observed that the native 4-1BB in T cells can physiologically interact with A20 in activated T cells (Fig. S7B). To understand the role of A20 binding to the 4-1BB endodomain within the CAR, we performed loss and gain-of-function experiments of A20 in CAR-T cells. We used shRNAs to knock down A20 in both 19.CD28ζ and 19.BBζ CAR-T cells and found that A20 deficiency further caused cell cluster formation and cell death (Fig. S7C–E), and reduced CAR-T cell counts (Fig. S7F). Furthermore, A20 deficiency increased phosphorylation of RIPK1 and RIPK3, which is consistent with an increase in necroptosis (Fig. S7G). In the experiment of gain of function, A20 overexpression decreased NF-κB activity in 19.BBζ CAR-T cells measured as diminished phosphorylation of IKKα/β and p65 (Fig. 4B–D), reduced formation of cell cluster, and reduced ICAM-1 expression (Fig. 4E and Fig. S8A, B). Similar results were observed upon overexpression of A20 in GD2-specific and B7-H3-specific CARs encoding 4-1BB (Fig. S8C–E). In addition, we also found that A20 overexpression in 19.BBζ CAR-T cells reduced the cell death rate (Fig. 4F) and restored numeric CAR-T cell expansion (Fig. 4G). Necroptosis was also reduced as indicated by decreased expression of p-RIPK3/p-MLKL (Fig. 4H, I). A20 overexpression in 19.BBζ CAR-T cells improved the antitumor effects in vitro (Fig. 4J–L). We finally performed bulk RNA sequencing of 19.BBζ CAR-T cells and 19.BBζ CAR-T cells overexpressing A20 after activation with the anti-idiotype Ab crosslinking the CAR.CD19. In 19.BBζ CAR-T cells overexpressing A20 we found increased TNFAIP3 and decreased ICAM-1 and NFKB2 (Fig. S8F), which is consistent with decreased NF-κB pathway (Fig. S8B). We also observed the induction of PIM2, and reduction of BCL2L14 and FAS (Fig. S8F), which are involved in cell survival and proliferation [38–40]. A20 overexpression increased effector signaling molecules like CD28, GZMA, and NKG7. KEGG pathway analysis showed that A20 in addition of regulating the expected necroptosis/apoptosis/NF-κB signaling pathway, regulated pathways involved in inflammatory bowel disease, allograft, and graft-versus-host disease, which are associated with inflammation (Fig. S8G). Finally, since A20 is a deubiquitinating enzyme [41, 42], we observed that A20 overexpression decreased ubiquitination of the 19.BBζ CAR that was previously described [43] (Fig. S9A, B). Overall, these data indicate that the master inhibitor of the NF-κB pathway A20 plays a critical role in controlling both CAR-T cell aggregation and cell death and that A20 seems dysfunctional in 4-1BB costimulated CAR-T cells.

### 4-1BB within the CAR sequesters the NF-κB inhibitor A20 to the cell membrane

**Fig. 5:**
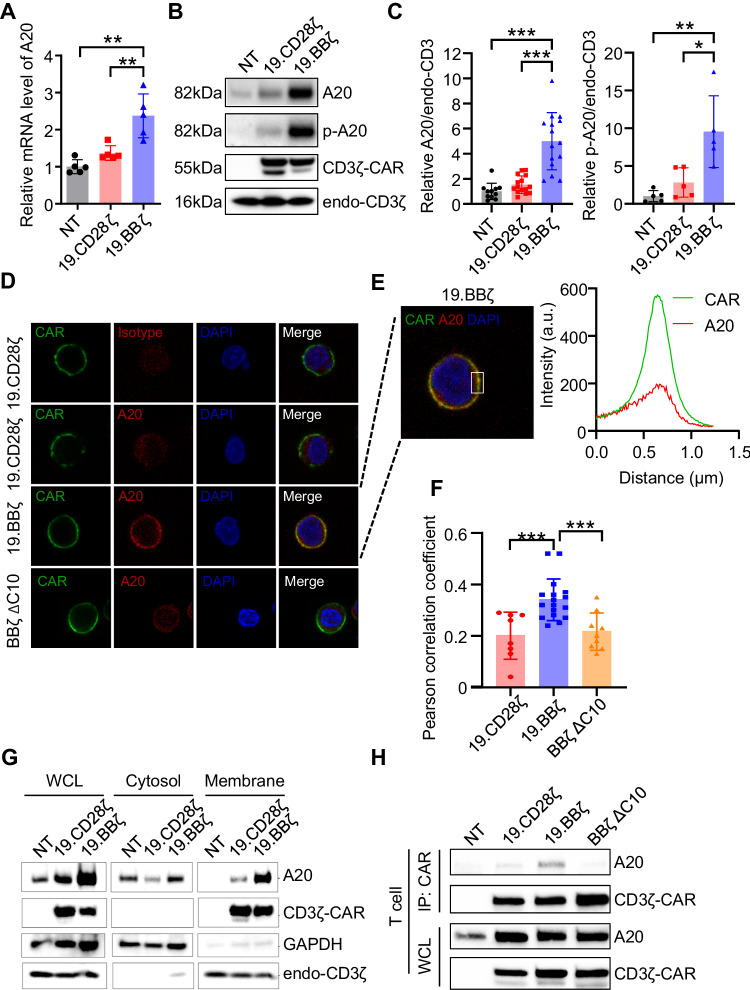
4-1BB endodomain binds A20 within the cell membrane causing A20 functional deficiency in CAR-T cells. **A** mRNA expression of A20 measured by qRT-PCR in NT, 19.CD28ζ, and 19.BBζ CAR-T cells collected at day 7–10 of culture. Data are represented as fold change in expression normalized to the housekeeping gene 18S, and to the expression in NT cells; NT indicates control non-transduced T cells; *n* = 5, **p < 0.01, one-way ANOVA. **B** Representative western blots showing the expression of A20 and its phosphorylated form at Ser^381^ in NT, 19.CD28ζ, and 19.BBζ CAR-T cells collected on day 7 of culture. **C** Quantification of A20 (left panel) and its phosphorylated form at Ser^381^ (right panel) in CAR-T cells illustrated in (**B**); *n* = 11, 15, and 15 for NT, 19.CD28ζ and 19.BBζ CAR-T cells (left panel); *n* = 5 (right panel), *p < 0.05, **p < 0.01, ***p < 0.001, one-way ANOVA. **D** Representative confocal microscopy imaging showing the distribution of A20 in 19.CD28ζ, 19.BBζ, and BBζ ΔC10 CAR-T cells. Blue staining indicates DAPI. Shown are representative cells of a single field; magnification 63×; scale bar 5 µm. Isotype was used as a control. **E** Representative region including the extracellular, membrane, and intracellular areas and the horizontal change of fluorescence intensity of CAR and A20 within the region in 19.BBζ CAR-T cells. **F** Statistics of Pearson correlation coefficients indicating the colocalization of CAR and A20 molecules in 19.CD28ζ, 19.BBζ, and BBζ ΔC10 CAR-T cells; *n* = 8, 17, and 8 for 19.CD28ζ, 19.BBζ, and BBζ ΔC10 CAR-T cells; ***p < 0.001, one-way ANOVA. **G** Representative western blots showing A20 detection in the whole cell lysate (WCL), cytosol, and plasma membrane of NT, 19.CD28ζ, and 19.BBζ CAR-T cells collected at days 7–10 of culture. **H** Representative immunoprecipitation blots of the CD19-specific CAR illustrating the binding of A20 to the 19.BBζ CAR and loss of binding in BBζ ΔC10 CAR-T cells

We performed further analyses to investigate the function of A20 in 4-1BB costimulated CAR-T cells. We analyzed the DEGs dataset [13] and found that A20 was upregulated in both CD19.BBζ and GD2.BBζ CAR-T cells compared to CD28-costimulated CAR-T cells (Fig. S10A). We found that both A20 mRNA (Fig. 5A) and protein (Fig. 5B, C and Fig. S10B, C) were upregulated in 19.BBζ compared to 19.CD28ζ CAR-T cells. Furthermore, 19.BBζ CAR-T cells showed higher A20 phosphorylated at Ser^381^ (Fig. 5B, C), which is a post-translational modification that increases the inhibitory activity of A20 [42]. Elevated levels of A20 were also observed in 4-1BB costimulated CAR-T cells targeting either GD2 or B7-H3 (Fig. S10D, E). Since *A20* is an early NF-κB responsive gene [44], these data indicate that the *A20* transcriptional regulation by NF-κB is conserved in 4-1BB costimulated CAR-T cells. To explore the possibility of a dysfunctional activity of the A20 protein in 4-1BB costimulated CAR-T cells, we studied the A20 cellular localization. Both confocal microscopy (Fig. 5D–F and Fig. S10F, G) and western blot analysis of fractionated cell membrane and cytosol (Fig. 5G) demonstrated that A20 co-localized with the 4-1BB expressing CAR at the plasma membrane. Furthermore, we observed that in CAR-T cells expressing the ΔC10 4-1BB mutant, A20 was released from the cell membrane (Fig. 5D, F, H). Taken together, these results indicate that A20 is upregulated in 4-1BB costimulated CAR-T cells, but A20 binds to 4-1BB within the CAR at the plasma membrane, which compromises its cytoplasmic functional activity in inhibiting NF-κB.

### Abrogating the 4-1BB interaction with A20 improves the antitumor effects of CAR-T cells

**Fig. 6:**
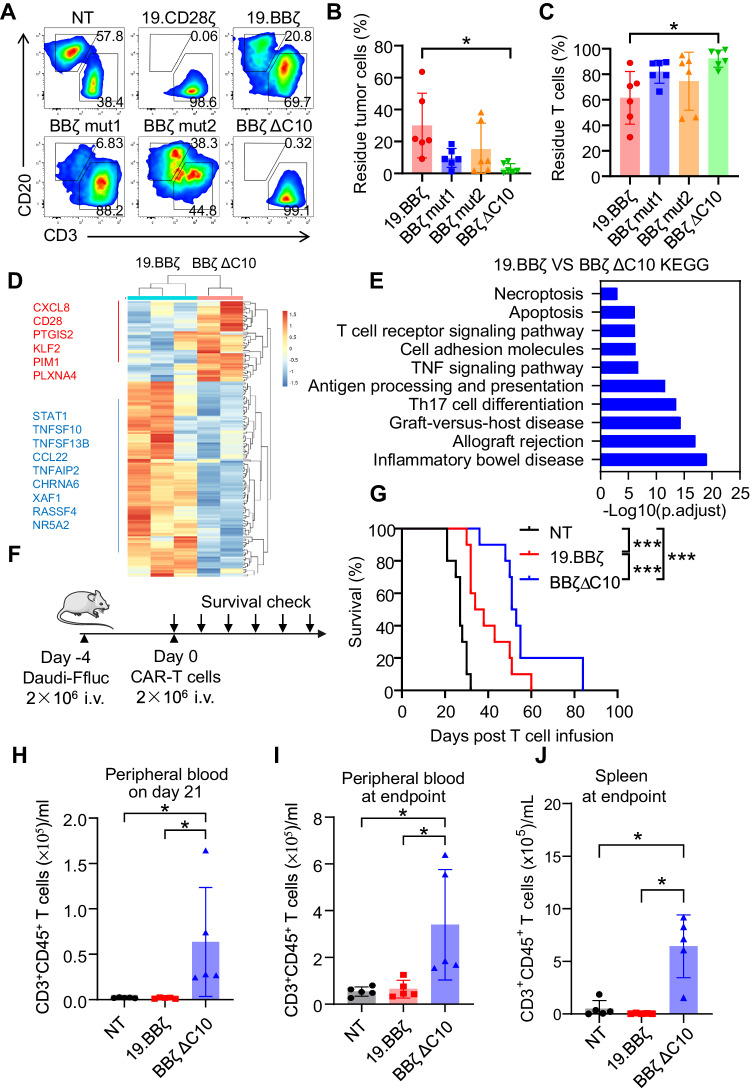
Abrogating the 4-1BB interaction with A20 improves the antitumor effects of CAR-T cells. **A** Representative flow cytometry plots of the experiments in which CAR-T cells were cocultured with Daudi cells for five days (E:T ratio of 1:5). At day five cells were collected to measure Daudi cells (CD20^+^) and T cells (CD3^+^) by flow cytometry. NT indicates control non-transduced T cells. **B**, **C** Summary of the coculture experiments illustrated in (**A**); *n* = 6, *p < 0.05, one-way ANOVA. **D** Heatmap of DEGs comparing 19.BBζ and BBζ ΔC10 CAR-T cells 24 h after activation with the anti-idiotype CAR.CD19 Ab; *n* = 3 for 19.BBζ and *n* = 2 for BBζ ΔC10. **E** KEGG pathway enrichment of DEGs comparing 19.BBζ and BBζ ΔC10 CAR-T cells illustrated in (**D**). **F** Schematic of the Daudi cell model in NSG mice to evaluate the antitumor effects of CAR-T cells in vivo. The i.v. indicates tail vein injection. **G** Kaplan–Meier survival curve of the Daudi tumor model illustrated in (**F**); *n* = 10, ***p < 0.001 by Log-rank test. **H** Numbers of CD3^+^CD45^+^ human T cells detected in the peripheral blood of mice of the Daudi tumor model collected on day 21 post-T-cell infusion; *n* = 5, *p < 0.05, one-way ANOVA. **I**, **J** Numbers of CD3^+^CD45^+^ human T cells in the peripheral blood (**G**) and spleen (**H**) of the mice of the Daudi tumor model at the time of euthanasia from day 25 to day 60 after CAR-T cell infusion; *n* = 5, *p < 0.05, one-way ANOVA

We first used CAR-T cells expressing the 4-1BB mutants (BBζ ΔC10, BBζ mut1, and BBζ mut2) in coculture experiments with CD19^+^CD20^+^ Daudi tumor cells. Only CAR-T cells expressing the BBζ ΔC10 mutant showed improved antitumor effects and enhanced CAR-T cell survival (Fig. 6A–C) without evidence of modification of Granzyme B and cytokine secretion (Fig. S11A–D). We performed RNA sequencing to analyze the molecular signature of 19.BBζ and BBζ ΔC10 CAR-T cells after activation. Compared to 19.BBζ cells, BBζ ΔC10 CAR-T cells showed reduced expression of proapoptotic gene XAF1 [45], RASSF4 [46], and NR5A2 that regulating CD95/Fas ligand (FasL) signaling [47]. BBζ ΔC10 CAR-T cells showed upregulation of KLF2 that enhances effector CD8^+^ T cell differentiation and prevents terminal exhaustion [48], upregulation of CXCL8 involved in T cell effector function [43], PIM1 involved in maintaining CD8^+^ T cell memory quality [49], and PLXNA4 (Plexin-A4) that mediates cytotoxic T cell trafficking [50] (Fig. 6D, E). Next, we evaluated the antitumor effects of CAR-T cells encoding the BBζ ΔC10 mutant in the xenotransplant Daudi model. We found that BBζ ΔC10 CAR-T cells prolonged the survival of tumor-bearing NSG mice compared to 19.BBζ CAR-T cells (Fig. 6F, G). In mice treated with BBζ ΔC10 CAR-T cells, we did not observe superior CAR-T cell tumor infiltration in the spleen and bone marrow at an early time point (Fig. S12A, B), but these mice showed enhanced expansion of CAR-T cells in the peripheral blood at week 3 after infusion, and in the peripheral blood as well as in the spleen at the time of euthanasia (Fig. 6H–J). Overall, these data indicate that abrogating the interaction of A20 with the 4-1BB endodomain improves the antitumor effect of 4-1BB CAR-T cells.

## Discussion

CD28 and 4-1BB endodomains incorporated within the CD19-specific CAR have been FDA-approved for the treatment of B-cell malignancies [1, 2, 51]. However, mechanistic studies characterizing the molecular events occurring when costimulatory endodomains are ectopically engineered within CARs remain limited. Our current study indicates that 4-1BB within the CAR sequesters A20 causing its functional deficiency that in turn leads to NF-κB hyperactivity, cell aggregation via ICAM-1 overexpression, and cell death including necroptosis via RIPK1/RIPK3/MLKL pathway.

CAR tonic signaling caused by self-aggregation of the scFv and leading to premature loss of CAR-T cell function was originally described in CD28-costimulated CAR-T cells, but subsequently demonstrated to be also present in 4-1BB costimulated CAR-T cells [12–15]. It has also been reported that CAR tonic signaling can cause Fas-dependent apoptosis through the continuous activation of NF-κB in 4-1BB costimulation [12]. Here we found that CAR-T cells encoding 4-1BB show pronounced cell cluster formation, cell death, and reduced cell growth without any evidence of CAR tonic signaling [14, 15]. In-depth analysis showed that cell death in CAR-T cells encoding 4-1BB is determined by both apoptosis and necroptosis with the latter specifically identified through the activation of the RIPK3/MLKL pathway as previously demonstrated in murine T cells [52]. Of note, our results are in line with the clinical evidence that commercial CD19.CAR-T product Kymrhian (tisagenlecleucel), which encodes the 4-1BB endodomain, is frequently out of specification since the viability of the product is below 80%, which was established as a requirement from the FDA. Importantly, the observed cell death and cell aggregation in 4-1BB costimulated CAR-T cells are two independent phenomena. Cell death was equally identified in single cells and cells in clusters, and cell aggregates are not formed by death cells, but rather by cell overexpressing the adhesion molecule ICAM-1. However, our data also indicate that the two phenomena of cell cluster formation and cell death are mechanistically unified by the 4-1BB-mediated involvement of the NF-κB/A20 pathway.

NF-κB is recognized for its crucial role in T cell activation following T cell receptor (TCR) engagement and is generally associated with T cell survival [33, 53–55]. 4-1BB costimulation was reported to promote CAR-T cell survival through noncanonical NF-κB signaling upon CAR activation [56]. However, increasing evidence indicates that NF-κB plays more complex roles in balancing cell survival and cell death of immune cells [31, 57, 58]. Our data reveal that 4-1BB costimulated CAR-T cells expanding in vitro in response to cytokines and in the absence of CAR stimulation, have increased canonical NF-κB signaling, but show increased cell death rate and form cell aggregates as compared to CD28-costimulated CAR-T cells. NF-κB pathway is known to promote ICAM-1 expression [25, 59] and thus hyperactive NF-κB explains the pronounced cell cluster formation in 4-1BB costimulated CAR-T cells. Our mechanistic study elucidating the critical role of A20 explains at least in part the unexpected observation of pronounced cell death and necroptosis in CAR-T cells with hyperactive NF-κB pathway.

A20 is a downstream target of NF-κB and acts as a master regulator by tuning inflammation [34, 60]. Specifically in T cells, A20 is constitutively expressed and is reported to remove polyubiquitin chains from MALT1, thereby attenuating T cell receptor (TCR)-mediated NF-κB activity [35, 61]. The A20 ZnF4/7 regions are also required to mediate ubiquitin conjugation to CARD11-BCL10-MALT1 (CBM) complex to downregulate NF-κB signaling in activated T cells [36]. Murine A20-deficient peripheral T cells exhibit necroptosis caused by the ubiquitination of RIPK3 suggesting that A20 is involved in the regulation of necroptosis in T cells [62]. Our data indicate that A20 co-localize with 4-1BB and that A20 is largely sequestered on the cell membrane, which impairs its cytoplasmic inhibitory activity. Our data highlight that both cell aggregation and cell death in 4-1BB costimulated CAR-T cells are corrected by A20 overexpression, while A20 knockdown promotes cell death in CD28-costimulated CAR-T cells, replicating the effects observed in 4-1BB costimulated CAR-T cells. These data parallel the findings in murine A20-deficient peripheral T cells, which exhibit reduced in vitro survival [62]. Additionally, we provide data indicating that A20 release from the cell membrane obtained by 4-1BB mutants preventing the TRAF/A20 binding is also abrogating cell cluster formation and cell death further highlighting the critical role of A20. Of note, we also found that while 4-1BB TRAF mut1 and mut2 prevented cell death, but compromised the antitumor effects of CAR-T cells since TRAF binding to 4-1BB is critical for its signaling [33]. In contrast, the BBζ ΔC10 mutation, which leaves the TRAF2 binding site intact, not only prevents cell death but also improves the antitumor ability of 4-1BB CAR-T cells. We have previously reported that the BBζ ΔC10 mutant prevents the binding of the THEMIS-SHP1 complex to 4-1BB [8]. Here, we observed that BBζ ΔC10 mutation also releases A20 indicating that the last 10 AAs of the COOH-tail region of 4-1BB can exploit multiple functions.

Overall, our study demonstrates that the 4-1BB endodomain in CAR-T cells causes cell aggregation and cell death in the forms of both apoptosis and necroptosis. Averting cell death by interrupting the A20 interplay with 4-1BB via TRAF-binding modulations could be leveraged to improve the antitumor effects of 4-1BB-costimulated CAR-T cells.

## Materials and methods

### Cell lines

293T cells, Jurkat cell line, and Daudi cell line were purchased from ATCC. 293T were cultured with IMDM (Gibco) supplemented with 10% FBS (Sigma), 2 mM GlutaMax (Gibco), penicillin (100 units/mL; Gibco) and streptomycin (100 µg/mL; Gibco). Jurkat cells and Daudi cells were cultured with RPMI-1640 (Gibco) supplemented with 10% FBS (Sigma), 2 mM GlutaMax (Gibco), penicillin (100 units/mL; Gibco) and streptomycin (100 µg/mL; Gibco). All cells were cultured at 37 °C with 5% CO_2_. All the cell lines were validated for the absence of mycoplasma.

### Plasmid construction and production of retroviral supernatants

All retroviral vectors were generated using the SFG backbone. All CARs were generated using the single-chain variable fragment (scFv) from specific antibodies (FMC63 scFv for CD19-specific CAR, 14G2a scFv for GD2-specific CAR, and 376.96 scFv for B7-H3-specific CAR), the CD8α stalk and transmembrane domain, the CD28 or 4-1BB intracellular domains, and CD3ζ intracellular domain (ζ-chain) as previously described [15–17]. GFP-tagged CARs were generated as previously described [14]. The 19.CD28ζ shGFP, 19.BBζ shGFP, 19.CD28ζ shA20, and 19.BBζ shA20 plasmids were constructed by inserting specific shRNAs targeting GFP or A20 into the 19.CD28ζ or 19.BBζ backbone using the U6 promoter sequence. The 19.CD28 CD3ζ and 19.BB CD3ζ mutants were generated by mutating PYAPP to AYAAA within the ζ-chain sequence. The 19.BBζ mut1, mut2, and ΔC10 plasmids contain mutations of 4-1BB that remove either the TRAF2 binding site (BBζ mut1, QEED→QAAA), or the TRAF1/2/3 binding sites (BBζ mut2, PEEEE→PEAAA), or delete the last 10 amino acids of the carboxyl-terminal tail (BBζ ΔC10) [8]. Retroviral supernatants were prepared as previously described [16, 63]. Briefly, 293 T cells were transfected with the three-plasmids-system including two plasmids encoding Peg-Pam-e/gag-pol and the RD114 envelope using the GeneJuice transfection reagent (Novagen). Supernatants were collected and filtered after 48 and 72 h.

### Generation of CAR-T cells

Buffy coats from healthy donors were obtained through the Gulf Coast Regional Blood Center (Houston, TX). Peripheral blood mononuclear cells (PBMCs) were isolated with Lymphoprep density separation (Fresenius Kabi Norge) and activated using plates coated with 1 mg/mL anti-CD3 (Miltenyi Biotechnology) and 1 mg/mL anti-CD28 (BD Biosciences) Abs. Forty-eight hours later, T lymphocytes were transduced with retroviral supernatants using retronectin-coated plates (Takara Bio) and expanded in complete medium including 45% RPMI-1640, 45% Click’s medium (Irvine Scientific), 10% FBS (Hyclone), 2 mM GlutaMAX (Gibco), 100 units/mL penicillin (Gibco) and 100 mg/mL streptomycin (Gibco). Cells were fed with IL-7 (10 ng/mL; PeproTech) and IL-15 (5 ng/mL; PeproTech). CAR-T cells were expanded in vitro for 7–10 days as per clinical manufacturing of CAR-T cells [64, 65]. The formation of CAR-T cell aggregates was observed under randomly selected fields by a bright-field microscope (Olympus), and the size was calculated using Fiji software.

### Flow cytometry

CAR expression in T cells was detected as previously described [16, 17, 66]. Human anti-CD3(#560167), CD19(#340364), CD20(#340941), Annexin-V(#556421), and 7-AAD(#559925), Granzyme B(#561142), IFNγ(#563731) Abs were obtained from BD Biosciences. Human anti-p-p65(#3033), cleaved caspase-8(#9496), cleaved caspase-3(#9602), RIPK3(#10188), and p-MLKL(#91689) Abs were obtained from Cell Signaling Technology and were used to stain cells after fixation and permeabilization (BD Biosciences). Chicken anti-Rabbit IgG conjugated with AF594(#A-21442) was obtained from Invitrogen. Cell Trace Violet (#C34557) was purchased from Thermo Scientific. Human anti-ICAM-1 Ab (#353106) and Zombie Aqua™ (#423102) dye were purchased from BioLegend. Counting beads (Invitrogen) were used to count the cells. Samples were acquired on a Canto II or Fortessa flow cytometer from BD, and data were analyzed using the FlowJo software. Necrostatin-1 (Nec-1) was purchased from Medchemexpress (MCE). DMSO was used to dissolve the inhibitors. CAR-T cells were incubated with DMSO or inhibitors for 24 h, followed by flow cytometry analysis after Annexin-V staining.

### Activation of CAR.CD19-T cells with the anti-idiotype Ab

The activation of CAR-T cells was conducted according to our previous report [8]. Briefly, non-tissue culture-treated 24-well plates were coated with the anti-CAR.CD19 Ab (1 μg/ml, 233-4A) overnight. Plates were washed twice before plating T cells (1 × 10^6^ cells/well). Plates were centrifuged at 1000 g for 5 min, and incubated at 37 °C for 24 h.

### ICAM-1 blocking assay

CAR-T cells were treated with 0.5 mg/ml anti-ICAM-1 Ab (#353102, BioLegend) or PBS control for 4 days. The Ab was added every two days. Cell cluster formation and phenotypic characterization were performed on day 8 of culture.

### Confocal microscopy

T cells expressing the CAR fused with GFP were fixed and permeabilized with the Cytofix/Cytoperm buffer (BD Biosciences), stained with DAPI (Invitrogen), washed with PBS, and mounted on glass-bottom microwell dishes (MatTek corporation). For the colocalization analysis, CAR-T cells without GFP fusion were stained with goat-anti-mouse FITC secondary Ab (BD bioscience), followed by fixation, permeabilization, and staining with AF647-conjugated human anti-A20 Ab (sc-166692 AF647, Santa Cruz Biotechnology) and DAPI. Data acquisition was performed on an LSM700 Zeiss laser scanning confocal microscope (objective lens 63×/1.4 Plan Apo Oil, pixel size 0.07 μm, pinhole size 1 AU) using ZEN software (ZEISS Microscopy). All images were acquired using the same settings. Data analyses including fluorescence intensity and Pearson correlation coefficient were performed with the Fiji software.

### Coculture experiments and ELISAs

CAR expression was examined before coculture experiments to normalize the CAR-T cell numbers. Daudi cells (i.e. target cells, 2 × 10^5^ cells/well) were seeded into 24-well plates and T cells were added at effector to target (E:T) ratio of 1:5. After 24 h, supernatants were collected for examining cytokines IFNγ and IL-2 by enzyme-linked immunosorbent assay (ELISA) following manufacturer’s instructions (R&D Systems). ELISA data were collected and analyzed using the Lumina-200 System. Five days after coculture initiation, cells were collected and stained with Zombie followed by anti-human CD3 and CD20 Abs for flow cytometry analysis. To assess the spontaneous secretion of cytokines, control T cells and CAR-T cells (1 × 10^6^ cells/well) were starved for 1 day in 2 mL complete medium without IL-7 and IL-15. Spontaneous secretion of cytokines including IFNγ and IL-2 was detected in the culture supernatants by ELISA.

### Immunoprecipitation

The immunoprecipitation (IP) of CAR-T cells was conducted as previously described [8]. Total proteins obtained from T cells (at least 5 × 10^7^ cells) were extracted using RIPA lysis buffer (Thermo Scientific) supplemented with protease/phosphatase inhibitors cocktails (Thermo Scientific). Equal amounts of total proteins were used for IP and input (whole cell lysate). IP samples were pre-cleared using rabbit/mouse IgG (Thermo Scientific) with protein G magnetic beads (Bio-Rad). Anti-idiotype CAR.CD19 Ab (1:100), anti-4-1BB (1:50, #34594, Cell Signaling Technology), or anti-A20 (1:100, #5630, Cell Signaling Technology) Abs were incubated with the IP samples for 16 h, and protein G beads were then used for the pull-down by magnetic separation. RIPA buffer was used to wash the IP products at least three times. IP products and input were both dissolved in SDS Laemmli buffer (Bio-Rad) supplemented with β-mercaptoethanol for Western blot analysis.

### Mass spectrometry

The mass spectrometry (MS) analysis was performed as previously described [8]. Briefly, IP samples from control and CAR-expressing Jurkat cells and two human CAR-T cell products were subjected to SDS polyacrylamide gel electrophoresis (SDS-PAGE, Bio-Rad) and stained with Coomassie Brilliant Blue (Bio-Rad). Proteins were reduced, alkylated, and in-gel digested with trypsin overnight at 37 °C. Peptides were extracted, desalted, and dried via vacuum centrifugation. The peptide samples were analyzed by liquid chromatography coupled to tandem mass spectrometry (LC-MS/MS) in 3 separated experiments by Thermo Easy nLC 1000 coupled to a QExactive HF or a Waters nanoAcquity coupled to a Thermo LTQ-Orbitrap Velos. Samples were injected onto a PepMap C18 column (75 μm inner diameter ×25 cm, 2 μm particle size) (Thermo Scientific) and separated over a 120 min gradient where mobile phase A was 0.1% formic acid in water and mobile phase B consisted of 0.1% formic acid in ACN. The LTQ-Orbitrap Velos was operated in data-dependent mode where the 10 most abundant precursors were selected for CID fragmentation (35% CE). The QExactive HF was operated in data-dependent mode where the 15 most intense precursors were selected for subsequent HCD fragmentation (27 NCE). Proteome Discoverer (version 2.1, Thermo Scientific) was used to process all raw data, which was searched against a Uniprot human database using Sequest. Data were filtered by a peptide cutoff of 5% false discovery rate (FDR). Spectral counts were used for protein quantification.

### Western blot

Protein lysates obtained from control and CAR-T cells were resolved on 4–15% SDS-PAGE. Proteins were transferred onto polyvinylidene fluoride membranes (Bio-Rad). Membranes were blocked in 5% non-fat milk (Bio-Rad) or 5% bovine serum albumin (BSA, VWR Life Science) in TBS-T and incubated with primary and secondary Abs. The following Abs were used: anti-CD3ζ (1:1000, sc-1239, Santa Cruz Biotechnology), anti-β-actin (1:2000, sc-47778, Santa Cruz Biotechnology), anti-GAPDH (1:2000, sc-47724, Santa Cruz Biotechnology), anti-A20 (1:1000, #5630, Cell Signaling Technology), anti-p-A20 (Ser^381^, 1:1000, #63523, Cell Signaling Technology), anti-p-IKKα/β (Ser^176/180^, 1:1000, #2697, Cell Signaling Technology), anti-p-p65 (Ser^536^, 1:1000, #3033, Cell Signaling Technology), anti-IKKβ (1:1000, #8943, Cell Signaling Technology), anti-p-RIPK1 (Ser^166^, 1:1000, #65746, Cell Signaling Technology), anti-RIPK1 (1:1000, #3493, Cell Signaling Technology), anti-p-RIPK3 (Ser^227^, 1:1000, Abcam, ab209384), anti-RIPK3 (1:1000, #2283 ProSci), anti-p-MLKL (Ser^358^, 1:1000, #91689, Cell Signaling Technology), anti-MLKL (1:1000, #14993, Cell Signaling Technology), anti-cleaved caspase-8 (Asp^374^, 1:1000, #9496, Cell Signaling Technology), anti-4-1BB (1:1000, #34594, Cell Signaling Technology), and horseradish peroxidase conjugated secondary Abs (goat-anti-mouse and goat-anti-Rabbit both from Thermo Scientific). Membranes were developed with SuperSignal West Femto Maximum Sensitivity Substrate (Thermo Scientific) on a Gel station (Bio-Rad).

### Quantitative RT-PCR

RNA was extracted using RNeasy Kit (QIAGEN), followed by reverse transcription to cDNA using Superscript VILO (Invitrogen). Human A20 primers were as follows: Forward: TCCTCAGGCTTTGTATTTGAGC; Reverse: TGTGTATCGGTGCATGGTTTTA. The PowerUp SYBR Green Master Mix (Applied Biosystems) was applied to perform qRT-PCR using the QuantStudio 6 PCR machine (Applied Biosystems) per the manufacturer’s protocol. The 18S ribosomal RNA was used as a housekeeping gene (GENEWIZ). Data were analyzed using the 2^−^^ΔΔCT^ method.

### NF-κB luciferase reporter assay

The NF-κB luciferase reporter assay was conducted to test the transcriptional activity of NF-κB signaling. Briefly, control and CAR-T cells were transduced with the NlucP/NF-κB-RE/Hygro Vector (Promega) at days 3–5 of culture. After incubation for 72 h, T cells were harvested to record the luciferase reporter activity using the Dual-Luciferase Reporter Assay System (Promega) according to the manufacturer’s protocol.

### CAR-T cell proliferation assay

The anti-idiotype CAR.CD19 Ab (1 μg/ml) was used to pre-coated 24-well plates [31]. Control and CAR-T cells (1 × 10^6^ cells) were starved for 1 day, labeled with CTV, and cultured in 1 mL complete medium without IL-7 and IL-15. CTV signal dilution was measured using flow cytometry on day 1 and day 5 gating on T cells.

### In vitro ubiquitination assay

The ubiquitination assay was performed as previously described [35]. Briefly, CAR-expressing Jurkat cells were transduced with a 6His-Flag ubiquitin construct (6HF-Ub), seeded in 15 cm dishes, and grown until reaching 90% confluence. Cells were then washed and harvested in PBS. Of the cell suspension, 80% was lysed in 6 M Guanidine-HCl-containing buffer, sonicated, and filtered, which was used to pull down endogenous proteins conjugated to the 6HF-Ub on Ni^2+^ - nitrilotriacetic acid (NTA) beads. After that, beads were washed thoroughly, and the precipitated proteins were eluted using imidazole. The remaining 20% of cell suspension was pelleted and solubilized using a detergent-containing buffer supplemented with protease inhibitors to prepare inputs. Both pull-down eluates and inputs were separated by SDS-PAGE and analyzed by Western blot.

### RNA sequence and bioinformatics analysis

CAR-T cells on day 7 of culture were activated using the anti-CD19 anti-idiotype Ab and collected for RNA sequencing after 24 h. CAR-T cells non-stimulated were used as control. Total RNA was isolated using RNeasy Mini kits (QIAGEN) following the manufacturer’s instructions. The quality of total RNA was evaluated using RNA 6000 Nano LabChip (Agilent 2100 Bioanalyzer, Santa Clara, CA). All samples had intact 18S and 28S ribosomal RNA bands with RIN numbers from 8.1 to 10 and RNA 260/280 ratios between 1.9 and 2.0. Raw data were processed with SOAPnuke, Bowtie, and RSEM. The differentially expressed genes (DEGs) were identified using DEseq2 algorithms and visualized with the ggplot2 package in R. Additionally, microarray and RNA sequencing datasets were downloaded from GEO for pathway enrichment. The enrichment of KEGG pathways of DEGs comparing CD28 and 4-1BB costimulated CAR-T cells was conducted using the ClusterProfiler R package. Plots including bar plots, Venn plots, and volcano plots according to the DEGs were visualized by ggplot2 R package. Clustering analysis was performed using the ComplexHeatmap R package.

### In vivo mouse model

Male or female NSG (NOD-scid IL2Rgnull) mice were injected intravenously (i.v.) with CD19^+^ Daudi tumor cells labeled with Firefly luciferase (2 × 10^6^ cells) and treated at day 4 with a suboptimal dose of CAR-T cells (2 × 10^6^). Tumor growth was monitored twice a week. On day 21 or endpoint time, blood or spleen was collected for immunophenotyping to detect CAR-T cells. For testing the T cell infiltration, on day 7, spleen and bone marrow were collected to detect CAR-T cells by immunophenotype. All animal experiments were performed in accordance with UNC Animal Husbandry and Institutional Animal Care and Use Committee (IACUC) guidelines and were approved by UNC IACUC.

### Statistical analysis

Data were summarized as the mean ± SD. The student *t*-test, one-way ANOVA, or two-way ANOVA were used to determine statistical significance between groups. Paired *t*-test was conducted to analyze the coculture assay of A20 overexpression. Multiple comparisons were used to calculate the adjusted *p*-value when appropriate. *p* < 0.05 was considered statistically significant. ns represented no significance. All statistical analyses were performed using GraphPad Prism 8 (GraphPad Software, CA).

### Supplementary information

Supplemental figures

Uncrop western files

## Data Availability

To access the RNA-seq data, visit the website https://www.ncbi.nlm.nih.gov/geo/query/acc.cgi?acc=GSE267821 and use the token “kzqpigamjruvnqj”. All other data are available in the main text or the supplementary materials. All data generated in this study can be obtained from the corresponding authors upon reasonable request.
