# Supplementary material for: 4-1BB-encoding CAR causes cell death via sequestration of the ubiquitin-modifying enzyme A20
Source: Cell Mol Immunol. 2024 Jun 27;21(8):905–17. doi: 10.1038/s41423-024-01198-y (PMC11291893; doi:10.1038/s41423-024-01198-y)
Supplement: Supplementary file 1 — Supplemental figures [file 41423_2024_1198_MOESM1_ESM.pptx]

## Slide 1
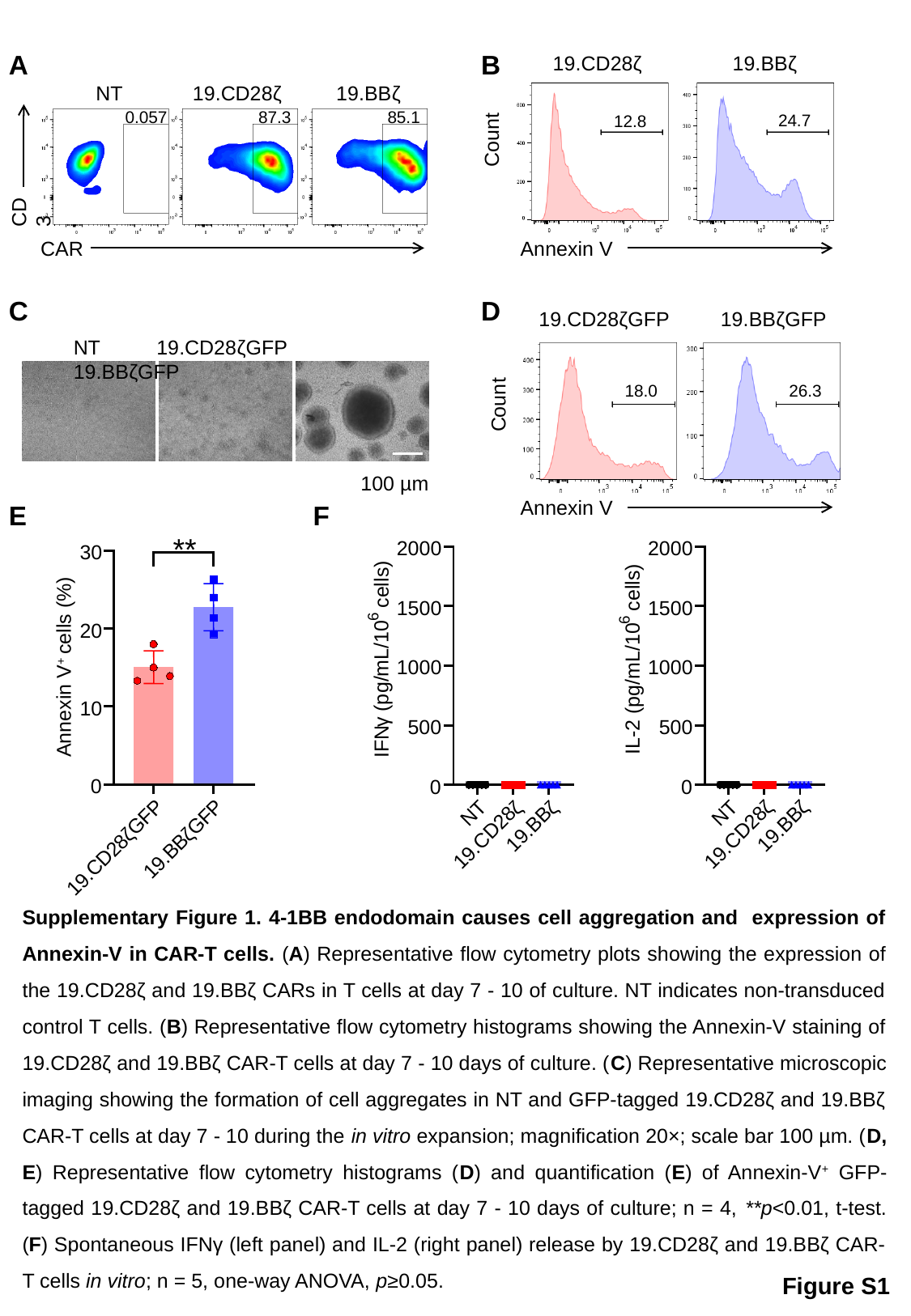

A
B
19.CD28ζ 19.BBζ
Count
24.7
12.8
Annexin V
NT
19.CD28ζ
19.BBζ
0.057
87.3
85.1
CD3
CAR
C
D
19.CD28ζGFP 19.BBζGFP
Count
18.0
26.3
Annexin V
NT 19.CD28ζGFP 19.BBζGFP
100 µm
E
F
2000
 cells)
1500
6
1000
 (pg/mL/10
γ
500
IFN
0
T
ζ
ζ
8
B
N
2
B
D
.
9
C
1
.
9
1
2000
 cells)
1500
6
1000
IL-2 (pg/mL/10
500
0
ζ
ζ
T
8
B
N
2
B
D
.
9
C
1
.
9
1
**
30
20
Annexin V+ cells (%)
10
0
P
P
F
F
G
G
ζ
ζ
8
B
2
B
D
.
9
C
1
.
9
1
Supplementary Figure 1. 4-1BB endodomain causes cell aggregation and expression of Annexin-V in CAR-T cells. (A) Representative flow cytometry plots showing the expression of the 19.CD28ζ and 19.BBζ CARs in T cells at day 7 - 10 of culture. NT indicates non-transduced control T cells. (B) Representative flow cytometry histograms showing the Annexin-V staining of 19.CD28ζ and 19.BBζ CAR-T cells at day 7 - 10 days of culture. (C) Representative microscopic imaging showing the formation of cell aggregates in NT and GFP-tagged 19.CD28ζ and 19.BBζ CAR-T cells at day 7 - 10 during the in vitro expansion; magnification 20×; scale bar 100 µm. (D, E) Representative flow cytometry histograms (D) and quantification (E) of Annexin-V+ GFP-tagged 19.CD28ζ and 19.BBζ CAR-T cells at day 7 - 10 days of culture; n = 4, **p<0.01, t-test. (F) Spontaneous IFNγ (left panel) and IL-2 (right panel) release by 19.CD28ζ and 19.BBζ CAR-T cells in vitro; n = 5, one-way ANOVA, p≥0.05.
Figure S1

## Slide 2
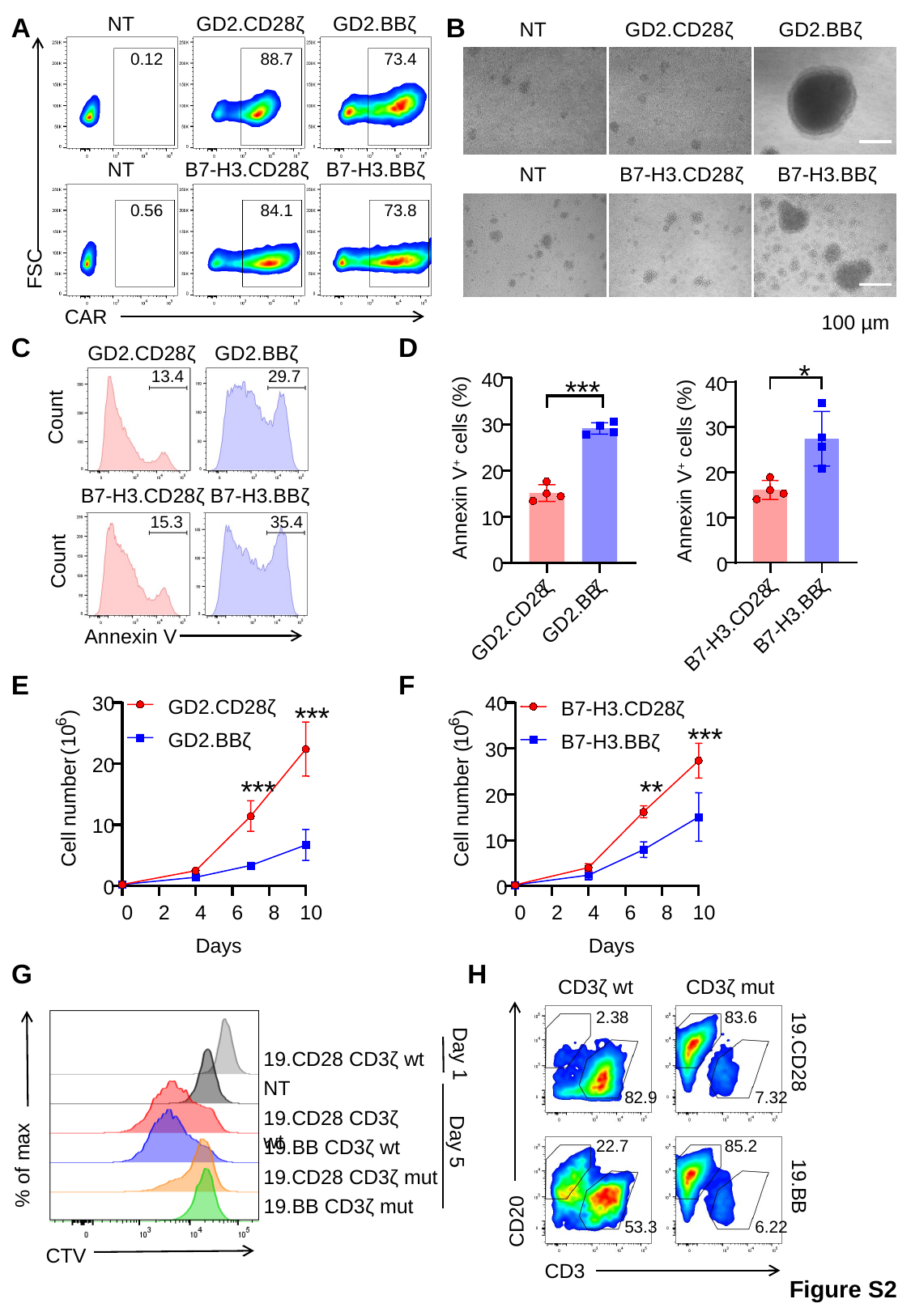

A
B
NT GD2.CD28ζ GD2.BBζ
0.12
88.7
73.4
NT B7-H3.CD28ζ B7-H3.BBζ
0.56
84.1
73.8
FSC
CAR
 NT GD2.CD28ζ GD2.BBζ
 NT B7-H3.CD28ζ B7-H3.BBζ
100 µm
C
D
GD2.CD28ζ 	 GD2.BBζ
13.4
29.7
Count
B7-H3.CD28ζ B7-H3.BBζ
15.3
35.4
Count
Annexin V
*
40
30
Annexin V+ cells (%)
20
10
0
ζ
ζ
8
B
2
B
D
.
3
C
H
.
-
3
7
H
B
-
7
B
40
***
30
Annexin V+ cells (%)
20
10
0
ζ
ζ
8
B
2
B
D
.
2
C
D
.
2
G
D
G
E
F
30
GD2.CD28ζ
***
)
6
10
GD2.BBζ
 (
20
***
Cell number
10
0
0
2
4
6
8
10
Days
40
B7-H3.CD28ζ
)
6
***
10
B7-H3.BBζ
30
**
20
Cell number (
10
0
0
2
4
6
8
10
Days
G
H
CD3ζ wt
CD3ζ mut
2.38
83.6
CD20
CD3
19.CD28
82.9
7.32
22.7
85.2
19.BB
53.3
6.22
% of max
CTV
19.CD28 CD3ζ wt
Day 1
NT
19.CD28 CD3ζ wt
19.BB CD3ζ wt
19.CD28 CD3ζ mut
Day 5
19.BB CD3ζ mut
Figure S2

## Slide 3
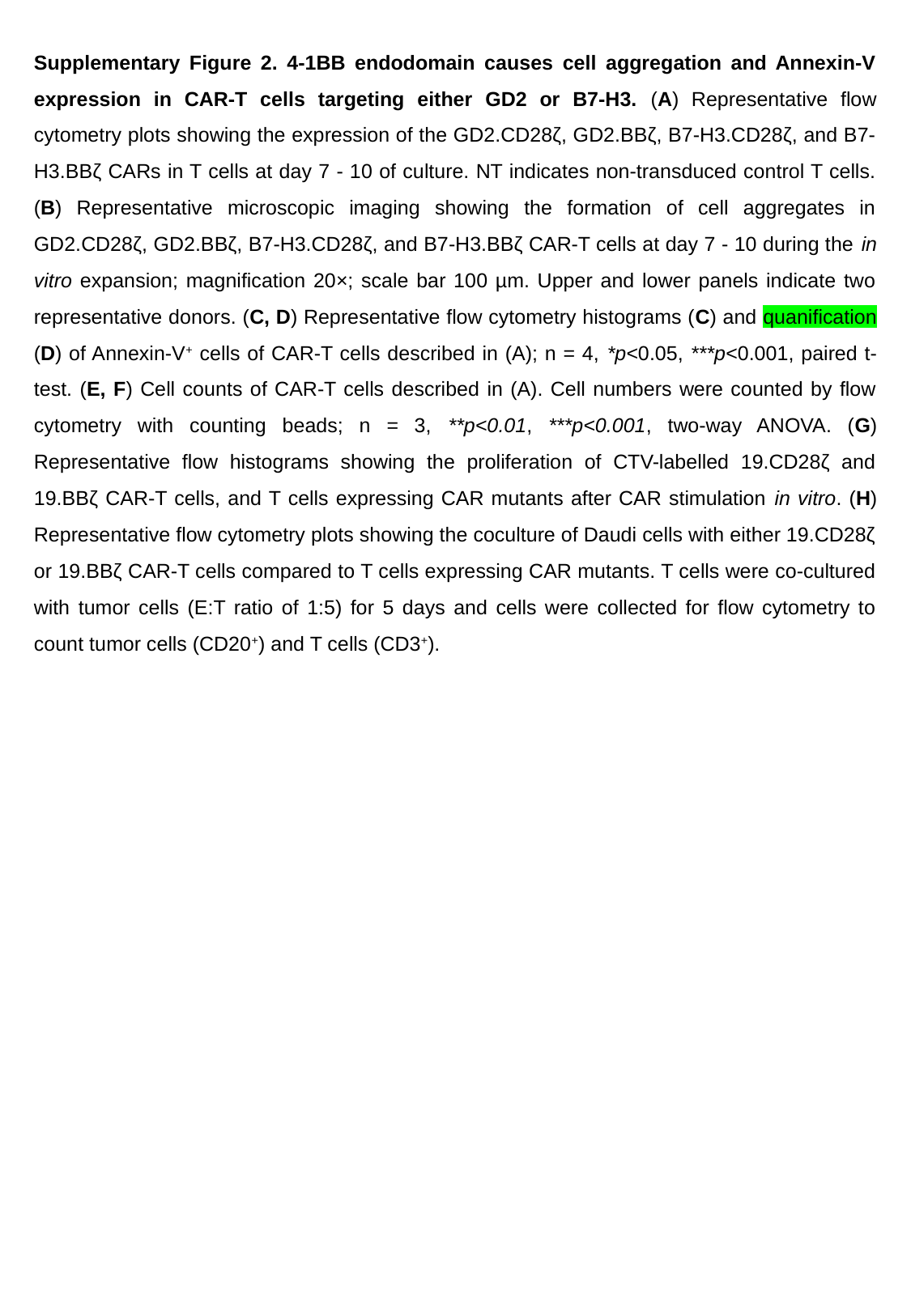

Supplementary Figure 2. 4-1BB endodomain causes cell aggregation and Annexin-V expression in CAR-T cells targeting either GD2 or B7-H3. (A) Representative flow cytometry plots showing the expression of the GD2.CD28ζ, GD2.BBζ, B7-H3.CD28ζ, and B7-H3.BBζ CARs in T cells at day 7 - 10 of culture. NT indicates non-transduced control T cells. (B) Representative microscopic imaging showing the formation of cell aggregates in GD2.CD28ζ, GD2.BBζ, B7-H3.CD28ζ, and B7-H3.BBζ CAR-T cells at day 7 - 10 during the in vitro expansion; magnification 20×; scale bar 100 µm. Upper and lower panels indicate two representative donors. (C, D) Representative flow cytometry histograms (C) and quanification (D) of Annexin-V+ cells of CAR-T cells described in (A); n = 4, *p<0.05, ***p<0.001, paired t-test. (E, F) Cell counts of CAR-T cells described in (A). Cell numbers were counted by flow cytometry with counting beads; n = 3, **p<0.01, ***p<0.001, two-way ANOVA. (G) Representative flow histograms showing the proliferation of CTV-labelled 19.CD28ζ and 19.BBζ CAR-T cells, and T cells expressing CAR mutants after CAR stimulation in vitro. (H) Representative flow cytometry plots showing the coculture of Daudi cells with either 19.CD28ζ or 19.BBζ CAR-T cells compared to T cells expressing CAR mutants. T cells were co-cultured with tumor cells (E:T ratio of 1:5) for 5 days and cells were collected for flow cytometry to count tumor cells (CD20+) and T cells (CD3+).

## Slide 4
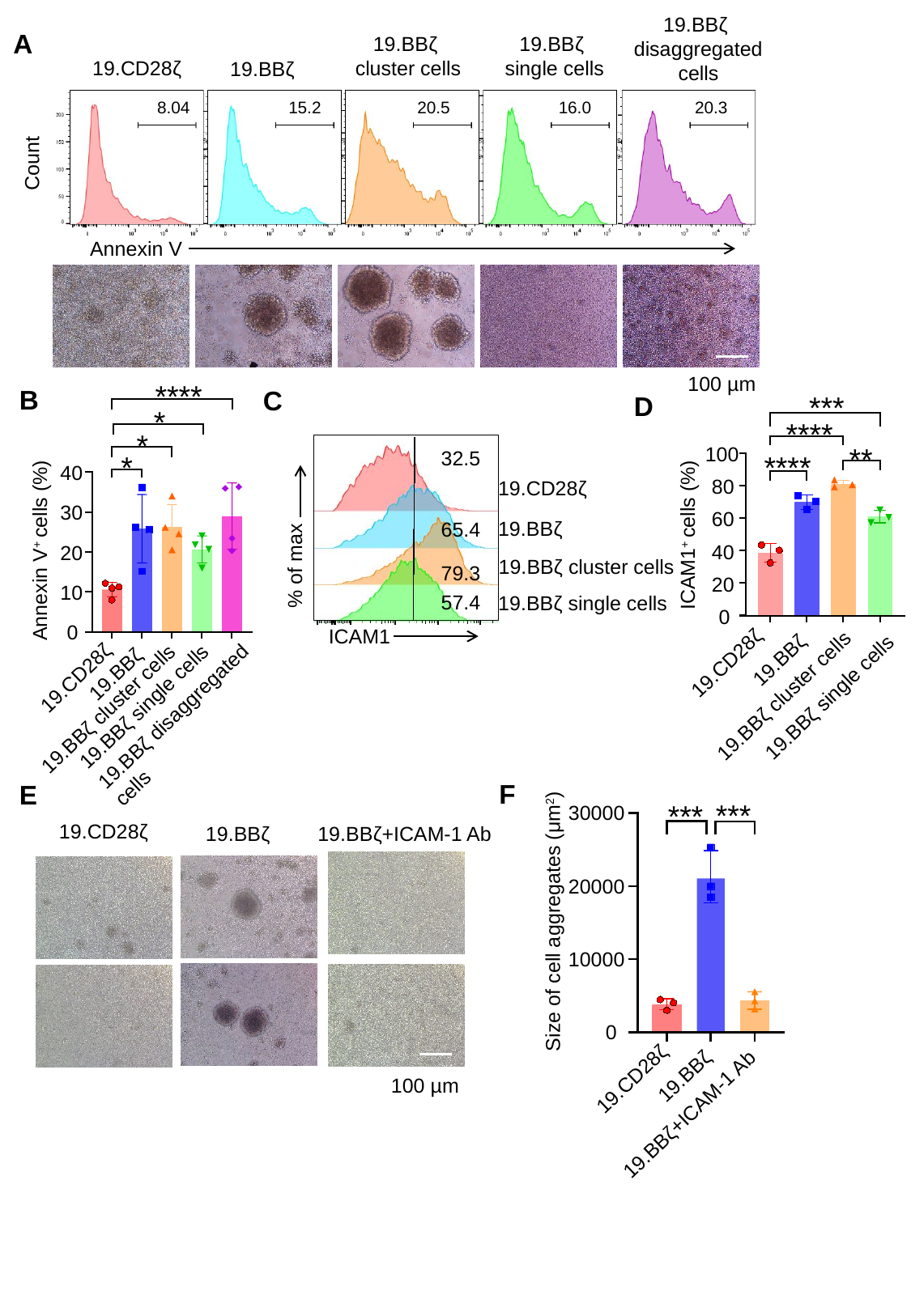

19.BBζ
disaggregated cells
19.BBζ
cluster cells
19.BBζ
single cells
19.CD28ζ
19.BBζ
20.5
8.04
15.2
16.0
20.3
Annexin V
A
Count
100 µm
****
*
*
*
40
30
Annexin V+ cells (%)
20
10
0
19.BBζ
19.CD28ζ
19.BBζ disaggregated cells
19.BBζ single cells
19.BBζ cluster cells
B
C
D
***
****
**
100
****
80
60
ICAM1+ cells (%)
40
20
0
19.BBζ
19.CD28ζ
19.BBζ single cells
19.BBζ cluster cells
32.5
65.4
79.3
57.4
19.CD28ζ
% of max
19.BBζ
19.BBζ cluster cells
19.BBζ single cells
ICAM1
F
E
***
***
30000
20000
Size of cell aggregates (μm2)
10000
0
19.BBζ
19.CD28ζ
19.BBζ+ICAM-1 Ab
19.CD28ζ
19.BBζ
19.BBζ+ICAM-1 Ab
100 µm

## Slide 5
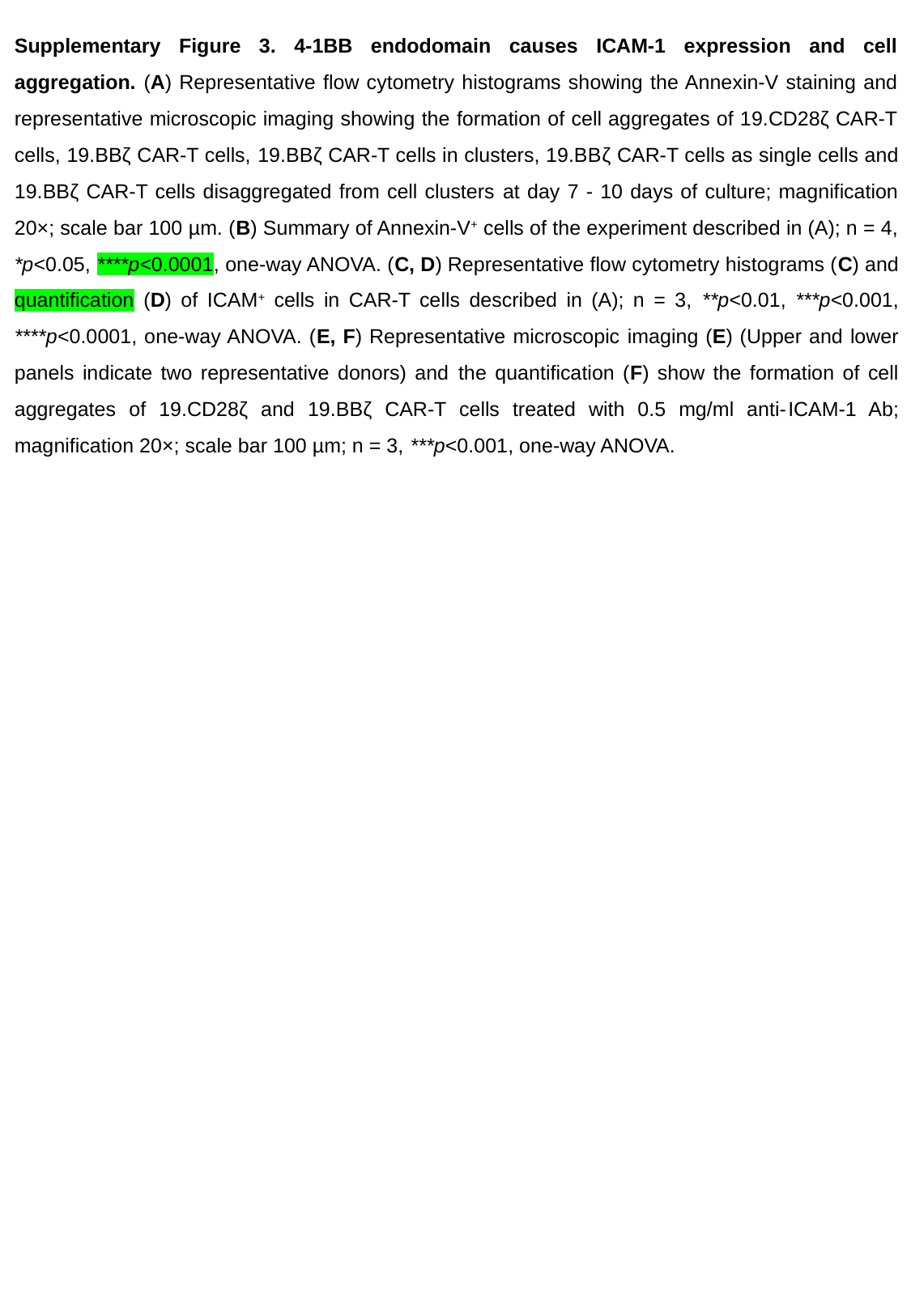

Supplementary Figure 3. 4-1BB endodomain causes ICAM-1 expression and cell aggregation. (A) Representative flow cytometry histograms showing the Annexin-V staining and representative microscopic imaging showing the formation of cell aggregates of 19.CD28ζ CAR-T cells, 19.BBζ CAR-T cells, 19.BBζ CAR-T cells in clusters, 19.BBζ CAR-T cells as single cells and 19.BBζ CAR-T cells disaggregated from cell clusters at day 7 - 10 days of culture; magnification 20×; scale bar 100 µm. (B) Summary of Annexin-V+ cells of the experiment described in (A); n = 4, *p<0.05, ****p<0.0001, one-way ANOVA. (C, D) Representative flow cytometry histograms (C) and quantification (D) of ICAM+ cells in CAR-T cells described in (A); n = 3, **p<0.01, ***p<0.001, ****p<0.0001, one-way ANOVA. (E, F) Representative microscopic imaging (E) (Upper and lower panels indicate two representative donors) and the quantification (F) show the formation of cell aggregates of 19.CD28ζ and 19.BBζ CAR-T cells treated with 0.5 mg/ml anti-ICAM-1 Ab; magnification 20×; scale bar 100 µm; n = 3, ***p<0.001, one-way ANOVA.

## Slide 6
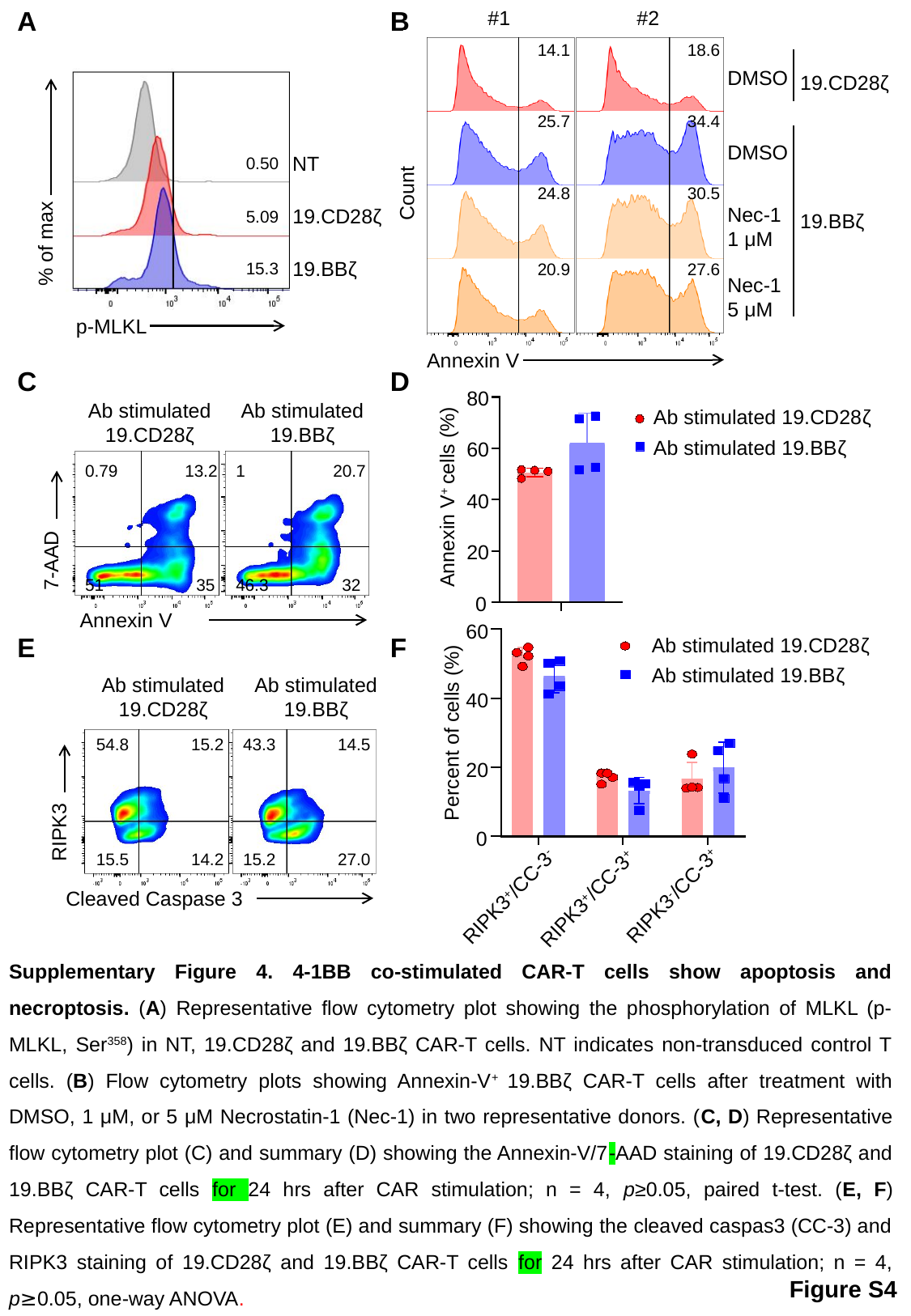

A
B
#1
#2
14.1
18.6
DMSO
19.CD28ζ
25.7
34.4
DMSO
24.8
30.5
Nec-1
1 μM
19.BBζ
20.9
27.6
Nec-1
5 μM
Annexin V
% of max
p-MLKL
NT
0.50
19.CD28ζ
5.09
19.BBζ
15.3
Count
C
D
80
Ab stimulated 19.CD28ζ
Ab stimulated 19.BBζ
60
Annexin V+ cells (%)
40
20
0
Ab stimulated
19.CD28ζ
Ab stimulated
19.BBζ
0.79
13.2
1
20.7
51
35
46.3
32
Annexin V
7-AAD
60
Ab stimulated 19.CD28ζ
Ab stimulated 19.BBζ
40
Percent of cells (%)
20
0
RIPK3-/CC-3+
RIPK3+/CC-3-
RIPK3+/CC-3+
E
F
Ab stimulated
19.CD28ζ
Ab stimulated
19.BBζ
54.8
15.2
43.3
14.5
RIPK3
15.5
14.2
15.2
27.0
Cleaved Caspase 3
Supplementary Figure 4. 4-1BB co-stimulated CAR-T cells show apoptosis and necroptosis. (A) Representative flow cytometry plot showing the phosphorylation of MLKL (p-MLKL, Ser358) in NT, 19.CD28ζ and 19.BBζ CAR-T cells. NT indicates non-transduced control T cells. (B) Flow cytometry plots showing Annexin-V+ 19.BBζ CAR-T cells after treatment with DMSO, 1 μM, or 5 μM Necrostatin-1 (Nec-1) in two representative donors. (C, D) Representative flow cytometry plot (C) and summary (D) showing the Annexin-V/7-AAD staining of 19.CD28ζ and 19.BBζ CAR-T cells for 24 hrs after CAR stimulation; n = 4, p≥0.05, paired t-test. (E, F) Representative flow cytometry plot (E) and summary (F) showing the cleaved caspas3 (CC-3) and RIPK3 staining of 19.CD28ζ and 19.BBζ CAR-T cells for 24 hrs after CAR stimulation; n = 4, p≥0.05, one-way ANOVA.
Figure S4

## Slide 7
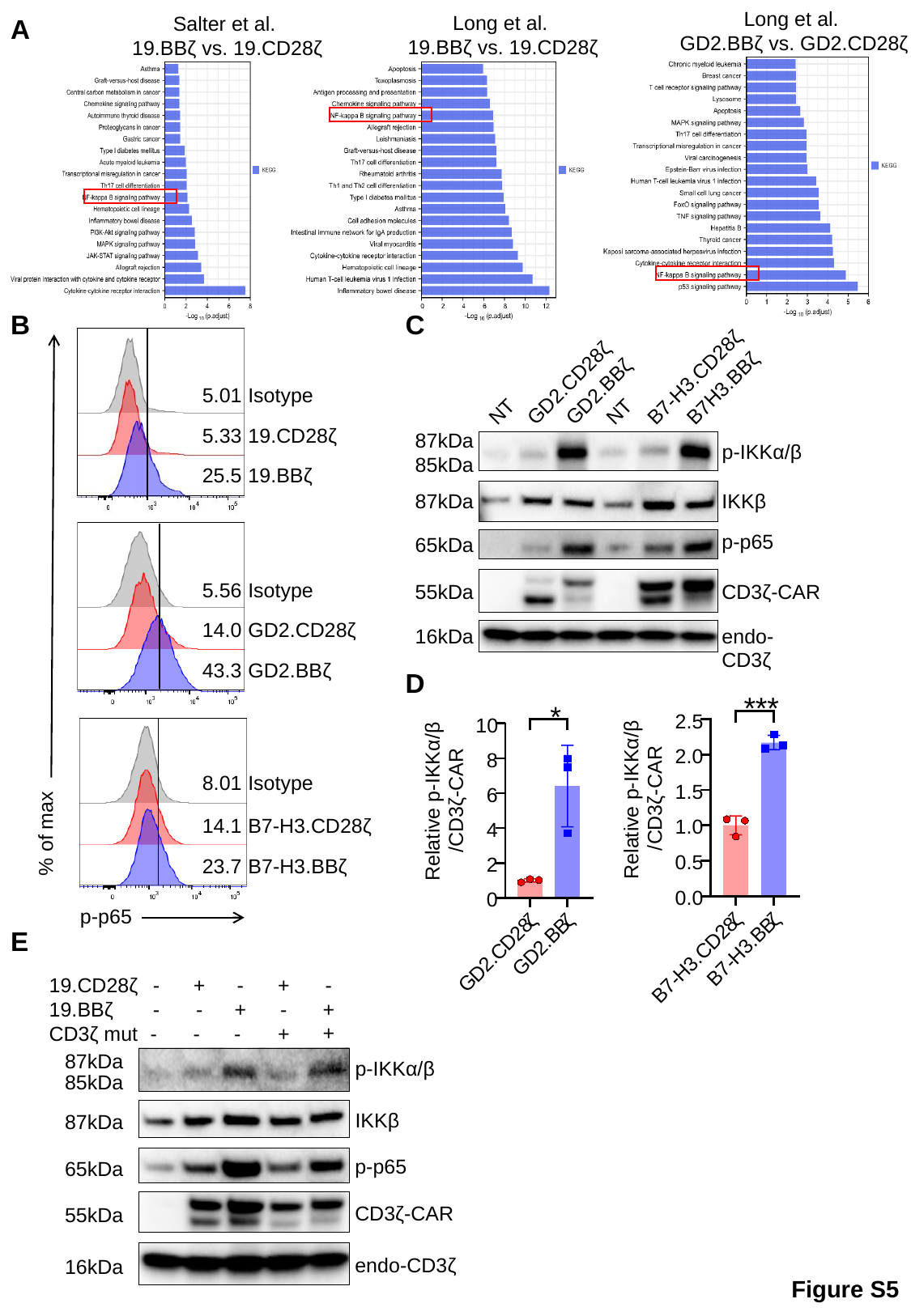

Long et al.
GD2.BBζ vs. GD2.CD28ζ
Long et al.
19.BBζ vs. 19.CD28ζ
Salter et al.
19.BBζ vs. 19.CD28ζ
A
B
C
% of max
p-p65
5.01
Isotype
5.33
19.CD28ζ
25.5
19.BBζ
5.56
Isotype
14.0
GD2.CD28ζ
43.3
GD2.BBζ
8.01
Isotype
14.1
B7-H3.CD28ζ
23.7
B7-H3.BBζ
B7-H3.CD28ζ
GD2.CD28ζ
B7H3.BBζ
GD2.BBζ
NT
NT
87kDa
p-IKKα/β
85kDa
87kDa
IKKβ
p-p65
65kDa
55kDa
CD3ζ-CAR
16kDa
endo-CD3ζ
D
***
2.5
2.0
1.5
/CD3ζ-CAR
Relative p-IKKα/β
1.0
0.5
0.0
ζ
ζ
8
B
2
B
D
.
3
C
H
.
-
3
7
H
B
-
7
B
*
10
8
6
/CD3ζ-CAR
Relative p-IKKα/β
4
2
0
ζ
ζ
8
B
2
B
.
D
2
C
D
.
2
G
D
G
E
19.CD28ζ
19.BBζ
CD3ζ mut
-
-
-
+
-
-
-
+
-
+
-
+
-
+
+
87kDa
p-IKKα/β
85kDa
IKKβ
87kDa
p-p65
65kDa
CD3ζ-CAR
55kDa
endo-CD3ζ
16kDa
Figure S5

## Slide 8
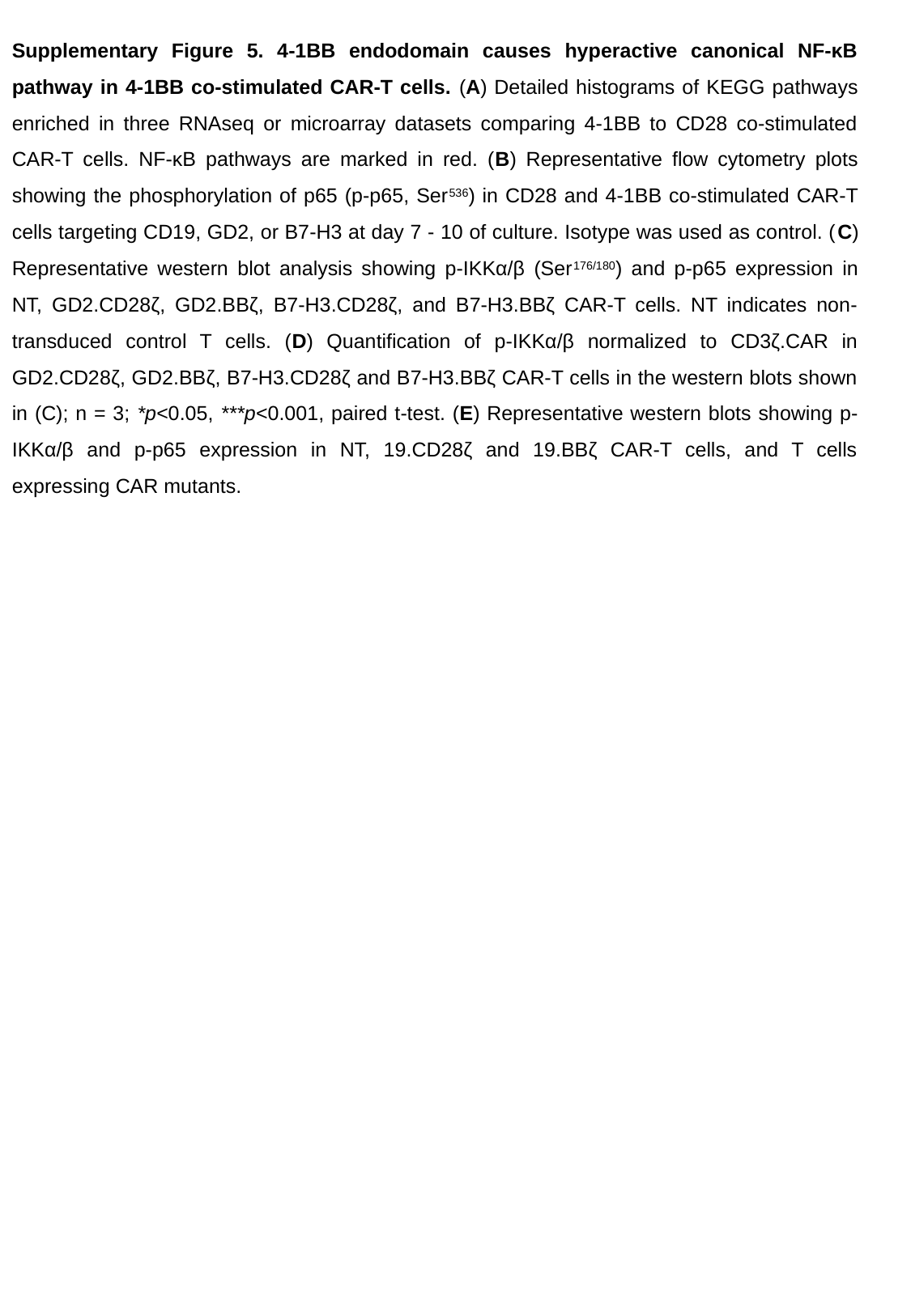

Supplementary Figure 5. 4-1BB endodomain causes hyperactive canonical NF-κB pathway in 4-1BB co-stimulated CAR-T cells. (A) Detailed histograms of KEGG pathways enriched in three RNAseq or microarray datasets comparing 4-1BB to CD28 co-stimulated CAR-T cells. NF-κB pathways are marked in red. (B) Representative flow cytometry plots showing the phosphorylation of p65 (p-p65, Ser536) in CD28 and 4-1BB co-stimulated CAR-T cells targeting CD19, GD2, or B7-H3 at day 7 - 10 of culture. Isotype was used as control. (C) Representative western blot analysis showing p-IKKα/β (Ser176/180) and p-p65 expression in NT, GD2.CD28ζ, GD2.BBζ, B7-H3.CD28ζ, and B7-H3.BBζ CAR-T cells. NT indicates non-transduced control T cells. (D) Quantification of p-IKKα/β normalized to CD3ζ.CAR in GD2.CD28ζ, GD2.BBζ, B7-H3.CD28ζ and B7-H3.BBζ CAR-T cells in the western blots shown in (C); n = 3; *p<0.05, ***p<0.001, paired t-test. (E) Representative western blots showing p-IKKα/β and p-p65 expression in NT, 19.CD28ζ and 19.BBζ CAR-T cells, and T cells expressing CAR mutants.

## Slide 9
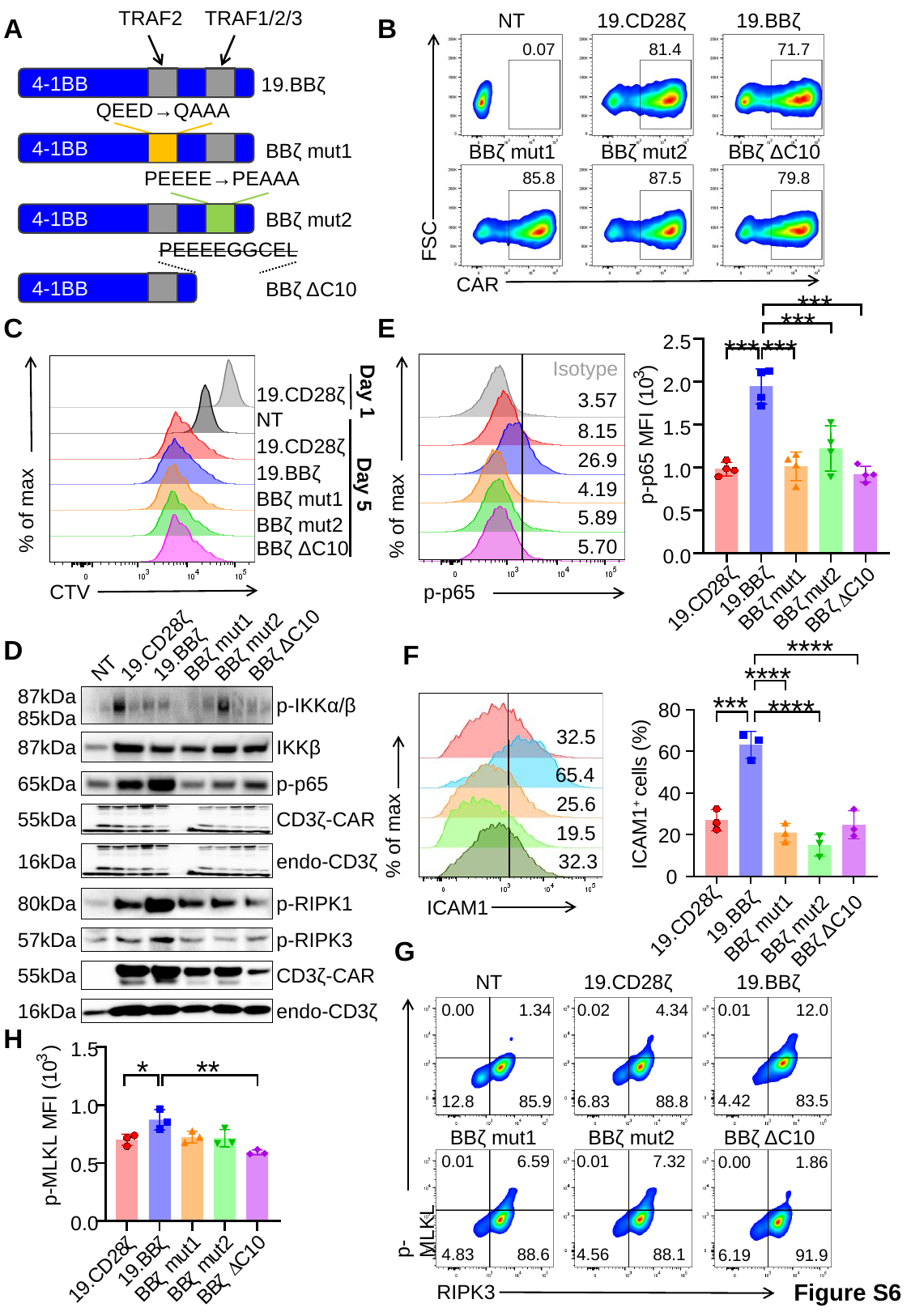

TRAF2 TRAF1/2/3
19.BBζ
4-1BB
QEED→QAAA
BBζ mut1
4-1BB
PEEEE→PEAAA
BBζ mut2
4-1BB
PEEEEGGCEL
BBζ ΔC10
4-1BB
NT
19.CD28ζ
19.BBζ
0.07
81.4
71.7
BBζ mut1
BBζ mut2
BBζ ΔC10
85.8
87.5
79.8
FSC
CAR
A
B
***
***
2.5
***
***
)
3
2.0
1.5
p-p65 MFI (10
1.0
0.5
0.0
ζ
ζ
0
1
2
t
t
8
B
1
u
u
2
B
C
m
m
D
.
D
9
ζ
ζ
C
ζ
1
.
B
B
B
9
B
B
1
B
C
E
Isotype
% of max
p-p65
3.57
8.15
26.9
4.19
5.89
5.70
% of max
CTV
19.CD28ζ
Day 1
NT
19.CD28ζ
19.BBζ
BBζ mut1
Day 5
BBζ mut2
BBζ ΔC10
BBζ ΔC10
19.CD28ζ
BBζ mut2
BBζ mut1
19.BBζ
NT
87kDa
p-IKKα/β
85kDa
87kDa
IKKβ
65kDa
p-p65
55kDa
CD3ζ-CAR
16kDa
endo-CD3ζ
80kDa
p-RIPK1
57kDa
p-RIPK3
55kDa
CD3ζ-CAR
16kDa
endo-CD3ζ
D
****
****
***
****
80
60
40
ICAM1+ cells (%)
20
0
19.BBζ
19.CD28ζ
BBζ mut1
BBζ mut2
BBζ ΔC10
F
32.5
% of max
25.6
32.3
ICAM1
65.4
19.5
G
NT
19.CD28ζ
19.BBζ
0.01
12.0
0.00
1.34
0.02
4.34
4.42
83.5
12.8
85.9
6.83
88.8
BBζ mut1
BBζ mut2
BBζ ΔC10
0.01
6.59
0.01
7.32
0.00
1.86
p-MLKL
4.83
88.6
4.56
88.1
6.19
91.9
RIPK3
)
1.5
3
*
**
1.0
p-MLKL MFI (10
0.5
0.0
0
1
2
ζ
ζ
t
t
8
B
1
u
u
2
B
C
m
m
D
.
D
9
C
1
ζ
ζ
.
ζ
B
B
B
9
B
B
1
B
H
Figure S6

## Slide 10
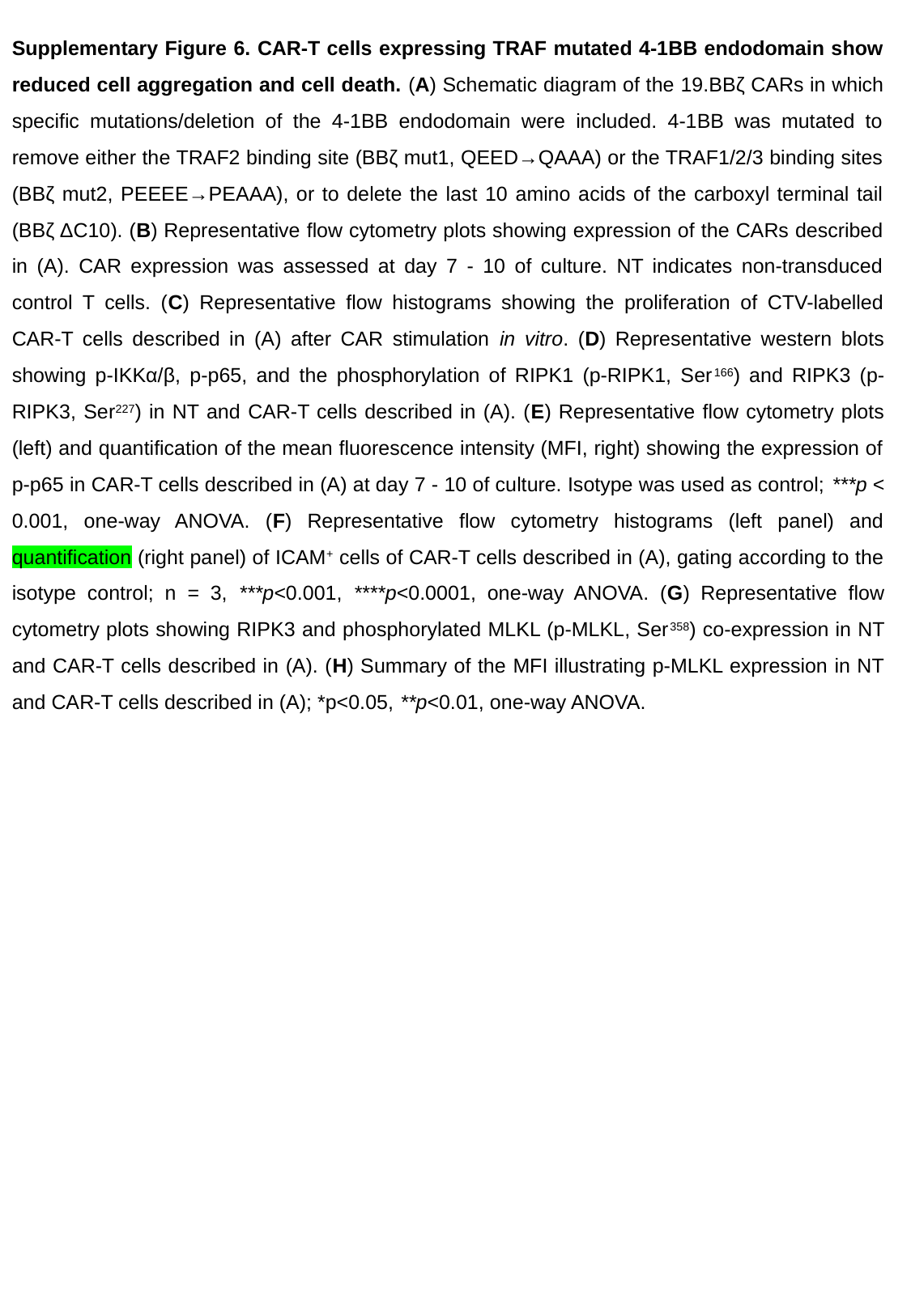

Supplementary Figure 6. CAR-T cells expressing TRAF mutated 4-1BB endodomain show reduced cell aggregation and cell death. (A) Schematic diagram of the 19.BBζ CARs in which specific mutations/deletion of the 4-1BB endodomain were included. 4-1BB was mutated to remove either the TRAF2 binding site (BBζ mut1, QEED→QAAA) or the TRAF1/2/3 binding sites (BBζ mut2, PEEEE→PEAAA), or to delete the last 10 amino acids of the carboxyl terminal tail (BBζ ΔC10). (B) Representative flow cytometry plots showing expression of the CARs described in (A). CAR expression was assessed at day 7 - 10 of culture. NT indicates non-transduced control T cells. (C) Representative flow histograms showing the proliferation of CTV-labelled CAR-T cells described in (A) after CAR stimulation in vitro. (D) Representative western blots showing p-IKKα/β, p-p65, and the phosphorylation of RIPK1 (p-RIPK1, Ser166) and RIPK3 (p-RIPK3, Ser227) in NT and CAR-T cells described in (A). (E) Representative flow cytometry plots (left) and quantification of the mean fluorescence intensity (MFI, right) showing the expression of p-p65 in CAR-T cells described in (A) at day 7 - 10 of culture. Isotype was used as control; ***p < 0.001, one-way ANOVA. (F) Representative flow cytometry histograms (left panel) and quantification (right panel) of ICAM+ cells of CAR-T cells described in (A), gating according to the isotype control; n = 3, ***p<0.001, ****p<0.0001, one-way ANOVA. (G) Representative flow cytometry plots showing RIPK3 and phosphorylated MLKL (p-MLKL, Ser358) co-expression in NT and CAR-T cells described in (A). (H) Summary of the MFI illustrating p-MLKL expression in NT and CAR-T cells described in (A); *p<0.05, **p<0.01, one-way ANOVA.

## Slide 11
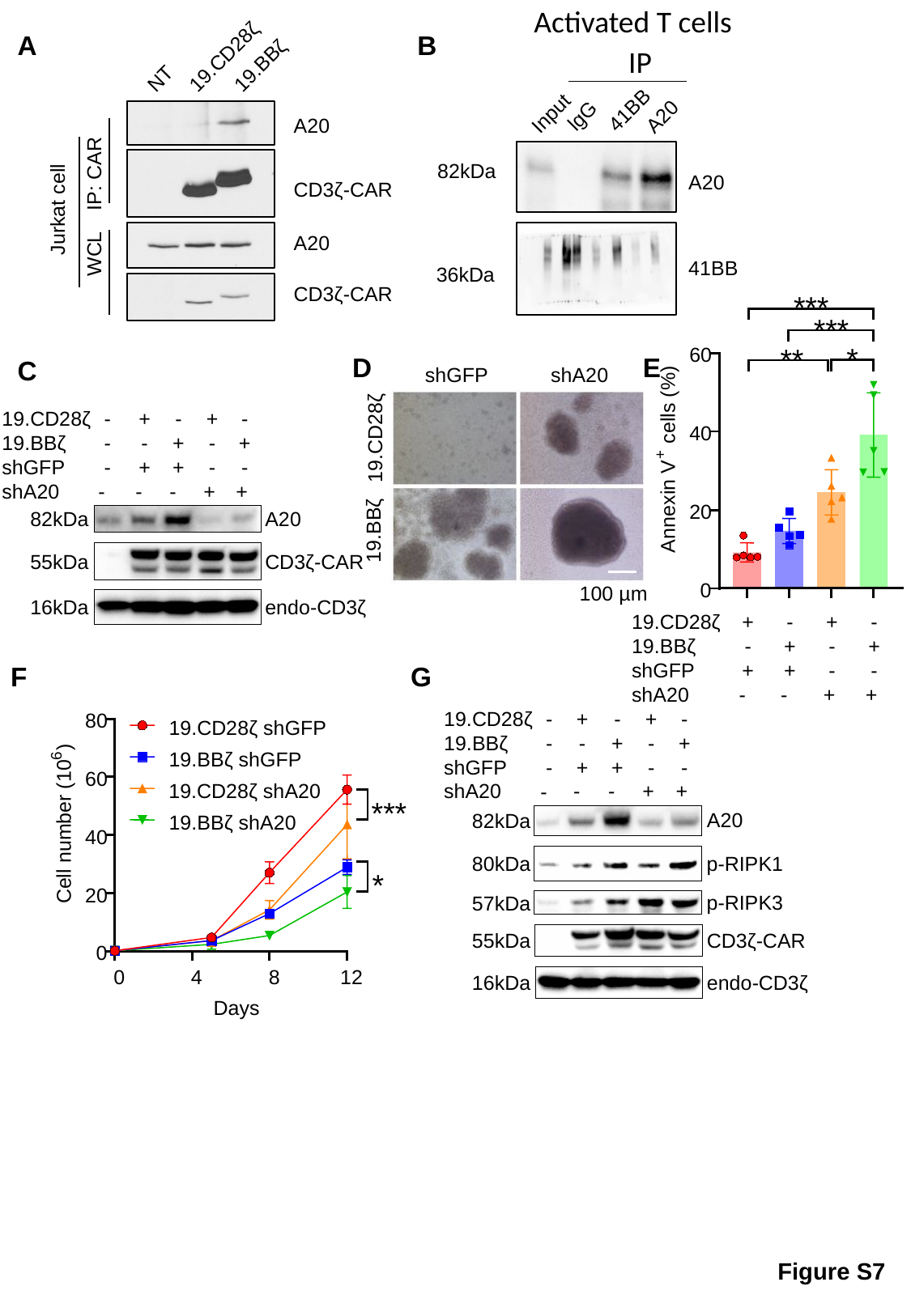

Activated T cells
IP
lgG
Input
41BB
A20
82kDa
A20
41BB
36kDa
A
B
19.CD28ζ
19.BBζ
NT
A20
IP: CAR
CD3ζ-CAR
Jurkat cell
WCL
A20
CD3ζ-CAR
***
***
*
**
60
 cells (%)
40
+
Annexin V
20
0
19.CD28ζ
19.BBζ
shGFP
shA20
+
-
+
-
-
+
+
-
+
-
-
+
-
+
-
+
shA20
shGFP
19.CD28ζ
19.BBζ
E
D
C
19.CD28ζ
19.BBζ
shGFP
shA20
+
-
+
-
-
+
+
-
+
-
-
+
-
+
-
+
-
-
-
-
A20
82kDa
CD3ζ-CAR
55kDa
endo-CD3ζ
16kDa
100 µm
F
G
80
19.CD28ζ shGFP
)
6
19.BBζ shGFP
10
60
19.CD28ζ shA20
***
19.BBζ shA20
40
Cell number (
*
20
0
0
4
8
12
Days
19.CD28ζ
19.BBζ
shGFP
shA20
+
-
+
-
-
+
+
-
+
-
-
+
-
+
-
+
-
-
-
-
A20
82kDa
80kDa
p-RIPK1
p-RIPK3
57kDa
CD3ζ-CAR
55kDa
endo-CD3ζ
16kDa
Figure S7

## Slide 12
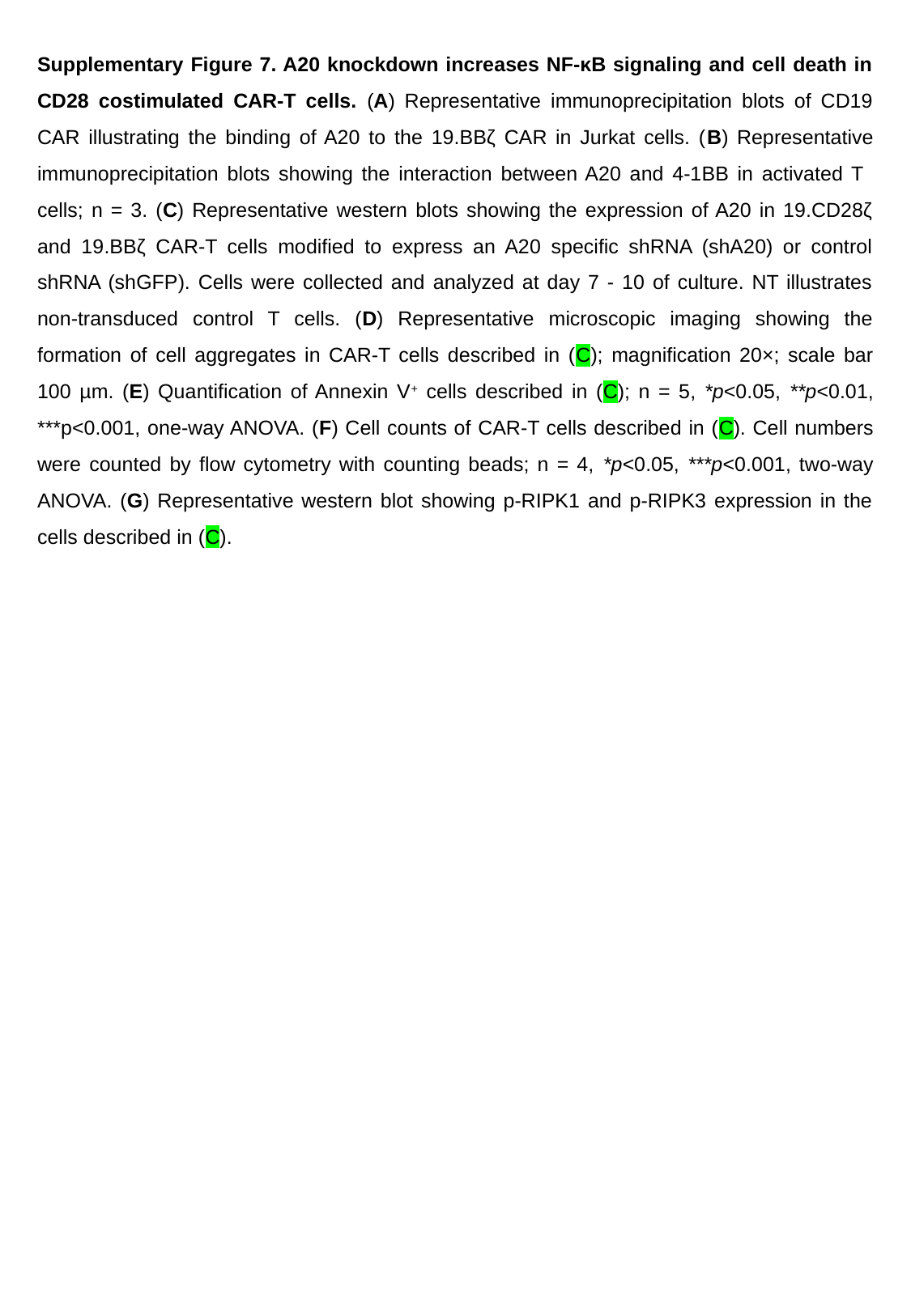

Supplementary Figure 7. A20 knockdown increases NF-κB signaling and cell death in CD28 costimulated CAR-T cells. (A) Representative immunoprecipitation blots of CD19 CAR illustrating the binding of A20 to the 19.BBζ CAR in Jurkat cells. (B) Representative immunoprecipitation blots showing the interaction between A20 and 4-1BB in activated T cells; n = 3. (C) Representative western blots showing the expression of A20 in 19.CD28ζ and 19.BBζ CAR-T cells modified to express an A20 specific shRNA (shA20) or control shRNA (shGFP). Cells were collected and analyzed at day 7 - 10 of culture. NT illustrates non-transduced control T cells. (D) Representative microscopic imaging showing the formation of cell aggregates in CAR-T cells described in (C); magnification 20×; scale bar 100 µm. (E) Quantification of Annexin V+ cells described in (C); n = 5, *p<0.05, **p<0.01, ***p<0.001, one-way ANOVA. (F) Cell counts of CAR-T cells described in (C). Cell numbers were counted by flow cytometry with counting beads; n = 4, *p<0.05, ***p<0.001, two-way ANOVA. (G) Representative western blot showing p-RIPK1 and p-RIPK3 expression in the cells described in (C).

## Slide 13
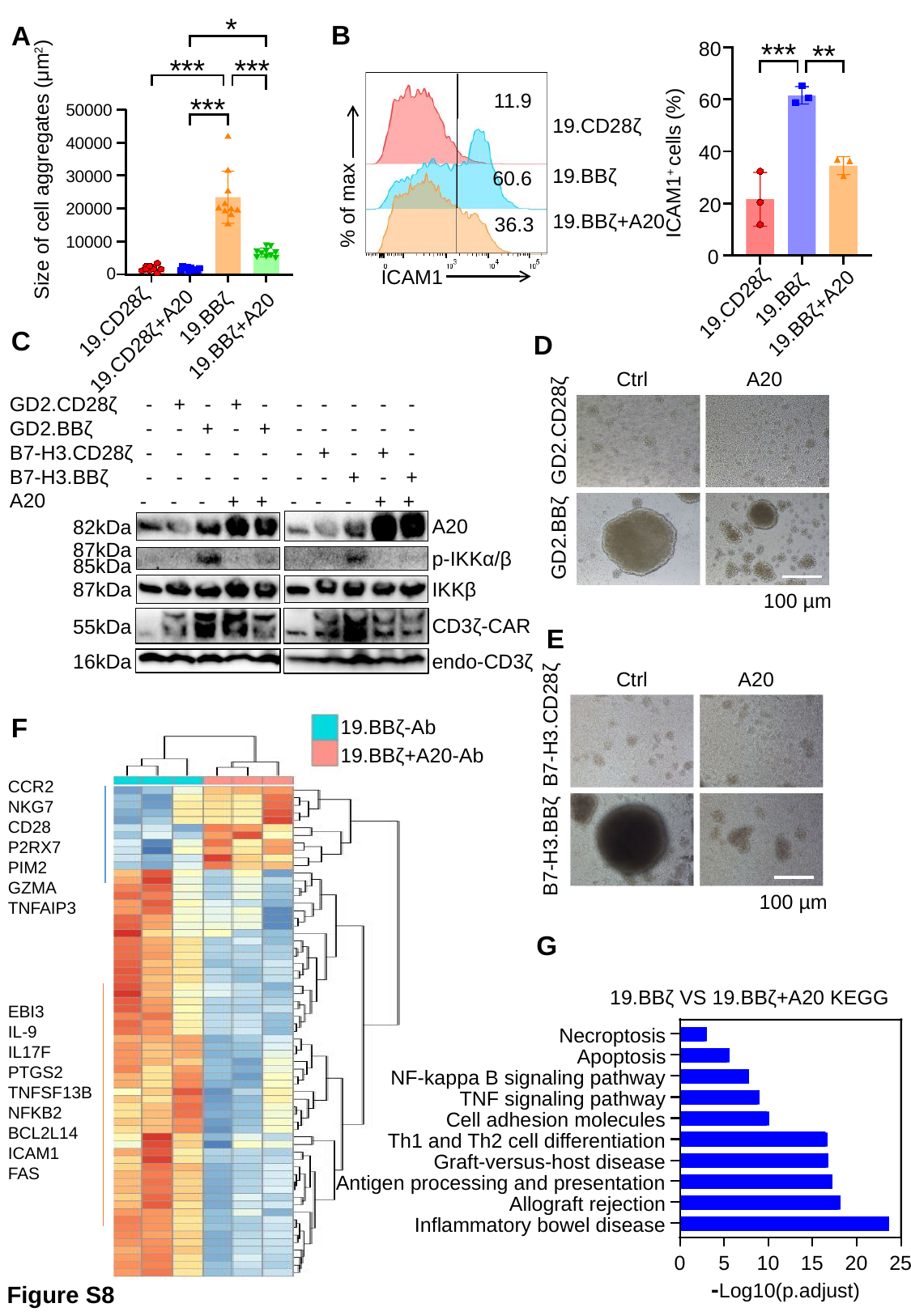

*
***
***
***
50000
40000
Size of cell aggregates (μm2)
30000
20000
10000
0
19.BBζ
19.CD28ζ
19.BBζ+A20
19.CD28ζ+A20
B
A
***
80
**
60
40
ICAM1+ cells (%)
20
0
19.BBζ
19.CD28ζ
19.BBζ+A20
11.9
19.CD28ζ
% of max
19.BBζ
60.6
19.BBζ+A20
36.3
ICAM1
C
D
Ctrl
A20
GD2.CD28ζ
GD2.BBζ
GD2.CD28ζ
GD2.BBζ
B7-H3.CD28ζ
B7-H3.BBζ
A20
-
-
-
-
-
+
-
-
-
-
-
+
-
-
-
+
-
-
-
+
-
+
-
-
+
-
-
-
-
-
-
-
+
-
-
-
-
-
+
-
-
-
+
-
+
-
-
-
+
+
A20
82kDa
87kDa
85kDa
p-IKKα/β
IKKβ
87kDa
CD3ζ-CAR
55kDa
endo-CD3ζ
16kDa
100 µm
E
Ctrl
A20
B7-H3.CD28ζ
B7-H3.BBζ
F
19.BBζ-Ab
19.BBζ+A20-Ab
CCR2
NKG7
CD28
P2RX7
PIM2
GZMA
TNFAIP3
EBI3
IL-9
IL17F
PTGS2
TNFSF13B
NFKB2
BCL2L14
ICAM1
FAS
100 µm
G
19.BBζ VS 19.BBζ+A20 KEGG
Necroptosis
Apoptosis
NF-kappa B signaling pathway
TNF signaling pathway
Cell adhesion molecules
Th1 and Th2 cell differentiation
Graft-versus-host disease
Antigen processing and presentation
Allograft rejection
Inflammatory bowel disease
0
5
10
15
20
25
-Log10(p.adjust)
Figure S8

## Slide 14
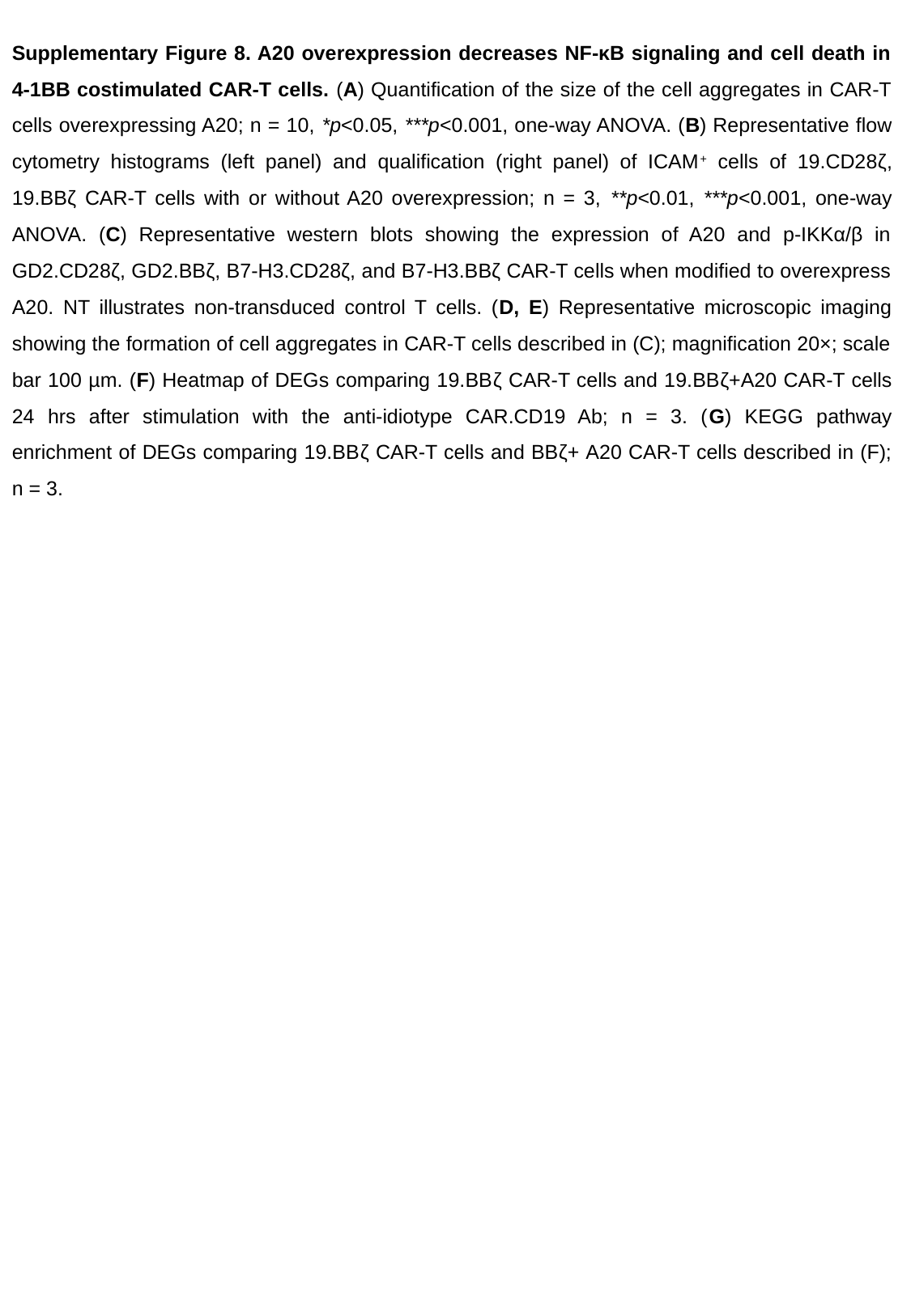

Supplementary Figure 8. A20 overexpression decreases NF-κB signaling and cell death in 4-1BB costimulated CAR-T cells. (A) Quantification of the size of the cell aggregates in CAR-T cells overexpressing A20; n = 10, *p<0.05, ***p<0.001, one-way ANOVA. (B) Representative flow cytometry histograms (left panel) and qualification (right panel) of ICAM+ cells of 19.CD28ζ, 19.BBζ CAR-T cells with or without A20 overexpression; n = 3, **p<0.01, ***p<0.001, one-way ANOVA. (C) Representative western blots showing the expression of A20 and p-IKKα/β in GD2.CD28ζ, GD2.BBζ, B7-H3.CD28ζ, and B7-H3.BBζ CAR-T cells when modified to overexpress A20. NT illustrates non-transduced control T cells. (D, E) Representative microscopic imaging showing the formation of cell aggregates in CAR-T cells described in (C); magnification 20×; scale bar 100 µm. (F) Heatmap of DEGs comparing 19.BBζ CAR-T cells and 19.BBζ+A20 CAR-T cells 24 hrs after stimulation with the anti-idiotype CAR.CD19 Ab; n = 3. (G) KEGG pathway enrichment of DEGs comparing 19.BBζ CAR-T cells and BBζ+ A20 CAR-T cells described in (F); n = 3.

## Slide 15
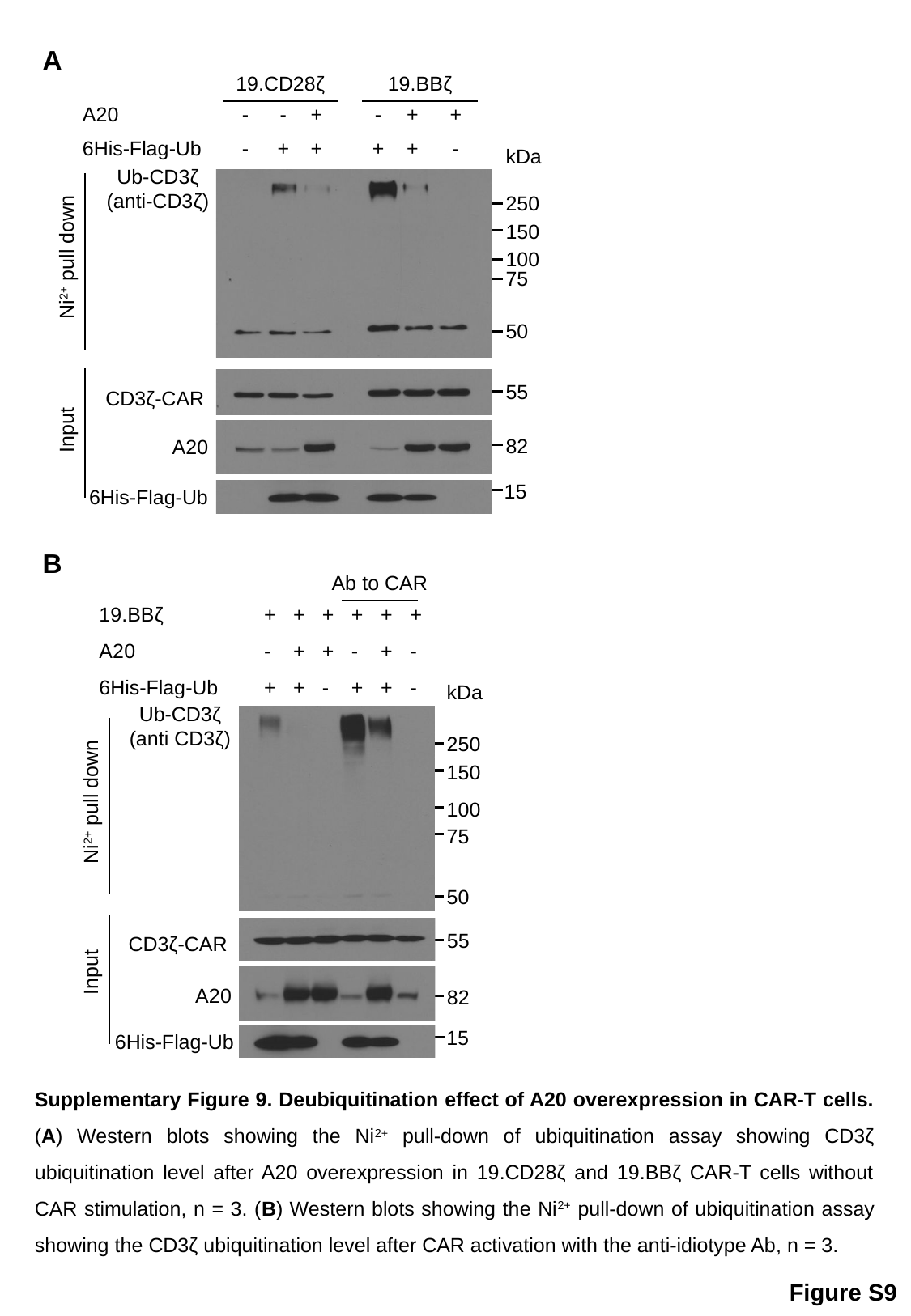

A
19.CD28ζ
19.BBζ
| A20 | - | - | + | | - | + | + |
| --- | --- | --- | --- | --- | --- | --- | --- |
| 6His-Flag-Ub | - | + | + | | + | + | - |
kDa
Ub-CD3ζ
(anti-CD3ζ)
250
150
Ni2+ pull down
100
75
50
55
CD3ζ-CAR
Input
82
A20
15
6His-Flag-Ub
B
Ab to CAR
| 19.BBζ | + | + | + | + | + | + |
| --- | --- | --- | --- | --- | --- | --- |
| A20 | - | + | + | - | + | - |
| 6His-Flag-Ub | + | + | - | + | + | - |
kDa
Ub-CD3ζ
(anti CD3ζ)
250
150
Ni2+ pull down
100
75
50
55
CD3ζ-CAR
Input
A20
82
15
6His-Flag-Ub
Supplementary Figure 9. Deubiquitination effect of A20 overexpression in CAR-T cells. (A) Western blots showing the Ni2+ pull-down of ubiquitination assay showing CD3ζ ubiquitination level after A20 overexpression in 19.CD28ζ and 19.BBζ CAR-T cells without CAR stimulation, n = 3. (B) Western blots showing the Ni2+ pull-down of ubiquitination assay showing the CD3ζ ubiquitination level after CAR activation with the anti-idiotype Ab, n = 3.
Figure S9

## Slide 16
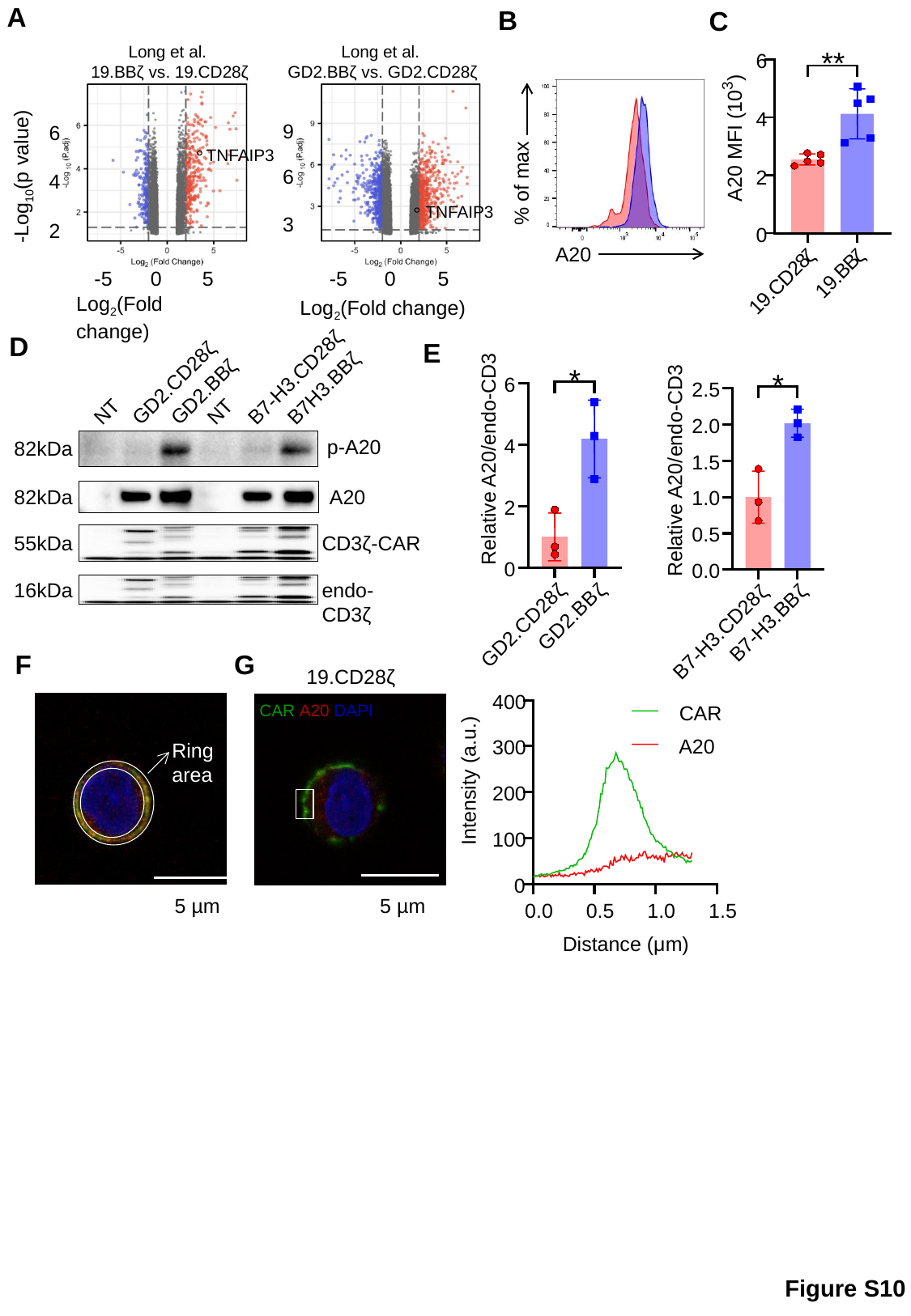

A
B
C
**
6
)
3
4
A20 MFI (10
2
0
ζ
ζ
8
B
2
B
D
.
9
C
1
.
9
1
Long et al.
GD2.BBζ vs. GD2.CD28ζ
Long et al.
19.BBζ vs. 19.CD28ζ
6
-Log10(p value)
4
2
-5
0
5
9
6
3
0
5
-5
TNFAIP3
TNFAIP3
Log2(Fold change)
Log2(Fold change)
% of max
A20
D
E
*
6
4
Relative A20/endo-CD3
2
0
ζ
ζ
8
B
2
B
D
.
2
C
D
.
2
G
D
G
*
2.5
2.0
1.5
Relative A20/endo-CD3
1.0
0.5
0.0
ζ
ζ
8
B
2
B
D
.
3
C
H
.
-
3
7
H
B
-
7
B
B7-H3.CD28ζ
GD2.CD28ζ
B7H3.BBζ
GD2.BBζ
NT
NT
p-A20
82kDa
A20
82kDa
55kDa
CD3ζ-CAR
16kDa
endo-CD3ζ
F
G
19.CD28ζ
CAR A20 DAPI
400
CAR
A20
300
Intensity (a.u.)
200
100
0
0.0
0.5
1.0
1.5
Distance (μm)
Ring area
5 µm
5 µm
Figure S10

## Slide 17
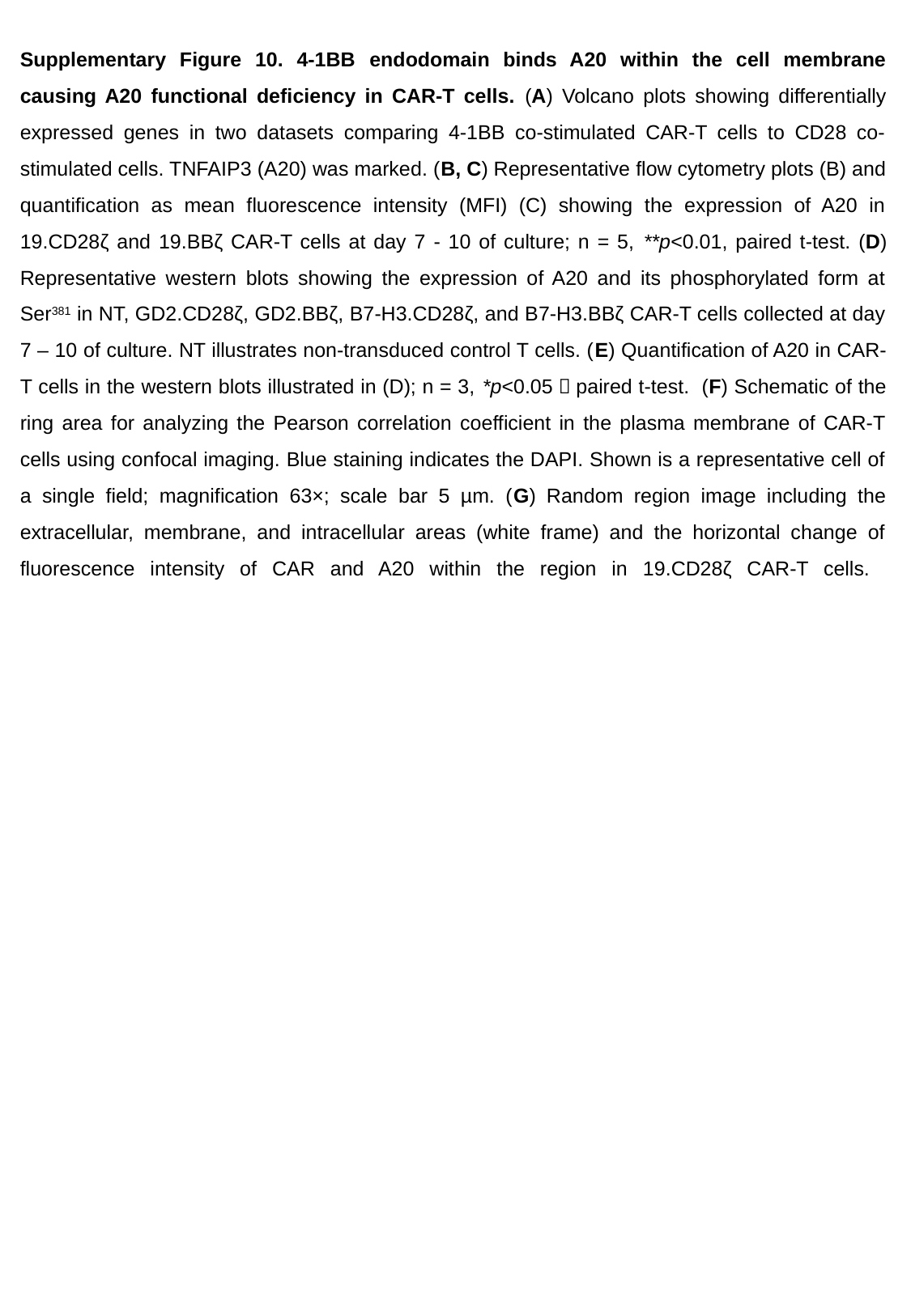

Supplementary Figure 10. 4-1BB endodomain binds A20 within the cell membrane causing A20 functional deficiency in CAR-T cells. (A) Volcano plots showing differentially expressed genes in two datasets comparing 4-1BB co-stimulated CAR-T cells to CD28 co-stimulated cells. TNFAIP3 (A20) was marked. (B, C) Representative flow cytometry plots (B) and quantification as mean fluorescence intensity (MFI) (C) showing the expression of A20 in 19.CD28ζ and 19.BBζ CAR-T cells at day 7 - 10 of culture; n = 5, **p<0.01, paired t-test. (D) Representative western blots showing the expression of A20 and its phosphorylated form at Ser381 in NT, GD2.CD28ζ, GD2.BBζ, B7-H3.CD28ζ, and B7-H3.BBζ CAR-T cells collected at day 7 – 10 of culture. NT illustrates non-transduced control T cells. (E) Quantification of A20 in CAR-T cells in the western blots illustrated in (D); n = 3, *p<0.05，paired t-test. (F) Schematic of the ring area for analyzing the Pearson correlation coefficient in the plasma membrane of CAR-T cells using confocal imaging. Blue staining indicates the DAPI. Shown is a representative cell of a single field; magnification 63×; scale bar 5 µm. (G) Random region image including the extracellular, membrane, and intracellular areas (white frame) and the horizontal change of fluorescence intensity of CAR and A20 within the region in 19.CD28ζ CAR-T cells.

## Slide 18
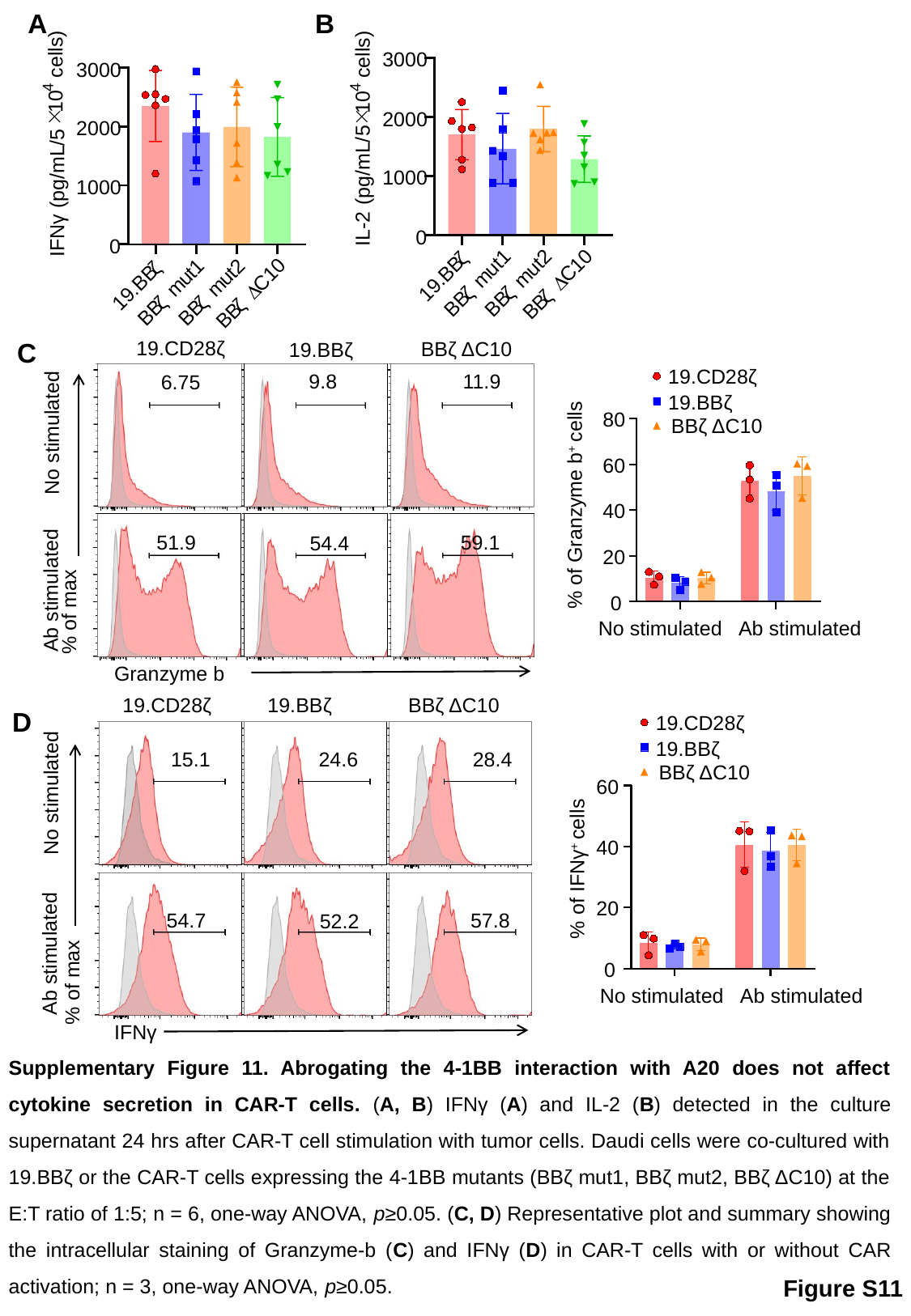

A
B
 cells)
3000
4
10
´
2000
1000
IFNγ (pg/mL/5
0
0
1
2
ζ
t
t
B
1
u
u
B
C
m
m
.
D
9
1
ζ
ζ
ζ
B
B
B
B
B
B
 cells)
3000
4
10
´
2000
1000
IL-2 (pg/mL/5
0
0
1
2
ζ
t
t
B
1
u
u
B
C
m
m
.
D
9
1
ζ
ζ
ζ
B
B
B
B
B
B
19.CD28ζ
BBζ ΔC10
19.BBζ
9.8
11.9
6.75
No stimulated
59.1
51.9
54.4
Ab stimulated
% of max
Granzyme b
C
19.CD28ζ
19.BBζ
BBζ ΔC10
80
60
% of Granzyme b+ cells
40
20
0
Ab stimulated
No stimulated
19.CD28ζ
19.BBζ
BBζ ΔC10
24.6
28.4
15.1
Ab stimulated
57.8
54.7
52.2
% of max
No stimulated
IFNγ
19.CD28ζ
19.BBζ
BBζ ΔC10
60
% of IFNγ+ cells
40
20
0
No stimulated
Ab stimulated
D
Supplementary Figure 11. Abrogating the 4-1BB interaction with A20 does not affect cytokine secretion in CAR-T cells. (A, B) IFNγ (A) and IL-2 (B) detected in the culture supernatant 24 hrs after CAR-T cell stimulation with tumor cells. Daudi cells were co-cultured with 19.BBζ or the CAR-T cells expressing the 4-1BB mutants (BBζ mut1, BBζ mut2, BBζ ΔC10) at the E:T ratio of 1:5; n = 6, one-way ANOVA, p≥0.05. (C, D) Representative plot and summary showing the intracellular staining of Granzyme-b (C) and IFNγ (D) in CAR-T cells with or without CAR activation; n = 3, one-way ANOVA, p≥0.05.
Figure S11

## Slide 19
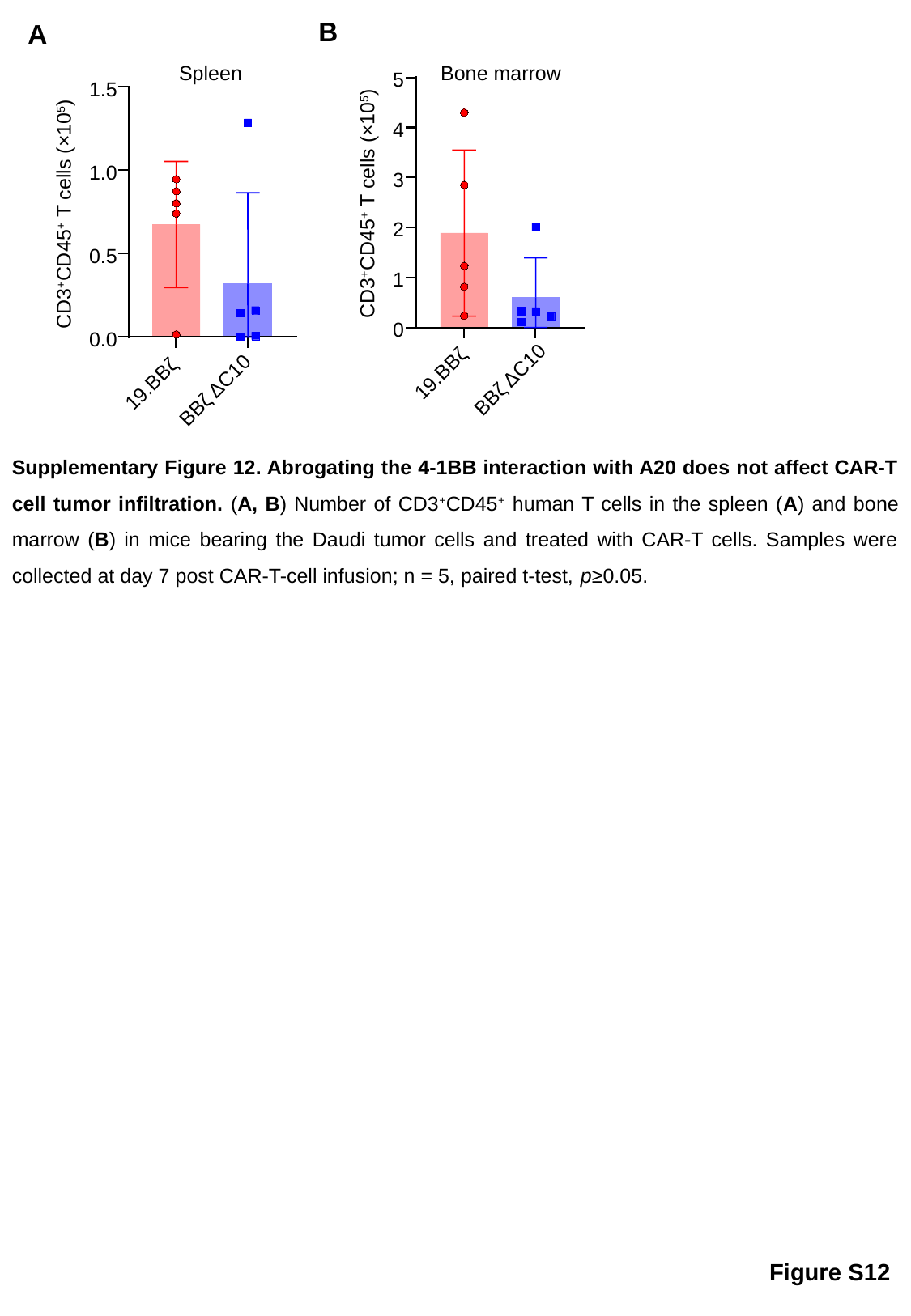

B
A
Bone marrow
5
4
CD3+CD45+ T cells (×105)
3
2
1
0
19.BBζ
BBζ ΔC10
Spleen
1.5
1.0
CD3+CD45+ T cells (×105)
0.5
0.0
19.BBζ
BBζ ΔC10
Supplementary Figure 12. Abrogating the 4-1BB interaction with A20 does not affect CAR-T cell tumor infiltration. (A, B) Number of CD3+CD45+ human T cells in the spleen (A) and bone marrow (B) in mice bearing the Daudi tumor cells and treated with CAR-T cells. Samples were collected at day 7 post CAR-T-cell infusion; n = 5, paired t-test, p≥0.05.
Figure S12
