# Supplementary material for: 4-1BB-encoding CAR causes cell death via sequestration of the ubiquitin-modifying enzyme A20
Source: Cell Mol Immunol. 2024 Jun 27;21(8):905–17. doi: 10.1038/s41423-024-01198-y (PMC11291893; doi:10.1038/s41423-024-01198-y)
Supplement: Supplementary file 2 — Uncrop western files [file 41423_2024_1198_MOESM2_ESM.pptx]

## Slide 1
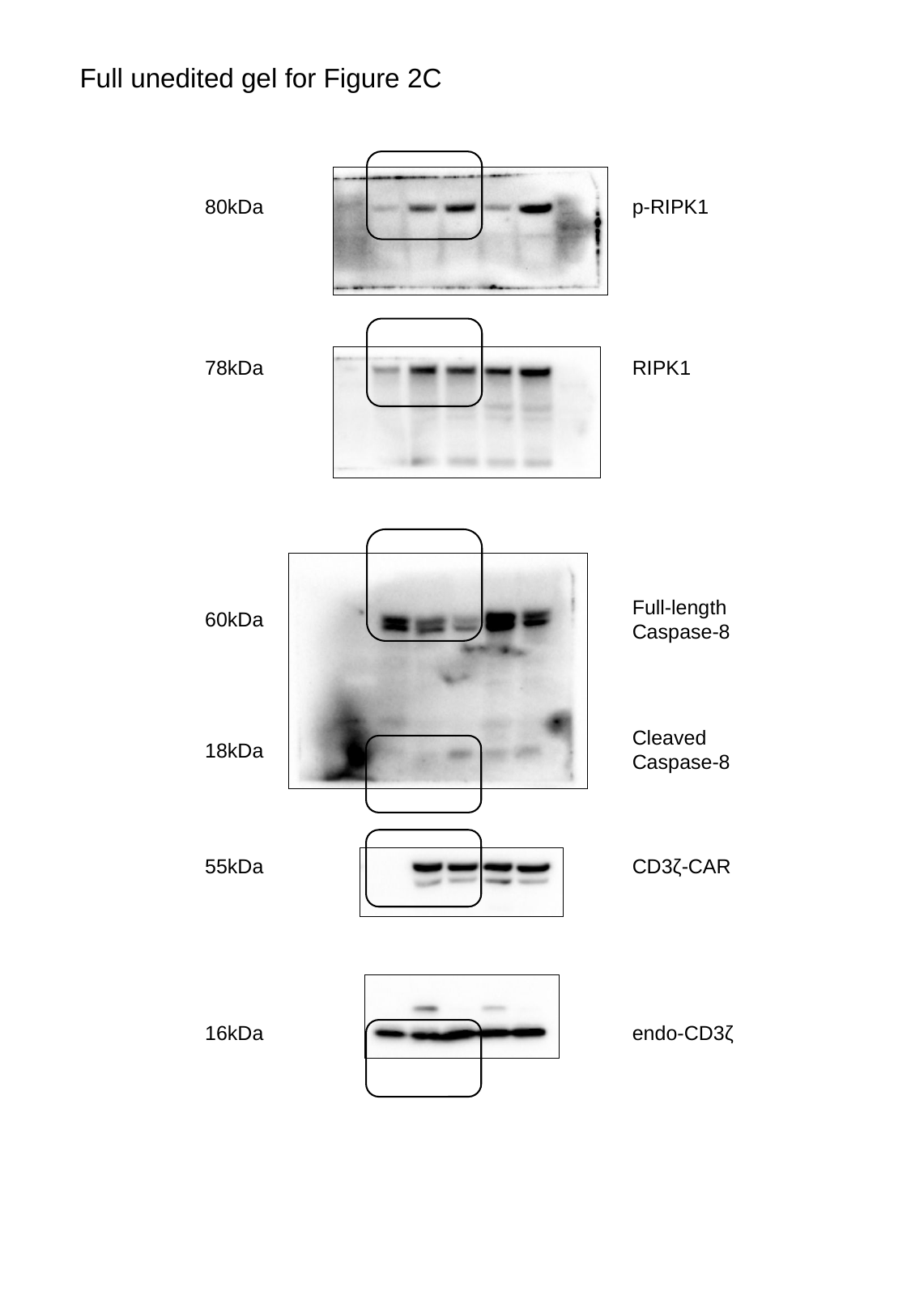

Full unedited gel for Figure 2C
80kDa
p-RIPK1
78kDa
RIPK1
Full-length Caspase-8
60kDa
Cleaved Caspase-8
18kDa
55kDa
CD3ζ-CAR
16kDa
endo-CD3ζ

## Slide 2
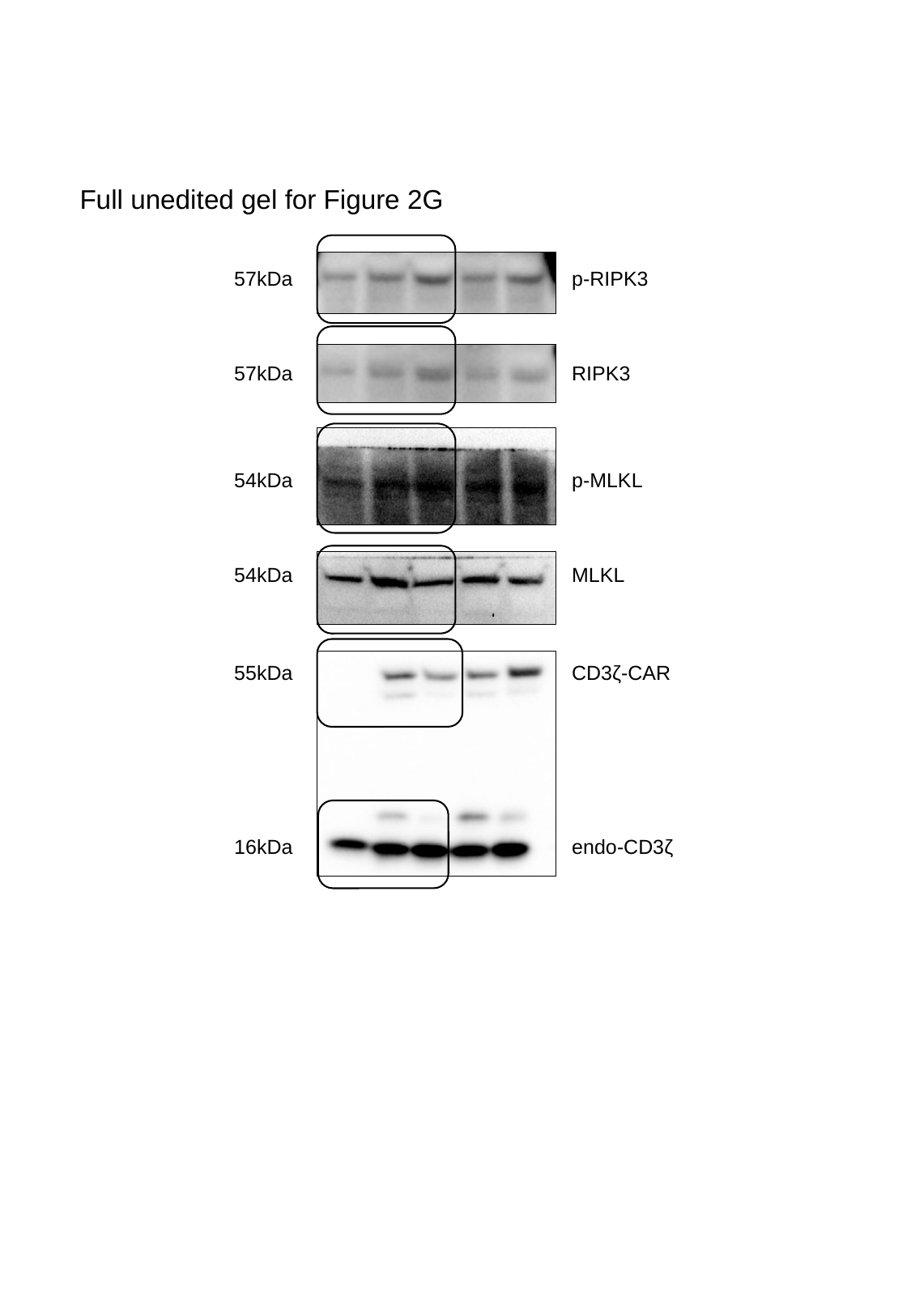

Full unedited gel for Figure 2G
57kDa
p-RIPK3
57kDa
RIPK3
54kDa
p-MLKL
54kDa
MLKL
55kDa
CD3ζ-CAR
16kDa
endo-CD3ζ

## Slide 3
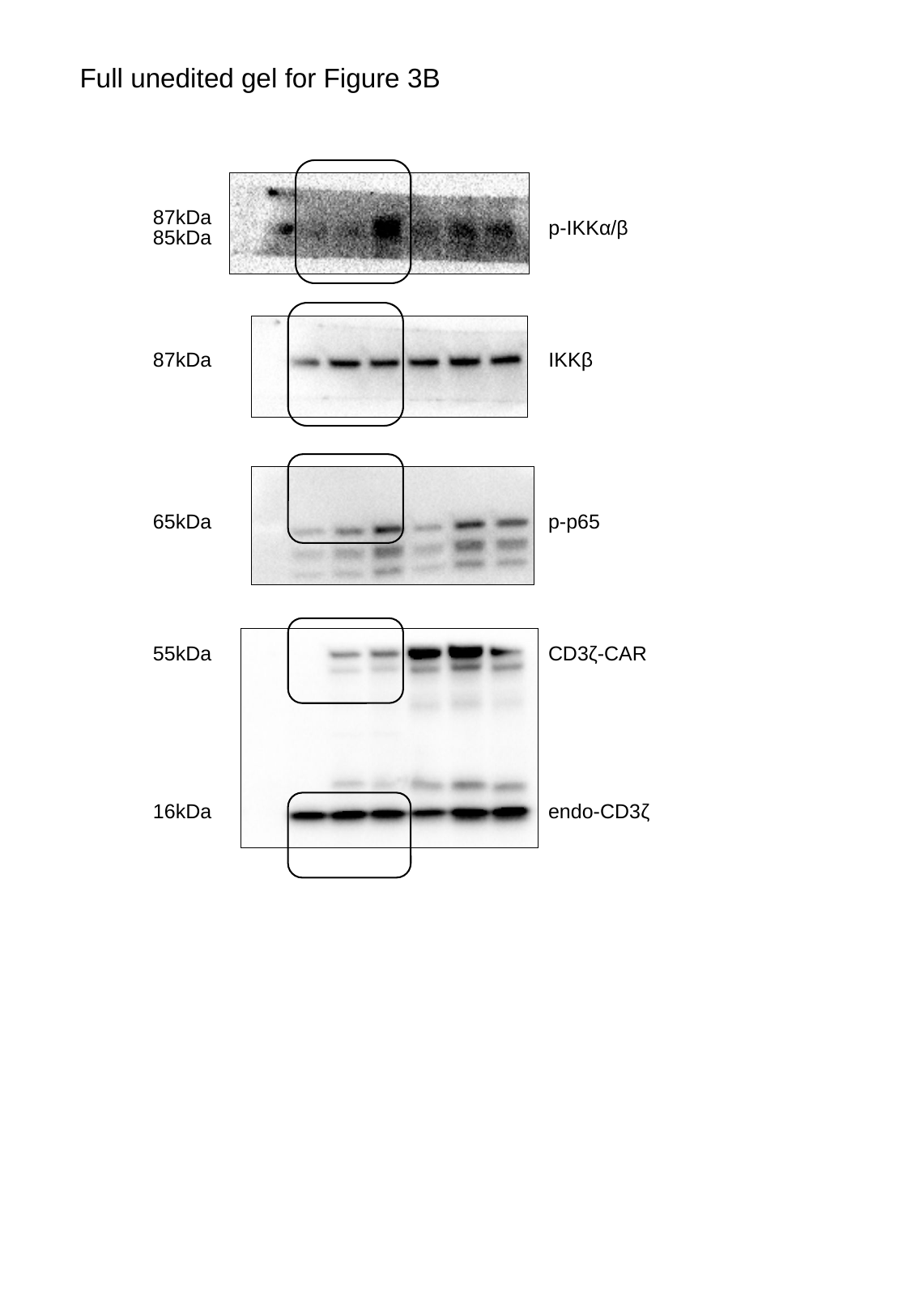

Full unedited gel for Figure 3B
87kDa
p-IKKα/β
85kDa
87kDa
IKKβ
65kDa
p-p65
55kDa
CD3ζ-CAR
16kDa
endo-CD3ζ

## Slide 4
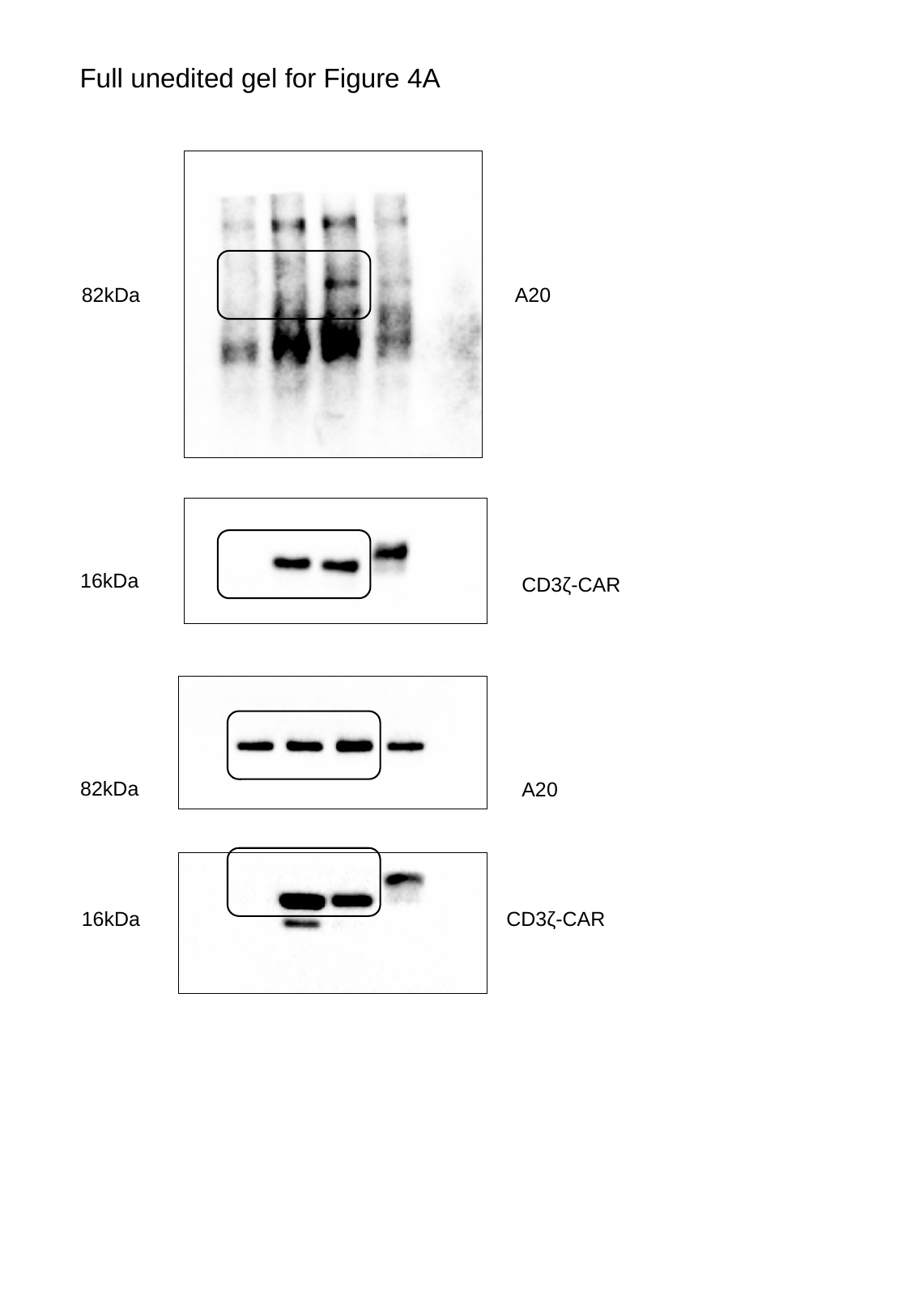

Full unedited gel for Figure 4A
82kDa
A20
16kDa
CD3ζ-CAR
82kDa
A20
16kDa
CD3ζ-CAR

## Slide 5
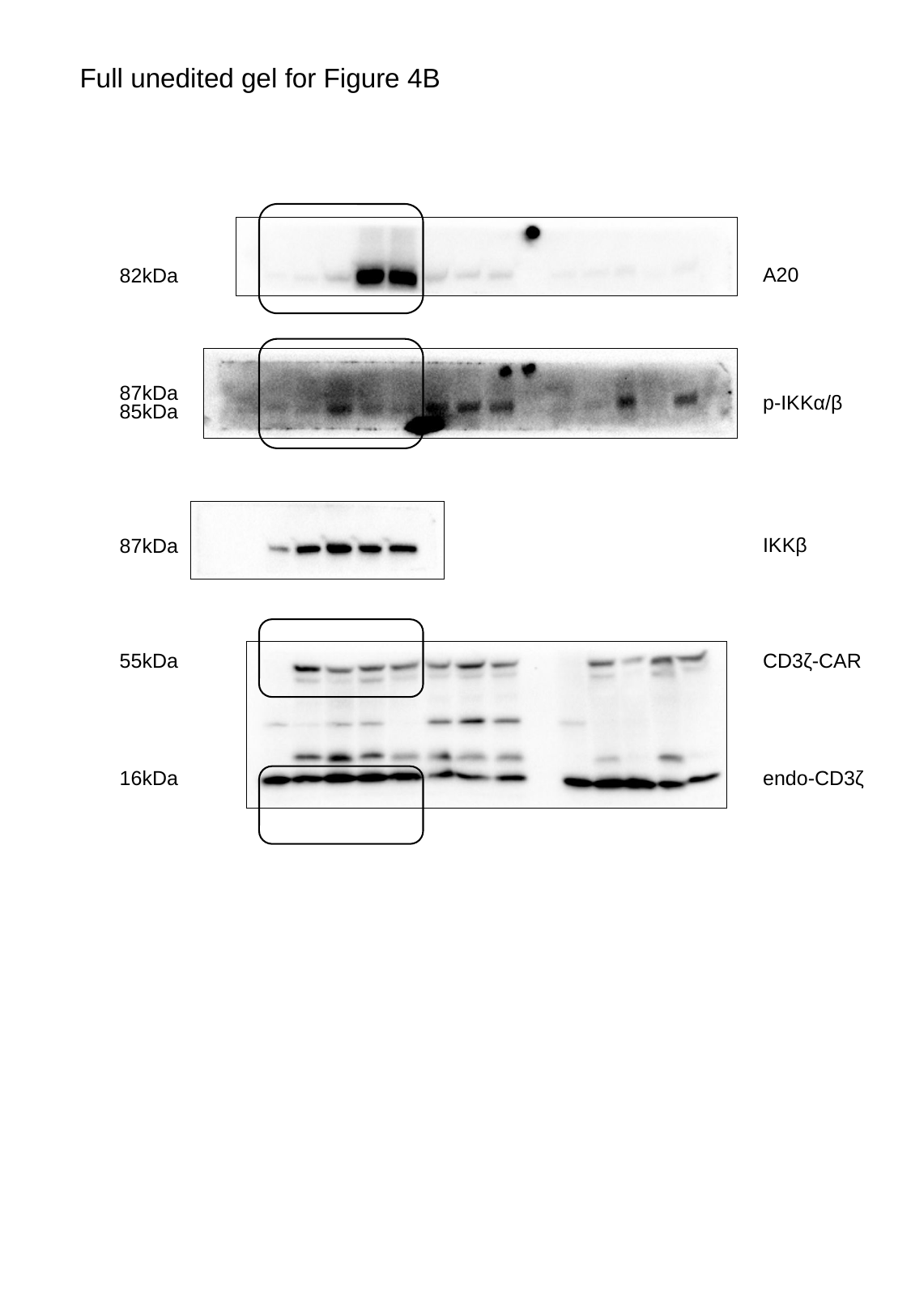

Full unedited gel for Figure 4B
A20
82kDa
87kDa
p-IKKα/β
85kDa
IKKβ
87kDa
CD3ζ-CAR
55kDa
endo-CD3ζ
16kDa

## Slide 6
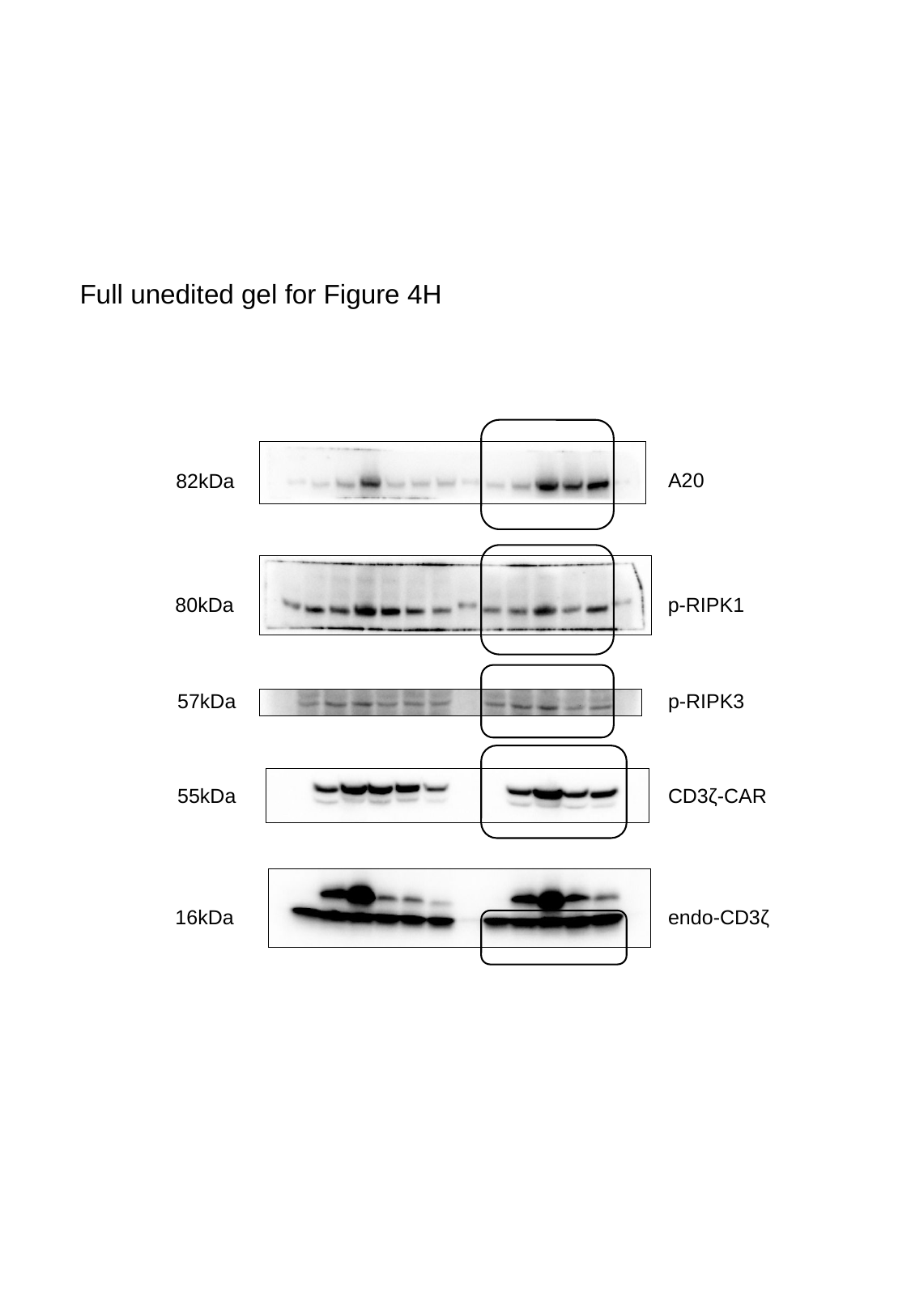

Full unedited gel for Figure 4H
A20
82kDa
80kDa
p-RIPK1
57kDa
p-RIPK3
55kDa
CD3ζ-CAR
16kDa
endo-CD3ζ

## Slide 7
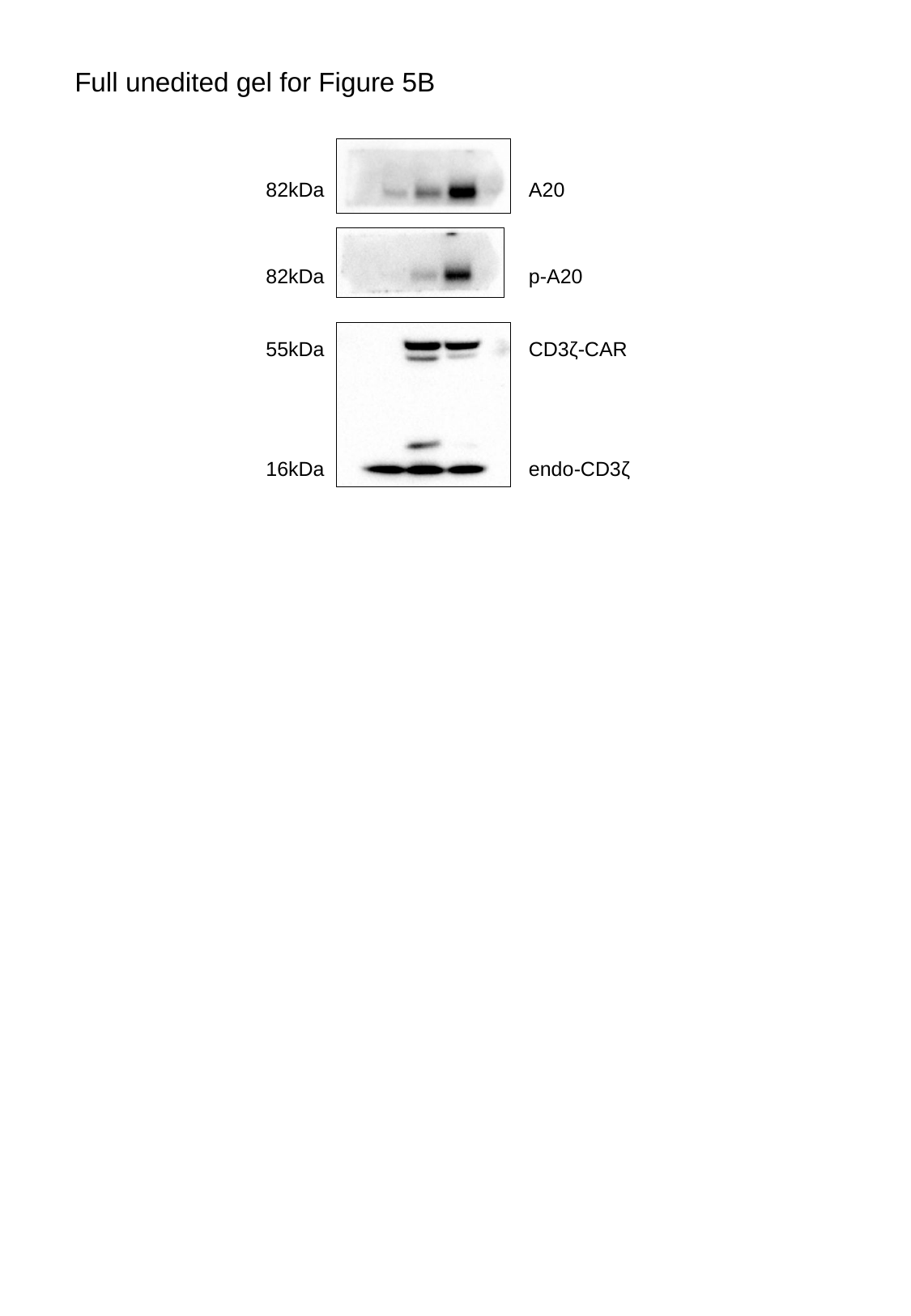

Full unedited gel for Figure 5B
82kDa
A20
82kDa
p-A20
55kDa
CD3ζ-CAR
16kDa
endo-CD3ζ

## Slide 8
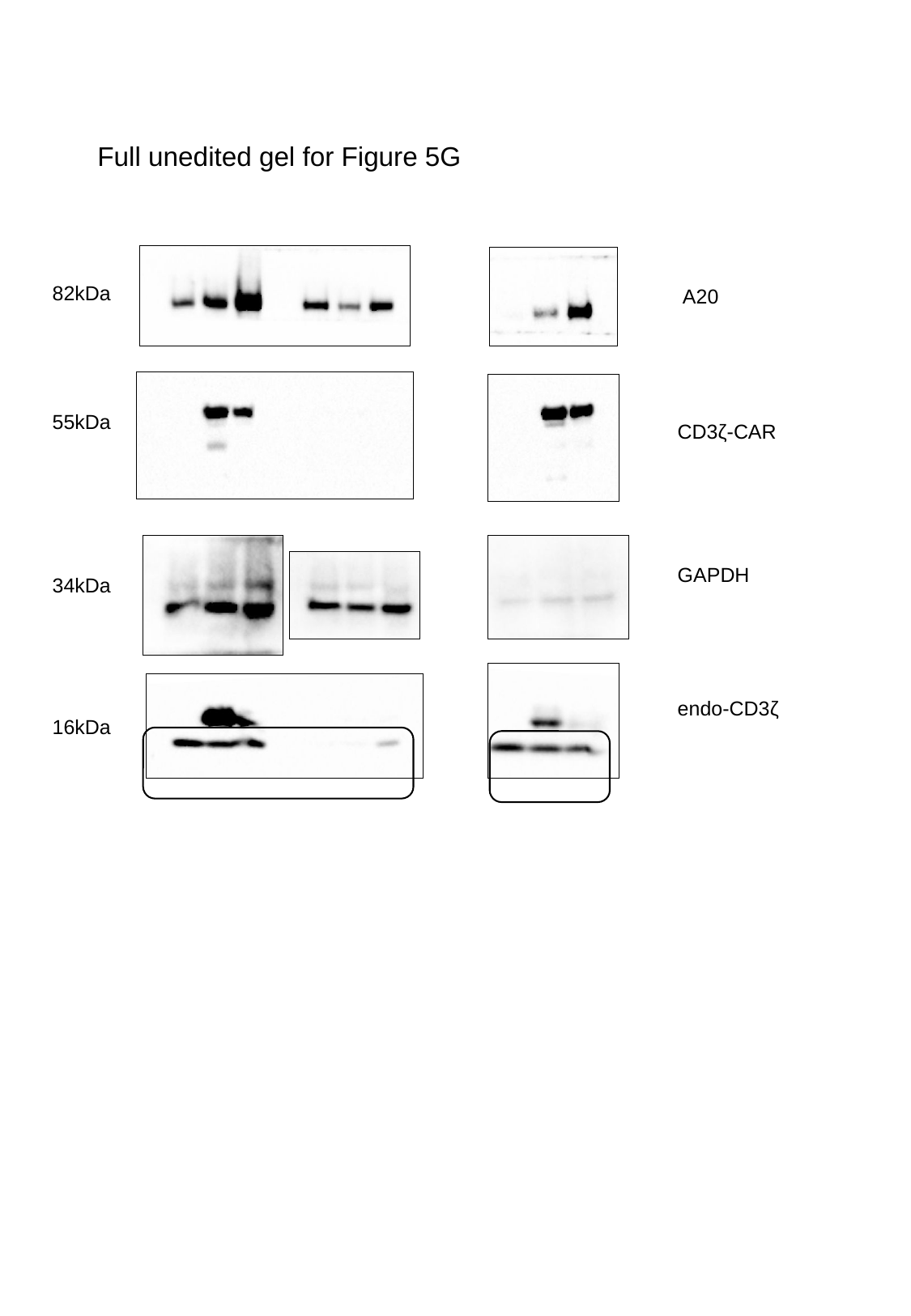

Full unedited gel for Figure 5G
82kDa
A20
55kDa
CD3ζ-CAR
GAPDH
34kDa
endo-CD3ζ
16kDa

## Slide 9
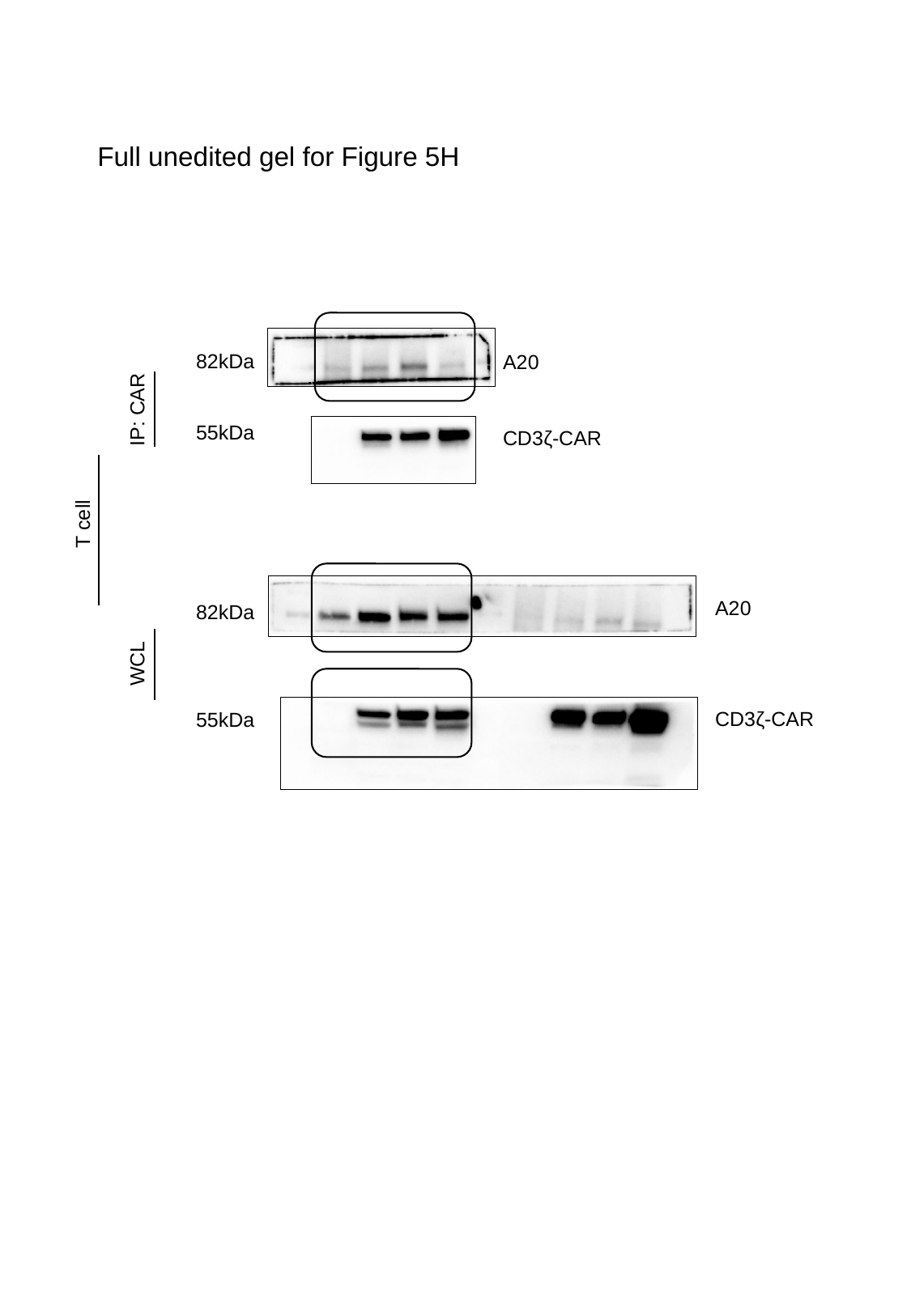

Full unedited gel for Figure 5H
82kDa
A20
IP: CAR
55kDa
CD3ζ-CAR
T cell
A20
82kDa
WCL
CD3ζ-CAR
55kDa

## Slide 10
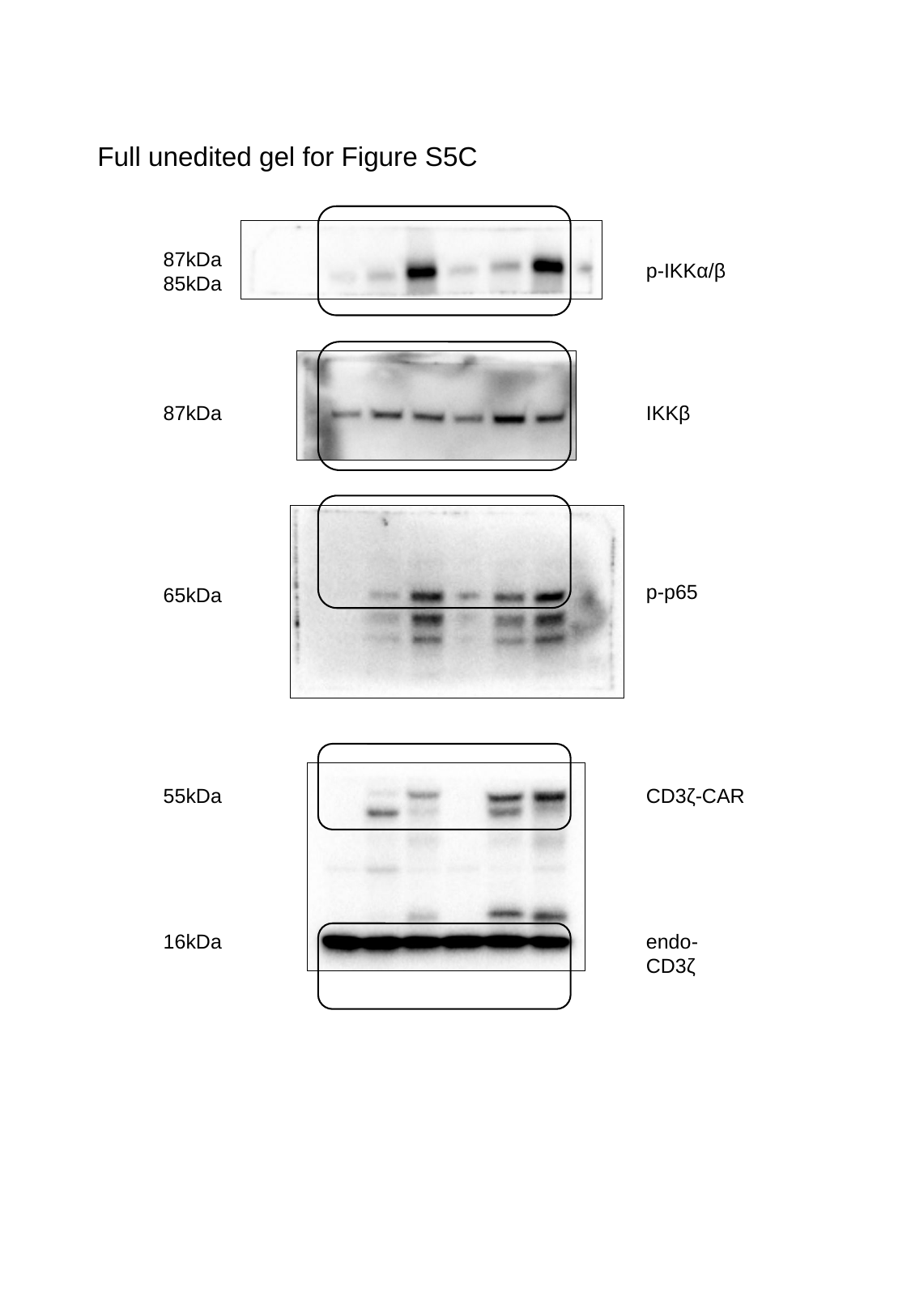

Full unedited gel for Figure S5C
87kDa
p-IKKα/β
85kDa
87kDa
IKKβ
p-p65
65kDa
55kDa
CD3ζ-CAR
16kDa
endo-CD3ζ

## Slide 11
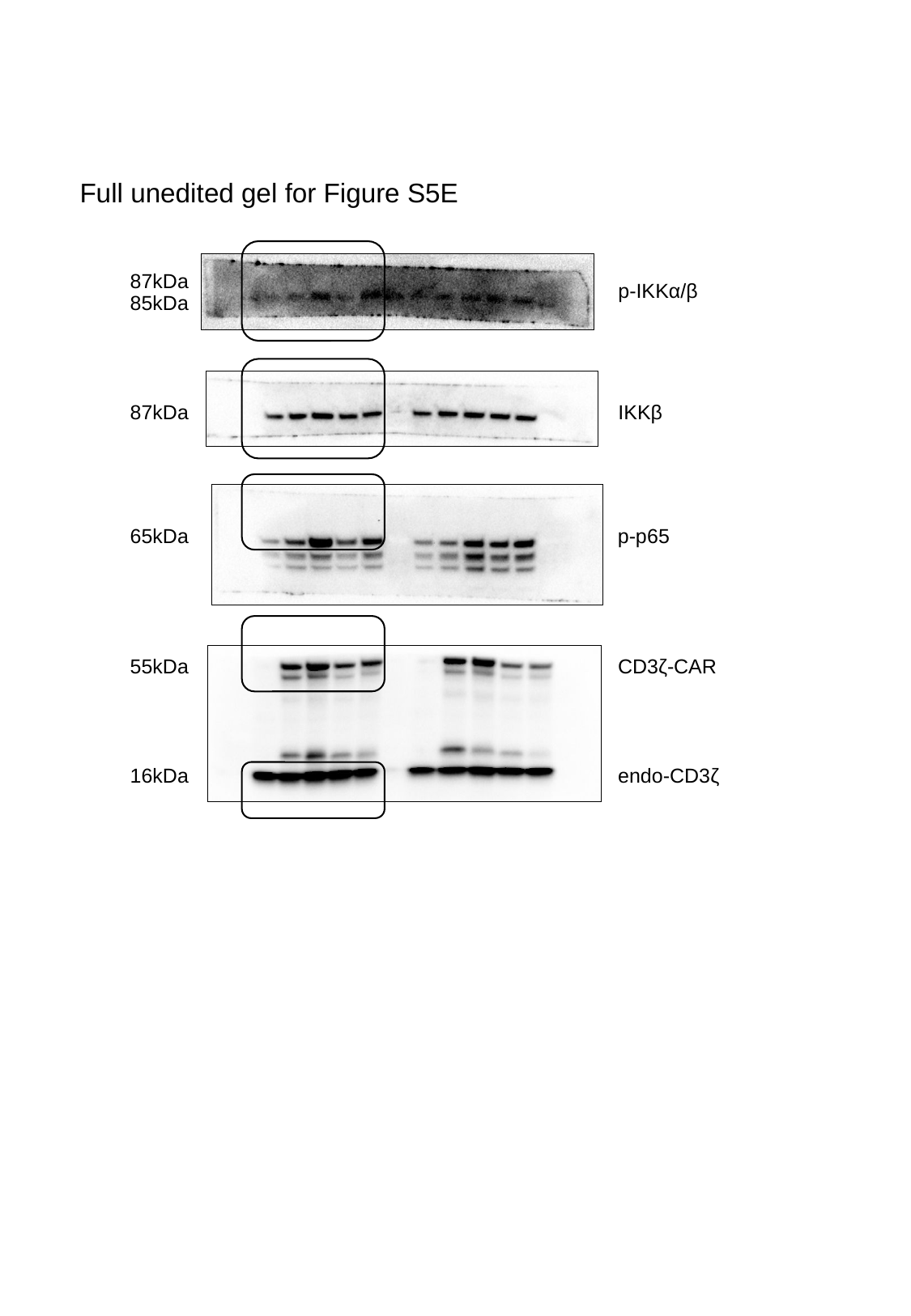

Full unedited gel for Figure S5E
87kDa
p-IKKα/β
85kDa
87kDa
IKKβ
65kDa
p-p65
55kDa
CD3ζ-CAR
16kDa
endo-CD3ζ

## Slide 12
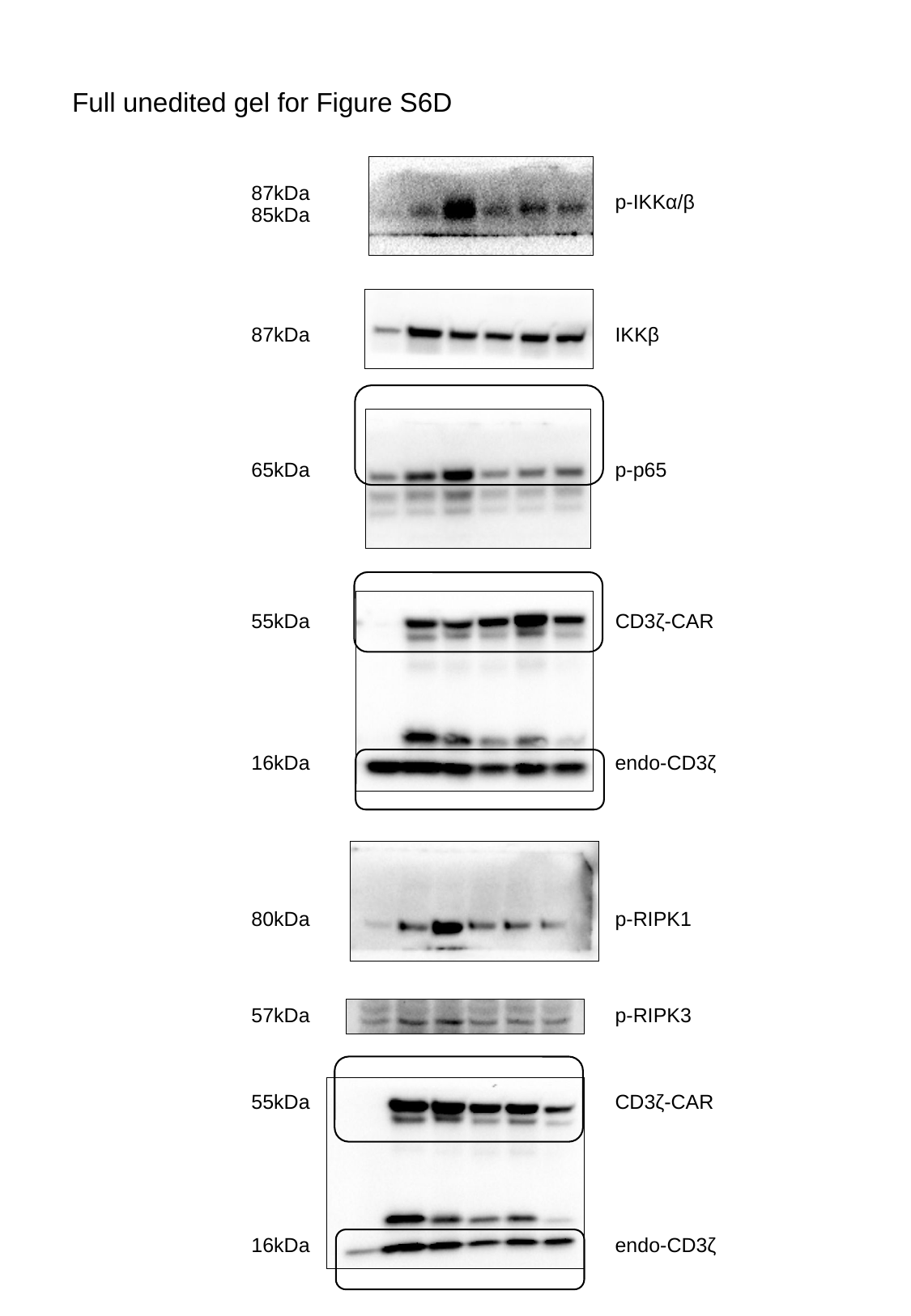

Full unedited gel for Figure S6D
87kDa
p-IKKα/β
85kDa
87kDa
IKKβ
65kDa
p-p65
55kDa
CD3ζ-CAR
16kDa
endo-CD3ζ
80kDa
p-RIPK1
57kDa
p-RIPK3
55kDa
CD3ζ-CAR
16kDa
endo-CD3ζ

## Slide 13
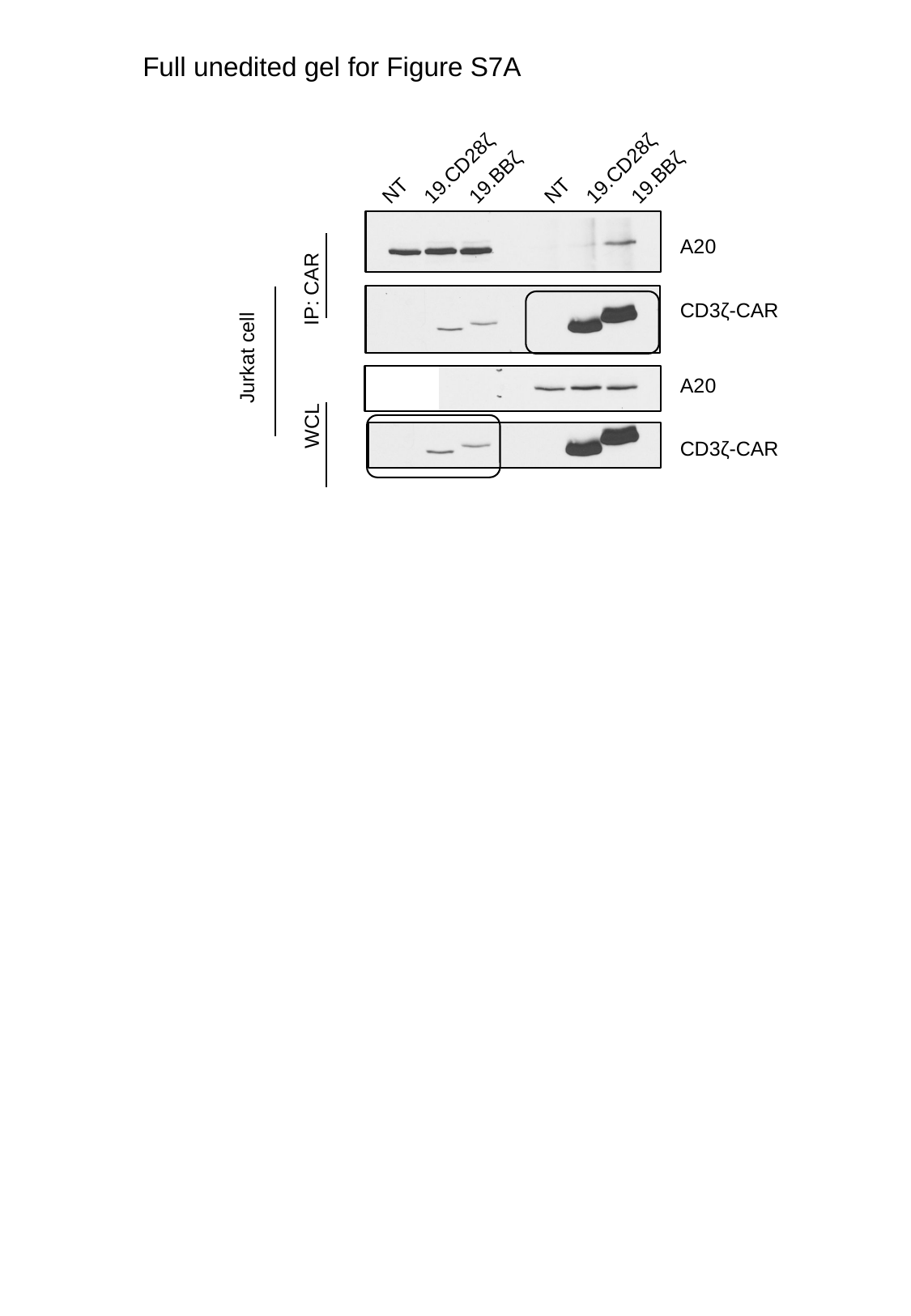

Full unedited gel for Figure S7A
19.CD28ζ
19.CD28ζ
19.BBζ
19.BBζ
NT
NT
A20
IP: CAR
CD3ζ-CAR
Jurkat cell
A20
WCL
CD3ζ-CAR

## Slide 14
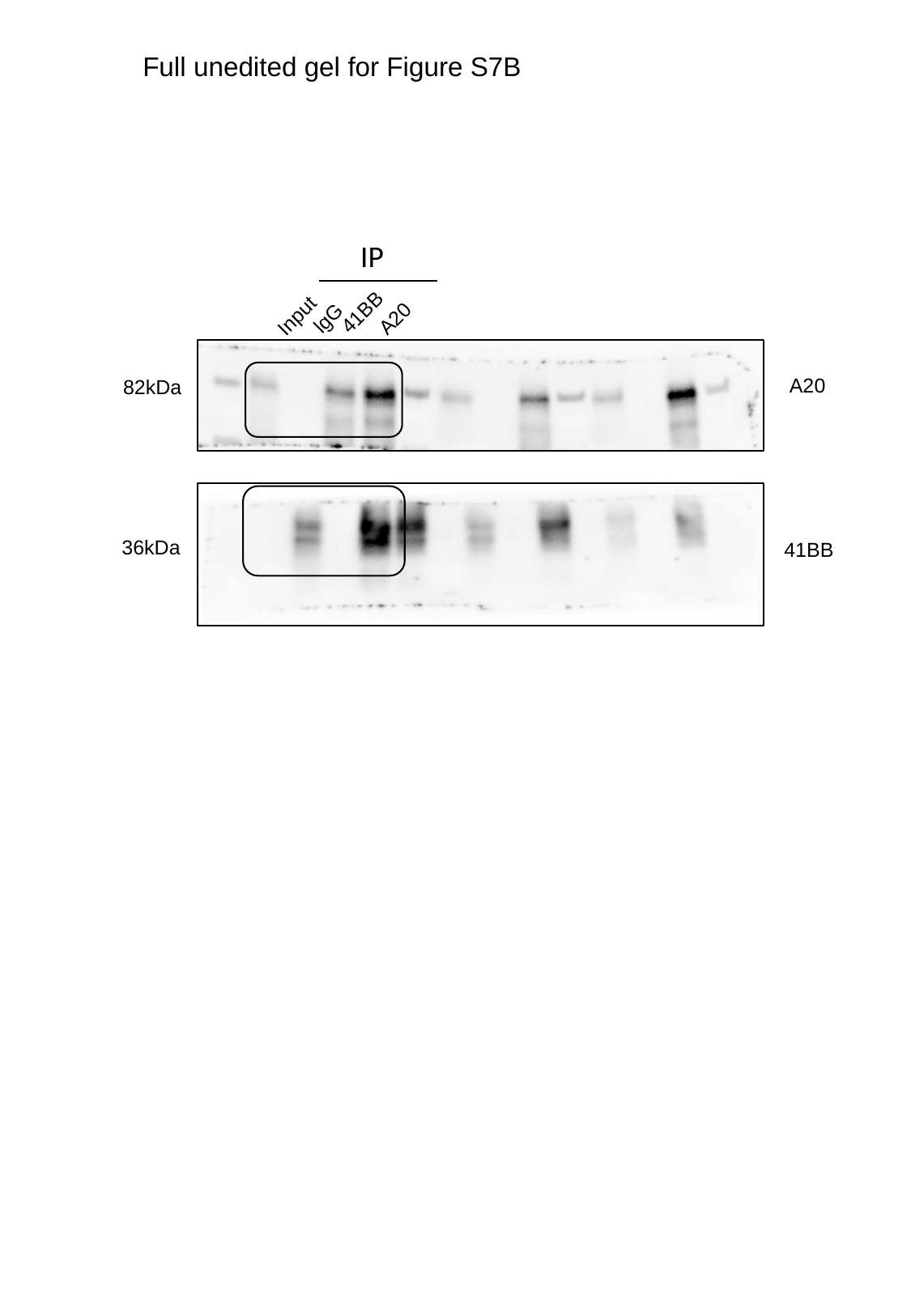

Full unedited gel for Figure S7B
IP
lgG
Input
41BB
A20
A20
82kDa
36kDa
41BB

## Slide 15
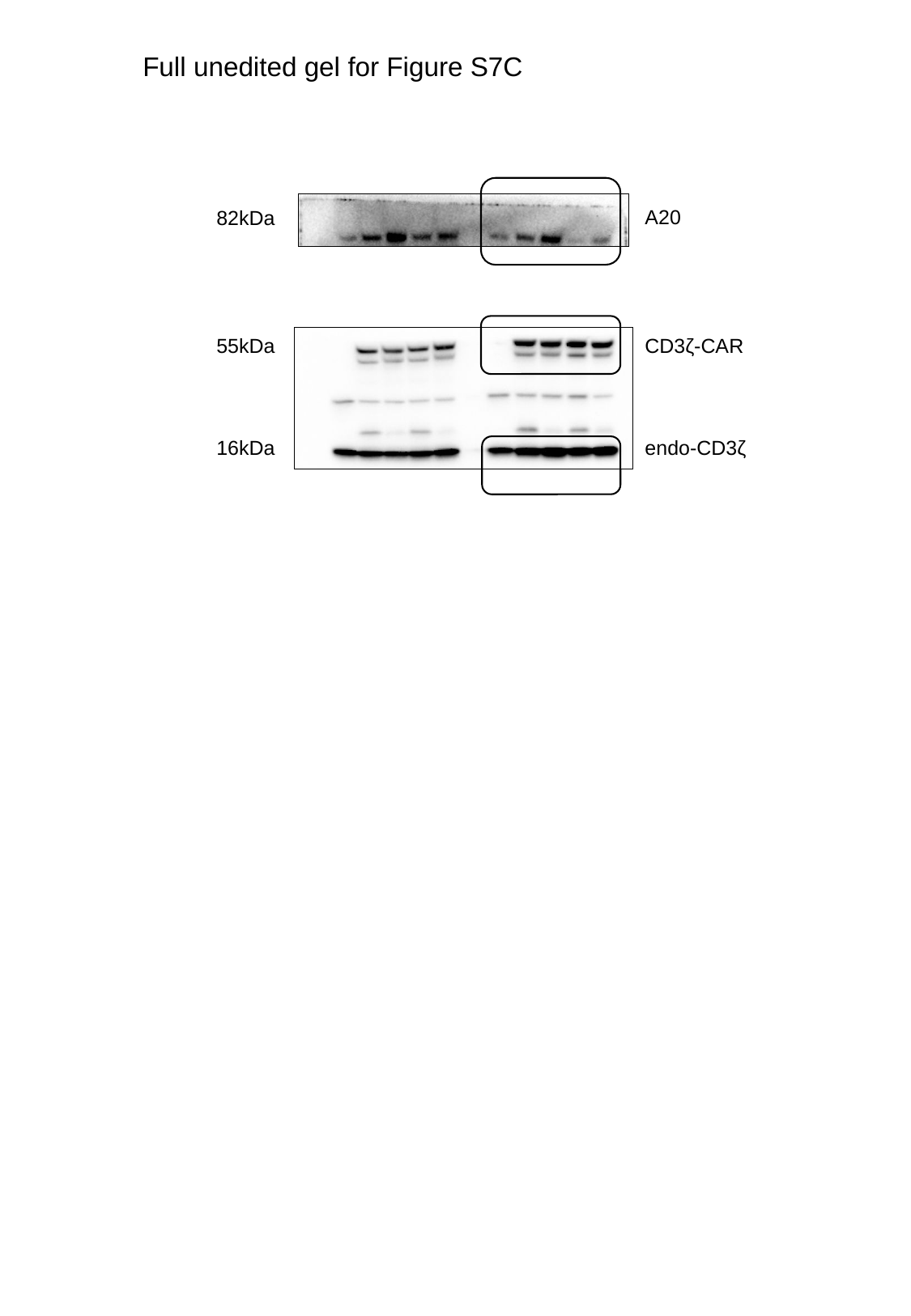

Full unedited gel for Figure S7C
A20
82kDa
CD3ζ-CAR
55kDa
endo-CD3ζ
16kDa

## Slide 16
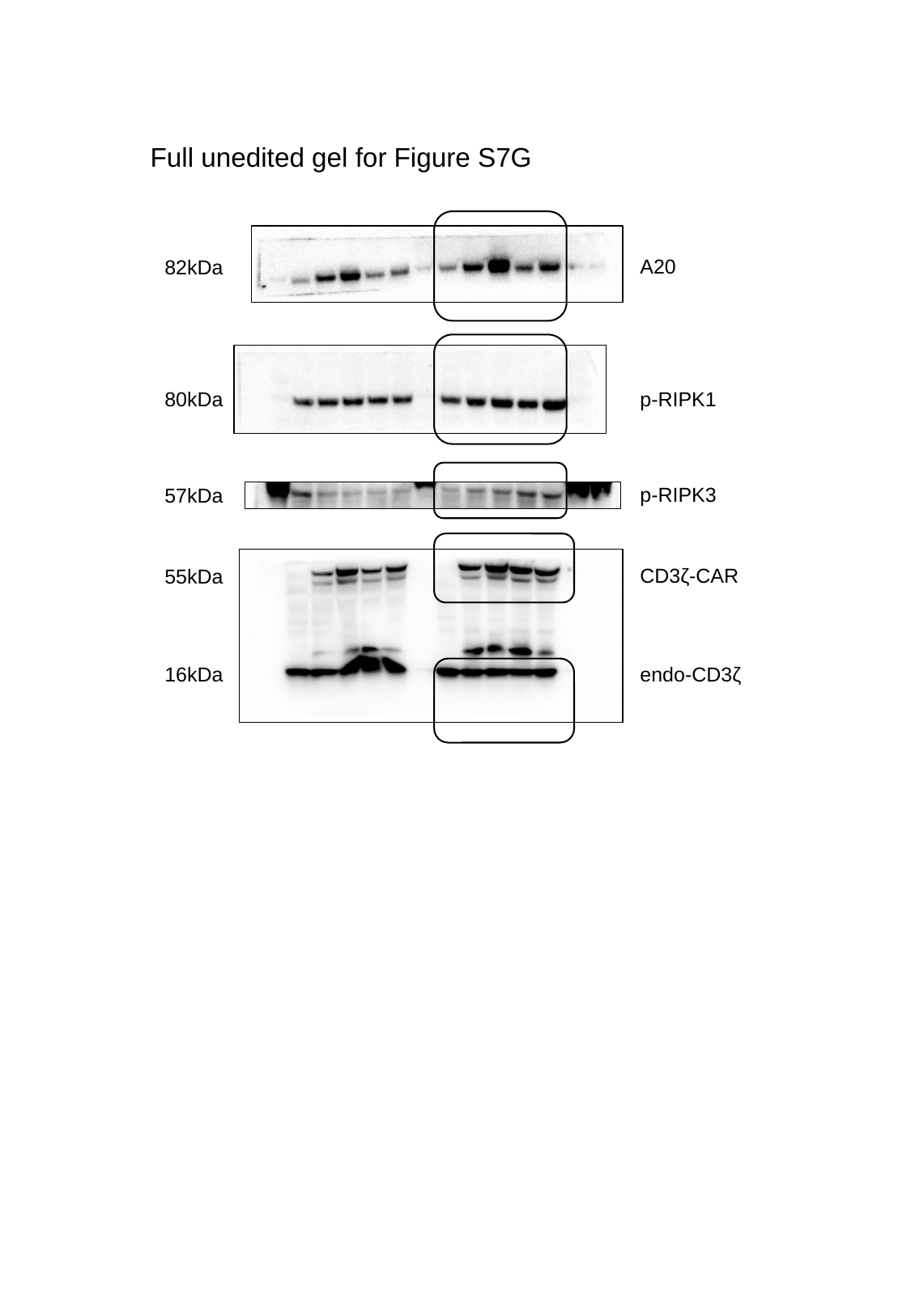

Full unedited gel for Figure S7G
A20
82kDa
80kDa
p-RIPK1
p-RIPK3
57kDa
CD3ζ-CAR
55kDa
endo-CD3ζ
16kDa

## Slide 17
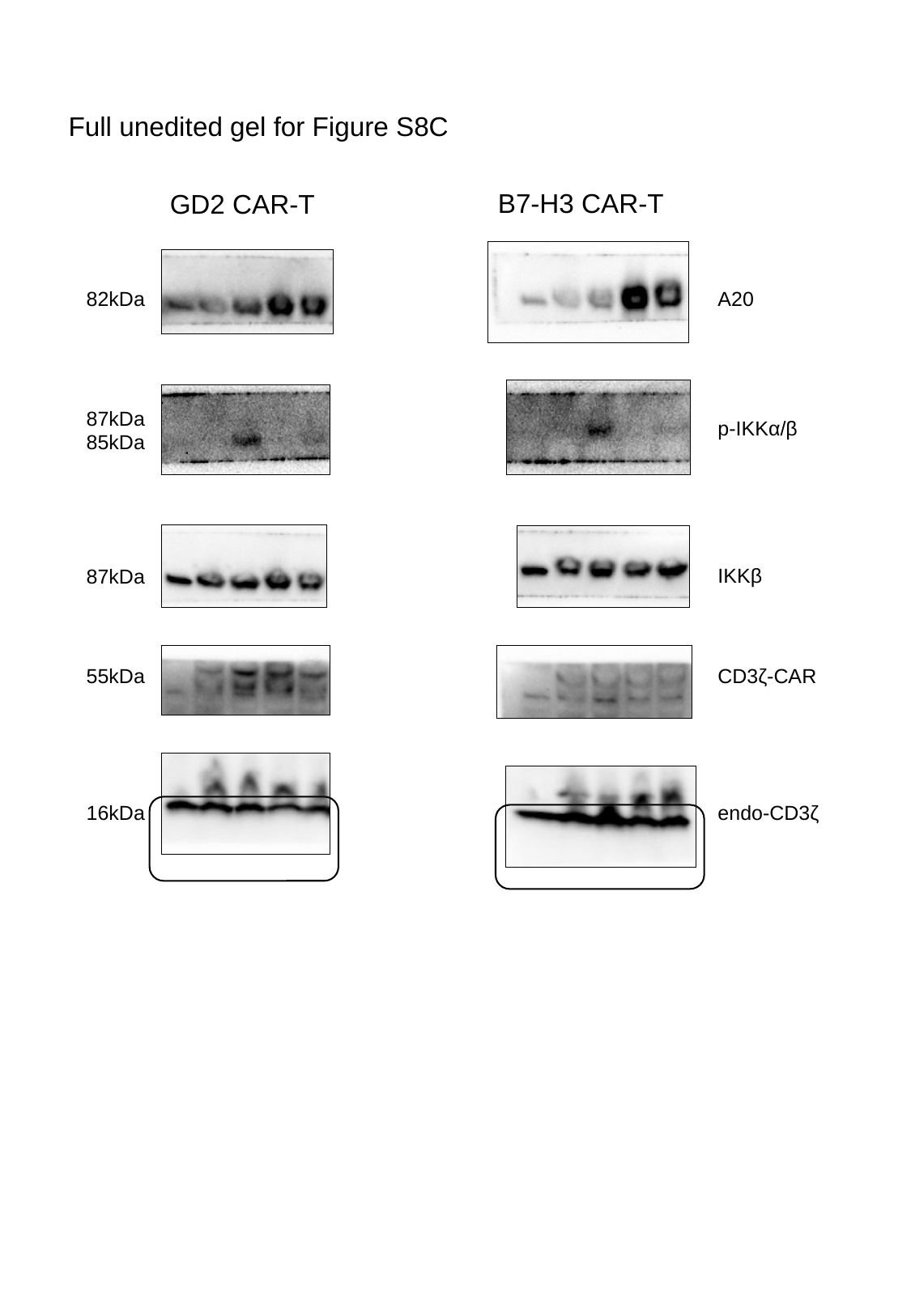

Full unedited gel for Figure S8C
B7-H3 CAR-T
GD2 CAR-T
A20
82kDa
87kDa
p-IKKα/β
85kDa
IKKβ
87kDa
CD3ζ-CAR
55kDa
endo-CD3ζ
16kDa

## Slide 18
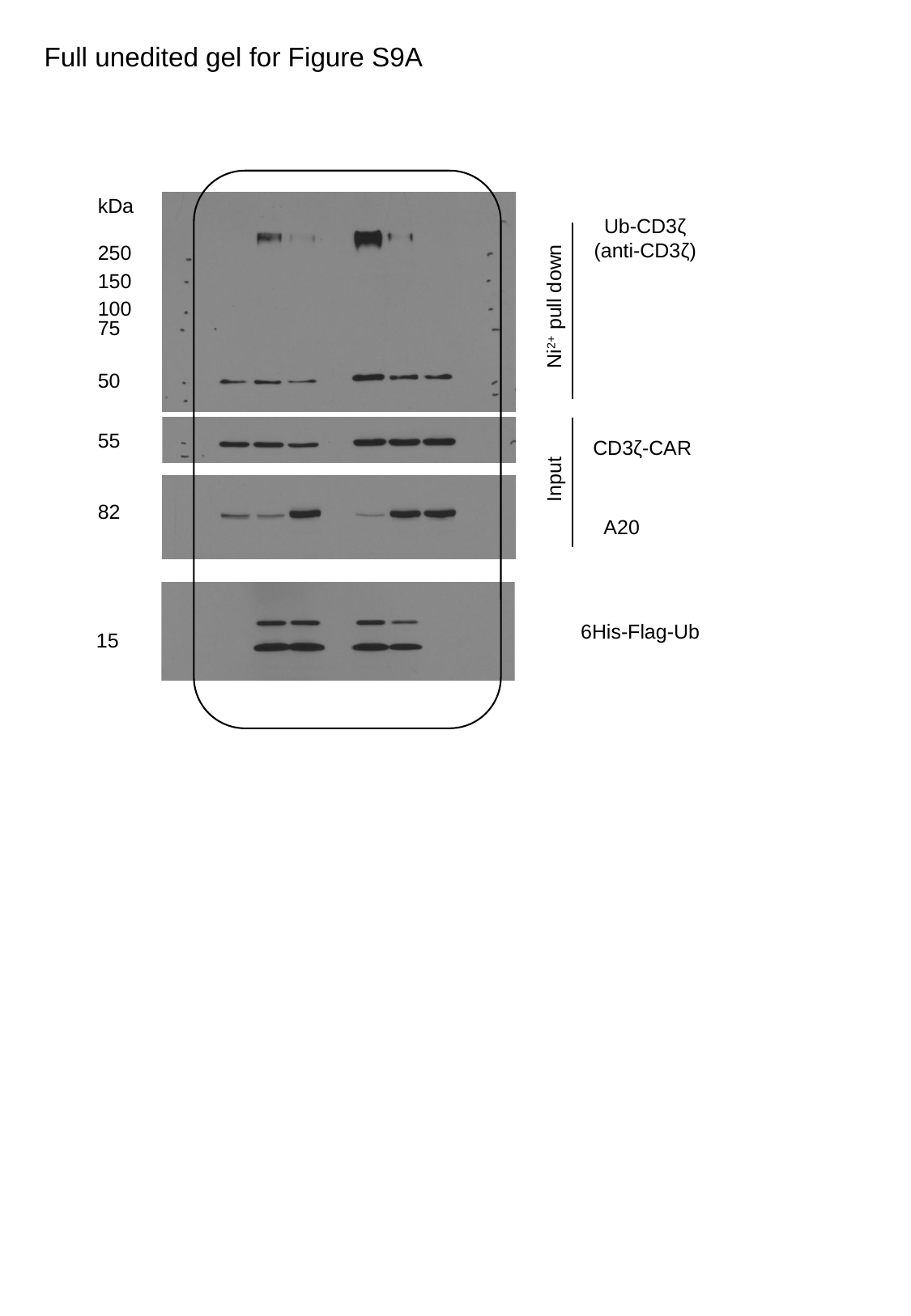

Full unedited gel for Figure S9A
kDa
Ub-CD3ζ
(anti-CD3ζ)
250
150
Ni2+ pull down
100
75
50
55
CD3ζ-CAR
Input
82
A20
6His-Flag-Ub
15

## Slide 19
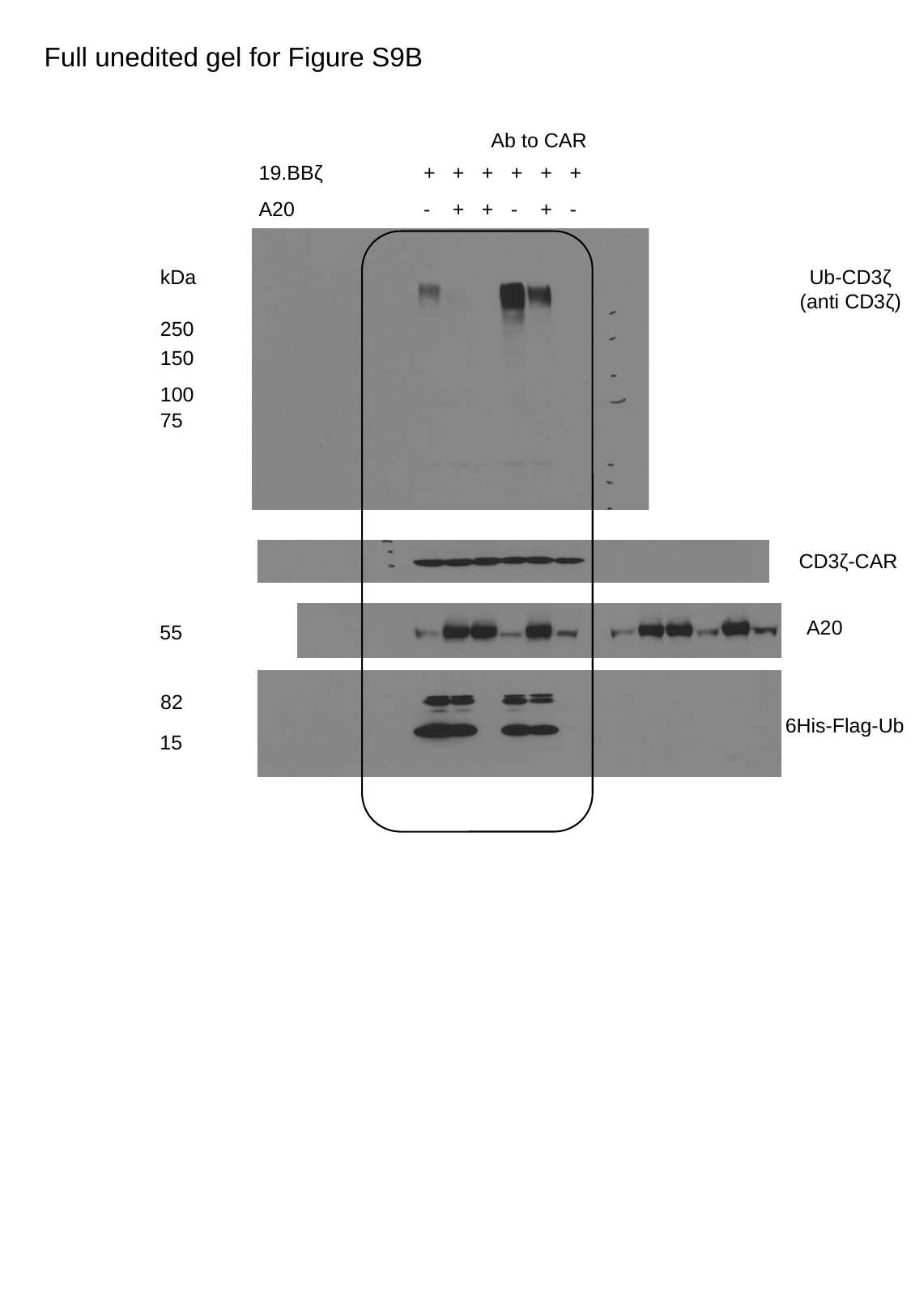

Full unedited gel for Figure S9B
Ab to CAR
| 19.BBζ | + | + | + | + | + | + |
| --- | --- | --- | --- | --- | --- | --- |
| A20 | - | + | + | - | + | - |
| 6His-Flag-Ub | + | + | - | + | + | - |
kDa
Ub-CD3ζ
(anti CD3ζ)
250
150
100
75
CD3ζ-CAR
A20
55
82
6His-Flag-Ub
15

## Slide 20
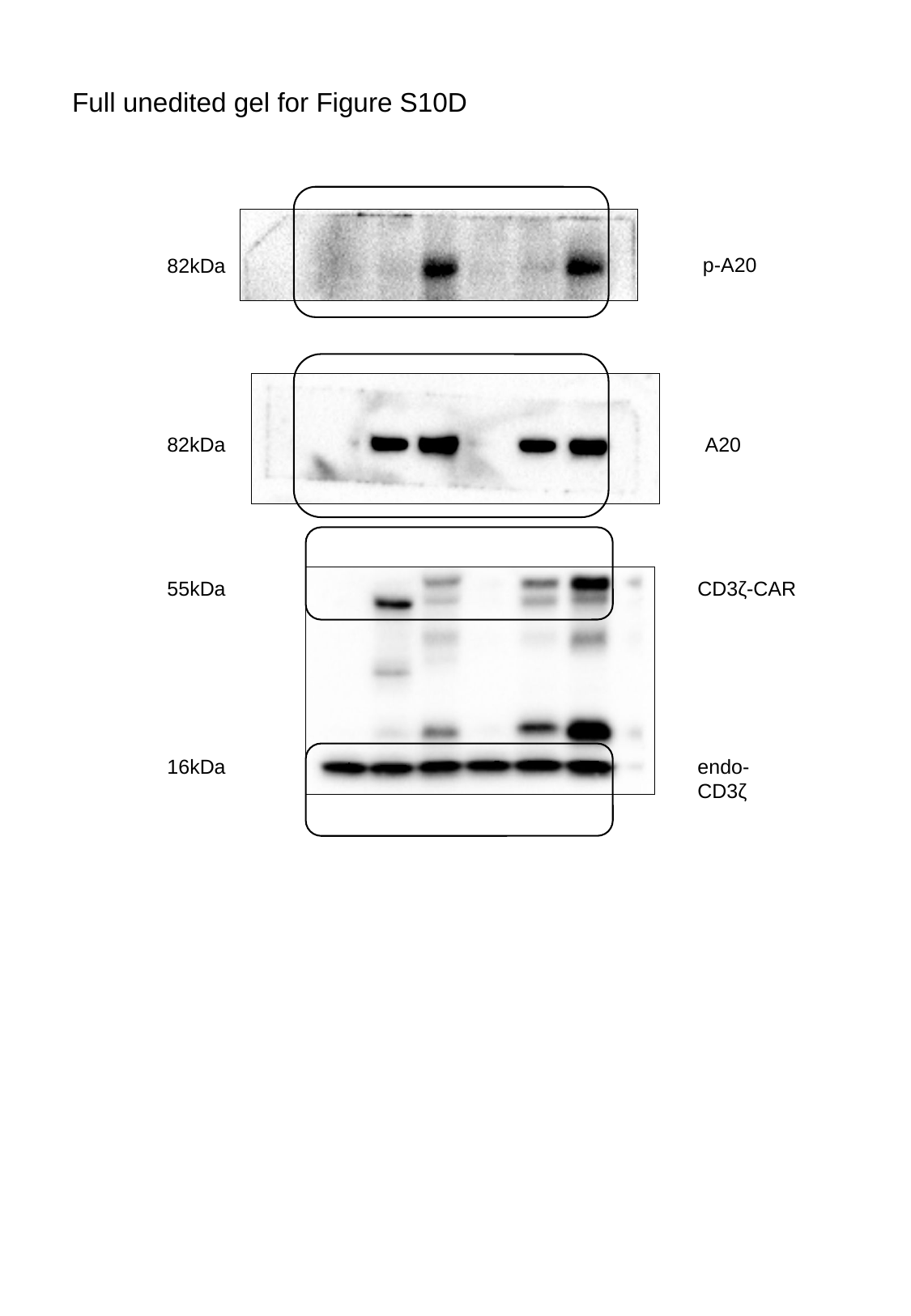

Full unedited gel for Figure S10D
p-A20
82kDa
A20
82kDa
55kDa
CD3ζ-CAR
16kDa
endo-CD3ζ
